# Multitarget drugs as potential therapeutic agents for alzheimer’s disease. A new family of 5-substituted indazole derivatives as cholinergic and BACE1 inhibitors

**DOI:** 10.1080/14756366.2022.2117315

**Published:** 2022-09-01

**Authors:** Pedro González-Naranjo, Concepción Pérez, Marina González-Sánchez, Adrián Gironda-Martínez, Eugenia Ulzurrun, Fernando Bartolomé, Marcos Rubio-Fernández, Angeles Martin-Requero, Nuria E. Campillo, Juan A. Páez

**Affiliations:** a Instituto de Química Médica (CSIC), Madrid, Spain; b Centro de Investigaciones Biológicas Margarita Salas (CSIC), Madrid, Spain; c Centro Nacional de Biotecnología (CSIC), Madrid, Spain; d Centro de Investigación Biomédica en Red de Enfermedades Neurodegenerativas (CIBERNED), Madrid, Spain; e Instituto de Investigación Hospital Doce de Octubre, Madrid, Spain; f Instituto de Ciencias Matemáticas (CSIC), Madrid, Spain

**Keywords:** Alzheimer’s disease, BACE1 inhibitor, BuChE inhibitor, indazole, multitarget drug

## Abstract

Multitarget drugs are a promising therapeutic approach against Alzheimer’s disease. In this work, a new family of 5-substituted indazole derivatives with a multitarget profile including cholinesterase and BACE1 inhibition is described. Thus, the synthesis and evaluation of a new class of 5-substituted indazoles has been performed. Pharmacological evaluation includes *in vitro* inhibitory assays on AChE/BuChE and BACE1 enzymes. Also, the corresponding competition studies on BuChE were carried out. Additionally, antioxidant properties have been calculated from ORAC assays. Furthermore, studies of anti-inflammatory properties on Raw 264.7 cells and neuroprotective effects in human neuroblastoma SH-SY5Y cells have been performed. The results of pharmacological tests have shown that some of these 5-substituted indazole derivatives **1**–**4** and **6** behave as AChE/BuChE and BACE1 inhibitors, simultaneously. In addition, some indazole derivatives showed anti-inflammatory (**3**, **6**) and neuroprotective (**1**–**4** and **6**) effects against Aβ-induced cell death in human neuroblastoma SH-SY5Y cells with antioxidant properties.

## 1. Introduction

The current treatments for Alzheimer’s disease (AD) are unsatisfactory because of their low effectiveness in the acute phase and their adverse side effects. During the last 19 years, a large number of clinical trials have been carried out; however, despite these efforts, no new drug has been approved by the U.S. Food and Drug Administration (FDA)^1^. Recently the FDA approved Aduhelm (aducanumab) via the accelerated approval pathway^2^. However, it appears that Phase III studies are not giving the expected results^3^^,^^4^.

The cognitive impairments suffered by AD patients are associated with cholinergic neurotransmission, which seems to be closely related to the pathological formation of ß-amyloid^5^^,^^6^; therefore, cholinergic drugs like acetylcholinesterase (AChE) inhibitors^7–11^ are currently the most commonly prescribed.

In relation to cholinesterase enzymes, AD is characterised by a significant reduction in AChE activity and an increase in butyrylcholinesterase (BuChE) activity. This increase in BuChE activity, especially found in the hippocampus, might be related to the loss of episodic memory^12^. Moreover, it has been reported that BuChE might facilitate the transformation of an initial benign form of senile plaque to a malignant form associated with AD^13^. On the other hand, BuChE-selective inhibitors have been reported to reduce ß-amyloid (Aß) and ß-amyloid precursor protein (APP) secretion *in vitro* and *in vivo*^14^^,^^15^. The generation of Aß deposits together with the formation of neurofibrillary tangles^16–18^ requires ß-secretase, also know as ß-site amyloid precursor protein (APP)-cleaving enzyme 1 (BACE1), which cleaves APP to release a soluble N-terminal fragment and a membrane-anchored C-terminal fragment^19^^,^^20^.

As the most recent clinical trials of BACE1 inhibitors have not shown great progress^21^, further research and a new strategy are required. Genetic evidence indicates that a gradual deletion of BACE1 not only reverses existing amyloid plaques, but also reduces gliosis and neuritic dystrophy while improving synaptic functions, therefore improving cognitive functions^22^. Thus, BACE1 inhibitors have multiple beneficial effects such as the reduction of Aβ generation and amyloid deposition; additionally, it can also reduce levels of potentially toxic APP-processing products such as the APP intracellular domain (AICD).

It is clear that the BuChE and BACE1 enzymes play an important role in AD and are therefore interesting targets for research into alternative drugs for the treatment of this pathology^12^^,^^13^^,^^23^^,^^24^. Considering that AD is a complex pathology in which a large number of pathways and therapeutic targets are involved^25^, it is reasonable to think that strategies such as multitarget drugs^26–30^ can be a good alternative as an effective treatment to fight this disease.

In this context, continuing with our effort of the advancement of an efficient strategy against AD from a multitarget approach, the development of new BACE1 inhibitors with simultaneous cholinergic properties has been the objective of this work. Thus, we describe the synthesis and biological evaluation of a new family of compounds based on the indazole ether scaffold extending the lateral chain at the 5-position in order to increase interactions with BACE1 receptors.

## 2. Materials and methods

### 2.1. General

Melting points were determined using a MP70 (Mettler Toledo) apparatus and are uncorrected. ^1^H-NMR spectra (300 or 400 MHz) and ^13 ^C-NMR spectra (75 or 100 MHz) were recorded on Varian INNOVA-300 (300 MHz) and Varian MERCURY-400 (400 MHz) spectrometers. The signal of the solvent was used as a reference. The chemical shifts are in ppm. High performance liquid chromatography (HPLC) was performed using a Waters 2695 apparatus with a diode array UV/Vis detector Waters 2996 and coupled to a Waters micromass ZQ using a Sunfire C_18_ column (4.6 × 50 mm, 3.5 µm) at 30 °C, with a flow rate of 0.35 mL/min. The mobile phases used were: CH_3_CN and 0.1% formic acid in H_2_O. Electrospray in positive mode was used for ionisation. The sample injection volume was set to 3 µL of a solution of 1 mg/mL CH_3_CN. Gradient conditions, time of gradient (gt) and time of retention (rt) are specified in each case and a different gradient elution was specified in each case. Automated chromatographic separations were carried out in the Isolera Prime (Biotage) equipment with variable detector, using silica gel 60 (230–400 mesh) cartridges or KP C18-HS cartridges, both from Biotage. Elemental analyses were performed on a Heraeus CHN-O Rapid Analysis apparatus. The purity of all compounds was >95% prior to biological testing (Supporting Information).

Reagents and solvents were purchased from common commercial suppliers, mostly Sigma-Aldrich and Alfa-Aesar, and were used without further purification. 5-nitro-1*H*-3-indazolol (**13**) was prepared from the procedure reported by Pfannstiel^31^, 1-benzyl-5-nitro-3-indazolone (**16**) was prepared from the procedure reported by Palazzo^32^ and 1-benzyl-3-benzyloxy-5-nitroindazole (**14**)^33^, 1-(2-naphthylmethoxy)-3-(2-naphthylmethoxy)-5-nitroindazole (**15**) and 5-amino-1-benzyl-3-(benzyloxy)indazole (**27**) were synthesised using methods described by group^34^.

### 2.2. Chemistry

General procedures for the preparation of 5-piperidinopropylaminoindazole derivatives **1**–**6** and 5-carbonylamino derivatives **7–12**, N-1 substituted 3-indazolol derivatives **17–20**, and 5-aminoindazoles **28–34**, together with spectroscopic characterisation of new compounds can be found in the *Supporting Information*.

### 2.3. Biological assays

#### 2.3.1. *In vitro* cholinesterase inhibition assays

AChE from human erythrocytes (EC 3.1.1.7) (min. 500 units/mg protein in buffered aqueous solution) and BuChE from human serum (EC 3.1.1.8) (3 units/mg protein, lyophilised powder) were purchased from Sigma.

Compounds were evaluated in 100 mM phosphate buffer pH 8.0 at 30 °C, using acetylthiocholine and butyrylthiocholine (0.4 mM) as substrates, respectively. In both cases, 5,5-dithio-bis(2-nitrobenzoic) acid (DTNB, Ellman’s reagent, 0.2 mM) was used and the values of IC_50_ were calculated by UV spectroscopy from the absorbance changes at 412 nm. Experiments were performed in triplicate.

#### 2.3.2. Kinetic study of BuChE inhibition

To investigate the mechanism of action of the compounds on AChE or BuChE, a kinetic analysis was performed. The experiments were carried out using combinations of four substrate concentrations and three inhibitor concentrations. Double-reciprocal Lineweaver–Burk plots of the data obtained, in which each point is the mean of three different experiments, were analysed.

Competitive inhibitors have the same *y*-intercept as uninhibited enzymes (since *V*_max_ is unaffected by competitive inhibitors the inverse of *V*_max_ also doesn’t change), but there are different slopes and *x*-intercepts. Non-competitive inhibition produces plots with the same *x*-intercept as uninhibited enzyme (*K_m_* is unaffected) but different slopes and *y*-intercepts. Uncompetitive inhibition causes different intercepts on both the *y*- and *x*-axes, but the same slope. Mixed inhibitors cause intersects above or below the *x*-axes.

The *K_i_*, values were determined by fitting the kinetic data to a competitive, non-competitive, or mixed inhibition model by nonlinear regression analysis using GraphPad Prism^35^.

#### 2.3.3. The BACE1 enzymatic assay

The BACE1 (EC 3.1.1.8) assay was carried out according to the manufacturer’s described protocol which is available from Invitrogen^36^.

Briefly, BACE1 *in vitro* assays were carried out using fluorescence resonance energy transfer (FRET). An APP-based peptide substrate (rhodamine-EVNLDAEFK-quencher, *K*m of 20 μM) carrying the Swedish mutation and containing rhodamine as a fluorescence donor and a quencher acceptor at each end was used. The intact substrate is weakly fluorescent and becomes highly fluorescent upon enzymatic cleavage. The assays were conducted in 50 mM sodium acetate buffer, pH 4.5, at a final enzyme concentration of 1 U/mL. Inhibitor screening was performed at 10 μM. The mixture was incubated for 60 min at 25 °C under dark conditions and then stopped with 2.5 M sodium acetate. Fluorescence was measured with a FLUOstar Optima (BMG Labtechnologies GmbH, Offenburg, Germany) microplate reader at 545 nm excitation and 585 nm emission. The assay kit was validated by the manufacturer.

#### 2.3.4. Oxygen radical absorbance capacity assay

The ORAC-FL method of Ou et al.^37^, partially modified by Dávalos et al.^38^, was followed, using a FLUOstar Optima (BMG Labtechnologies GmbH, Offenburg, Germany) with 485 excitation and 520 emission filters. 2,2′-Azobis-(amidinopropane) dihydrochloride (AAPH), (±)-6-hydroxy-2,5,7,8-tetramethylchromane-2-carboxylic acid (trolox) and fluorescein (FL) were purchased from Sigma-Aldrich. The reaction was carried out in 75 mM phosphate buffer (pH 7.4) and the final reaction mixture was 200 µL. Antioxidant (25 µL) was dissolved in dimethyl sulfoxide at a concentration of 1 mM and diluted in advance in an assay buffer to the desired concentration; the final DMSO concentration in the reaction mixture did not exceed 1% and FL (150 µL; 10 nM) solutions were placed in a black 96-well microplate (96 F untreat, NuncTM). The mixture was pre-incubated for 30 min at 37 °C and then AAPH solution (25 µL, 240 mM) was added rapidly using a multichannel pipette. The microplate was immediately placed in the reader and the fluorescence was recorded every 90 s. for 90 min. The microplate was automatically shaken prior to each reading. Samples were measured at four different concentrations (10^−1 ^µM). A blank (FL + AAPH in phosphate buffer) instead of the sample solution and four calibration solutions using trolox (10^−1 ^µM) was also used in each assay. All of the reaction mixtures were prepared in duplicate and at least three independent assays were performed for each sample. Raw data were exported from the Fluostar optima Software to an Excel sheet for further calculations. Antioxidant curves (fluorescence *vs.* time) were represented and the area under the curve (AUC) is calculated as:

$$\text{AUC }=f_{\text{1}/}f_{0}+f_{i/}f_{0}+\;\ldots \;+f_{\text{34}}/f_{0}+\;\left(f_{\text{35}}/f_{0}\right)$$

where *f*_o_ = initial fluorescence reading at 0 min and *f_i_* = fluorescence reading at time i. The net AUC is obtained by subtracting the AUC of the blank from that of a sample. The relative Trolox equivalent ORAC value is calculated as:

Relative  ORAC  value =[(AUC sample - AUC blank)/(AUCT rolox - AUC blank)](molarity  of  trolox/molarity of sample)

#### 2.3.5. Nitrite determinations

The content of nitrite, one of the end-products of NO oxidation, was monitored by a procedure based on the diazotidation of nitrite by sulphanilic acid (Griess reaction). Twenty-four hours after the incubation of Raw 264.7 cells with 0.4 µg/mL of LPS, 50 µL of sample aliquots were mixed with 50 µL of Griess reagent in 96-well plates and incubated at room temperature for 15 min. The absorbance (520 nm) of the mixture was measured on a microplate reader. The concentration of nitrite was calculated with the linear equation derived from the standard curve generated by known concentrations of sodium nitrite.

#### 2.3.6. Amyloid-beta peptide neurotoxicity

SH-SY5Y human neuroblastoma cells were obtained from American Type Culture Collection. Cells were maintained at 37 °C in a 5% CO2 humidified atmosphere in DMEM-Glutamax supplemented with heat-inactivated 10% foetal bovine serum and 1% penicillin/streptomycin. All experiments with Aβ1–42 used DMEM-Glutamax without phenol in the media.

The compounds were dissolved in 100% dimethyl sulphoxide (DMSO) as a 10 mM stock and diluted with culture medium to final concentrations. Aβ1–42 powder (AnaSpec, Inc., San Jose, CA) was dissolved in acetic acid (0.1 M) obtaining a 2 μg/μL stock. Then Aβ1-42 was oligomerised in no phenol red DMEM for 24 h at 37 °C. The final concentration in cells cultures was 5 μM. All dilutions of stock were prepared fresh before addition to the culture medium.

SH-SY5Y cells were treated with 5 μM Aβ1-42 for 24 h and pre-treated or not for 1 h with increasing concentrations of compounds. After treatment, cultures were processed for a cell viability assay using MTT assay.

#### 2.3.7. Cell viability assay

Cell viability was measured using the 3-(4,5-Dimethylthiazol-2-yl)-2,5-diphenyltetrazolium bromide (MTT) assay, based on the ability of viable cells to reduce yellow MTT to blue formazan. Briefly, cells cultured in 96-well plates and treated with the indicated compounds for 16 h were incubated with MTT (0.5 mg/ml, 4 h) and subsequently solubilised in DMSO. The extent of reduction of MTT was quantified by absorbance measurement at 595 nm according to the manufacturer’s protocol.

## 3. Results and discussion

Based on previous results, one possible way to develop BACE1 inhibitors consists of introducing different functional groups in position 5 of the indazole structure. Thus, different functionalised groups such as indazolyl benzamides, indazolyl ureas and 5-piperidinopropylaminoindazole derivatives were chosen. In the other two positions of the of the indazole system, *N-1 and O-3*, different groups were also included to achieve different families and therefore chemical diversity. A variety of aromatic groups such as benzyl, 4-chlorobenzyl, 2,3-dichlorobenzyl, 3,4-dichlorobenzyl, 1-naphthylmethyl and 2-naphthylmethyl were selected as substituents at position 1 and oxygen at position 3 of indazole ring.

### 3.1. Chemistry

**Scheme 1.**
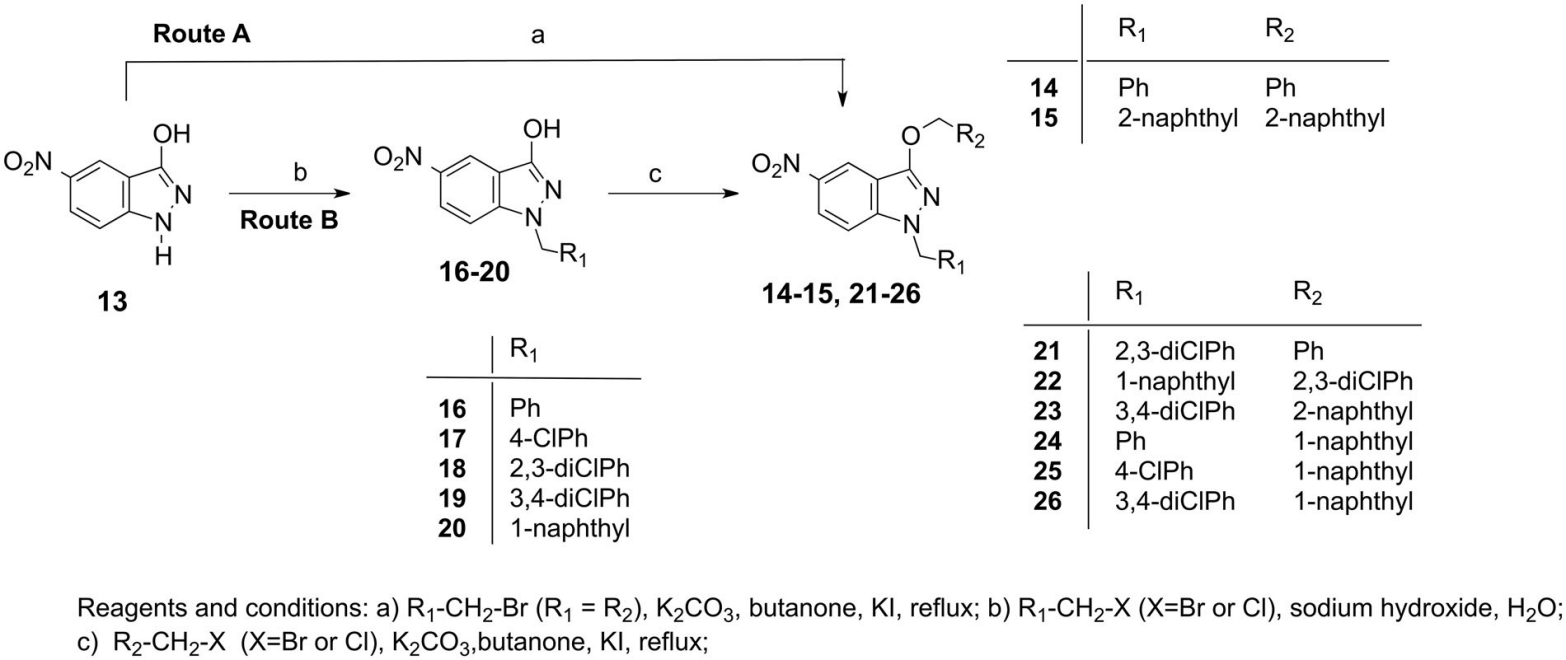
Synthetic routes for the preparation of 5-nitroindazole derivatives **14**–**26**.

**Scheme 2.**
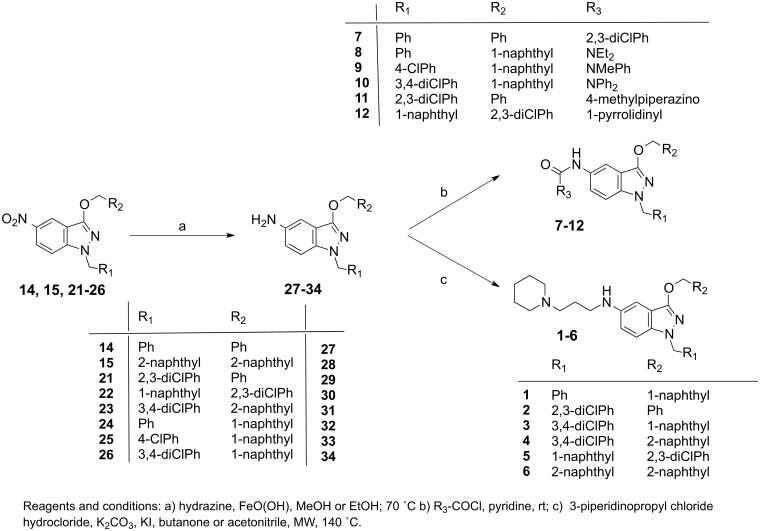
Synthetic routes for the preparation of 5-aminosubstituted indazole derivatives **1**–**12.**

Representative sets of compounds (**1–12**) were proposed as potential candidates (Table 1). The synthetic methodology for accessing the target compounds **1–12** was based on the introduction of the different groups at N-1 position or at the hydroxyl group of the 5-nitroindazolol **13** through well-established *N*-alkylation or *O*-alkylation procedures^34^ (Scheme 1) and further transformation of the nitro group at position 5 of the indazole ring (Scheme 2).

**Table 1.** Inhibition of AChE, BuChE, BACE1 (IC_50_, μM) and antioxidant activity (ORAC) of selected indazole derivatives and inhibition type of BuChE inhibitors.

compd	*R*_1_	*R*_2_	*R*_3_	AChE^a^ IC_50_ (µM)	BuChE^a^ IC_50_ (µM)	Type of Inhibition (BuChE)^b^	BACE1^c^ IC_50_ (µM)	ORAC^d^ (µM)
1	Ph	1-naphthyl	NH(CH_2_)_3_-piperidino	>10 (23 %)	3.2 ± 0.5	M	2.7 ± 0.1	1.1 ± 0.1
2	2,3-diClPh	Ph	NH(CH_2_)_3_-piperidino	>10 (27 %)	0.40 ± 0.04	M	3.2 ± 0.2	0.7 ± 0.1
3	3,4-diClPh	1-naphthyl	NH(CH_2_)_3_-piperidino	>10 (38%)	0.17 ± 0.05	M	2.1 ± 0.2	0.6 ± 0.1
4	3,4-diClPh	2-naphthyl	NH(CH_2_)_3_-piperidino	>10 (26%)	0.57 ± 0.2	M	1.9 ± 0.1	0.7 ± 0.1
5	1-naphthyl	2,3-diClPh	NH(CH_2_)_3_-piperidino	>10	>10		2.7 ± 0.1	0.8 ± 0.1
6	2-naphthyl	2-naphthyl	NH(CH_2_)_3_-piperidino	9 ± 1	3.7 ± 0.3	M	3.4 ± 0.2	0
7	Ph	Ph	NHCO(2,3-diClPh)	7.6 ± 0.3	>10		(60%)	0
8	Ph	1-naphthyl	NHCO(NEt_2_)	>10 (29 %)	>10		9.1 ± 0.5	0.9 ± 0.1
9	4-ClPh	1-naphthyl	NHCON(Ph,Me)	>10	>10		>10 (29%)	0.6 ± 0.1
10	3,4-diClPh	1-naphthyl	NHCON(Ph)_2_	>10 (28 %)	>10		>10 (10%)	0.2 ± 0.1
11	2,3-diClPh	Ph	NHCO(4-methylpiperazino)	>10	>10 (47%)		2.8 ± 0.5	1.1 ± 0.1
12	1-naphthyl	2,3-diClPh	NHCO(1-pyrrolidinyl)	>10	>10		>10 (43%)	0.5 ± 0.1
27	Ph	Ph	NH_2_	8.6 ± 0.5	>10 (21%)		>10 (2%)	0.9 ± 0.1
28	2-naphthyl	2-naphthyl	NH_2_	4.1 ± 0.2	>10 (24%)		8.9 ± 0.5	0.3 ± 0.1
29	2,3-diClPh	Ph	NH_2_	>10	>10		>10	0.7 ± 0.1
30	1-naphthyl	2,3-diClPh	NH_2_	>10 (36 %)	>10		(79%)	0.8 ± 0.1
31	3,4-diClPh	2-naphthyl	NH_2_	6.0 ± 0.5	>10 (30%)		(69%)	0.63 ± 0.04
32	Ph	1-naphthyl	NH_2_	>10	>10		>10	1.0 ± 0.1
33	4-ClPh	1-naphthyl	NH_2_	>10	>10		5.8 ± 0.5	1.0 ± 0.1
34	3,4-diClPh	1-naphthyl	NH_2_	3.5 ± 0.6	>10 (32%)		(72%)	0.33 ± 0.05

^a^ IC50 values (mean ± SEM) were determined from 3 different experiments using acetylthiocholine and butyrylthiocholine (0.8 and 0.5 µM, respectively) as substrates. In parentheses: percentage of inhibition at 10 µM.

^b^ BuChE inhibition type, M: mixed.

^c^ IC50 values (mean ± SEM). In parentheses: percentage of inhibition at 10 µM.

^d^ ORAC: Oxygen Radical Absorbance Capacity. Data are expressed as μmol of Trolox equivalents/μmol of tested compound.

The reaction of the starting 5-nitroindazole **13** with alkyl halides under refluxing of butanone solvent (route A, Scheme 1) was useful to obtain disubstituted derivatives with identical groups at both N- and O-positions (**14**, **15**). For derivatives with different groups at N-1 and OH at C3, route B was applied (Scheme 1).

This synthetic route consists of a two-step sequence involving initial alkylation with the suitable benzylic halide at the more nucleophilic N-1 position of the indazole derivative and then introduction of the second substituent at position 3-OH using different benzylic halides.

Following **route A**, when the indazole ether derivative has the same substituent at both the *N-*1 and *O-3* positions, the reaction was carried out in potassium carbonate and potassium iodide in butanone. Thus, the preparation of the 1-substituted benzyl and naphthyl indazole ethers **14** and **15** were carried out starting from 5-nitroindazolol **13** and benzyl bromide and naphthyl bromide^34^, respectively (Scheme 1).

The synthesis of 1,3-disubstituted indazoles with different groups at N-1 position and ether function were performed following a more versatile synthetic **route B**, starting from 5-nitroindazolol **13** and the corresponding halides in an aqueous solution of sodium hydroxide.

Thus, the reaction of **13** with the corresponding bromides or chlorides afforded the N-1 benzyl, 4-chlorobenzyl and 2,3-dichlorobenzyl **16**–**18** and the 3,4-dichlorobenzyl and 1-naphthylmethyl 3-indazolol derivatives **19–20**, respectively. Subsequent O-alkylation of **16**–**20** with the corresponding bromides of benzyl, 2,3-dichlorobenzyl and 2-naphthylmethyl and the 1-naphthylmethyl chloride afforded the indazole ethers of benzyloxy **21**, 2,3-dichlorobenzyloxy **22**, 2-naphthylmethoxy **23** and 1-naphthylmethoxy **24–26**, respectively.

For the preparation of 5-aminoindazole ethers, we performed novel modifications in the indazole ether system via the reduction of 5-nitroindazoles (*Scheme 2*). In this sense, the conversion of the nitro group at C-5 into the corresponding 5-amino derivative was achieved by treatment with hydrazine and ferric oxyhydroxide FeO(OH) as a catalyst^34^. The FeO(OH) was prepared by the treatment of FeCl_3_ and sodium hydroxide solution^39^. Thus, the reduction of the 5-nitro derivatives **14**, **15** and **21–26** afforded the corresponding 5-amino derivatives **27**–**34**, respectively.

The more interesting family selected, the 5-piperidinopropylaminoindazole derivatives, were prepared from the corresponding 1,3-disubstituted 5-aminoindazoles with piperidinopropyl chloride. Thus, the synthesis of indazole ethers **1**–**6** was carried out starting from the 5-amino-1-substituted derivatives of benzyl **29**, 2,3-dichlorobenzyl **30**, 1-naphthyl **32** and **34** and 2-naphthylmethyl ethers **28** and **31**.

Finally, the second selected family, the indazolyl carboxamides **7**–**12**, was analysed through the reaction of 1,3-disubstituted 5-aminoindazoles with acyl chloride. Thus, the indazolyl-2,3-dichlorobenzamide **7** was obtained by the reaction of the 5-aminoindazole **27** with the corresponding acyl halide in the presence of pyridine.

The indazolyl urea derivatives were prepared in a similar way. The reaction of 3-naphthylmetoxy derivatives **32**–**34** with diethylcarbamoyl chloride, N-Methyl-N-phenylcarbamoyl chloride and diphenylcarbamyl chloride afforded the corresponding indazolyl ureas **8**–**10**, respectively. Finally, the piperazinyl and pyrrolidinyl carboxamides **11** and **12** were prepared from **29** and **30** with the 4-methyl-1-piperazinecarbonyl chloride and the 1-pyrrolidinecarbonyl chloride, respectively.

The structures of synthesised compounds (**1**–**12**, **14**–**35**) were established as N1 and N-1/O3 disubstituted indazoles on the basis of NMR data (Supplemental Tables S1 and S2). The position of the 1 and 3 substituents can be clearly distinguished by ^13 ^C-NMR spectra examining the chemical shifts (supplemental Table S2). Thus, the signals corresponding to aromatic methylene group attach at O-3 (68.5–71.8 ppm) appear at lower fields in relation to aromatic methylene at N-1 (51.1–53.0 ppm).

### 3.2. Biological assays

#### 3.2.1. Ache/BuChE inhibitory activity

The derivatives corresponding to final families **1**–**12** and their precursors, the 5-aminoindazoles **27**–**34** were subjected to enzymatic assays^40^ in order to evaluate their capacity to inhibit human AChE and BuChE. Those compounds that inhibited >50% had their IC_50_ calculated. The experimental values, both % inhibition and IC_50_, are shown in Table 1. Unfortunately, not all quantitative values of IC_50_ could be determined for solubility reasons.

In relation to 5-aminoindazoles **27**–**34**, regarding AChE inhibition, compounds **27**, **28**, **31** and **34** showed enzymatic inhibition with IC_50_ values less than 10 µM. However, in relation to BuChE inhibition, neither compound showed interesting activity, although the compounds that behave as BuChE inhibitors (**27**, **28**, **31** and **34**) showed a very slight inhibition at 10 µM.

The results obtained for AChE inhibition of target indazoles (**1**–**12**) indicate that several derivatives have significant activity as inhibitors of this enzyme, with compounds **6** and **7** showing lower IC_50_ values among the 5-*substituted* indazoles.

However, the most relevant activity was found in relation to BuChE enzymatic assays, with the 5-amino*substituted* indazoles **1**–**4**, **6** and **11** being the most interesting compounds in relation to the inhibition of BuChE. It should be noted that the N-1 dichlorobenzyl derivatives **2**–**4** showed the lowest IC_50_ values, in the submicromolar range, among the piperidinopropylamino derivatives.

According to values of inhibitory activity for BuChE and AChE, the most interesting compounds in BuChE enzymatic inhibition are derivatives **2**–**4**, which showed lower activities in AChE. The naphthyl derivative **6** is the only compound that showed activity in both enzymes in the micromolar range.

#### 3.2.2. Kinetic study of BuChE inhibition

In order to obtain better knowledge about the mechanism of action of this family of compounds, a kinetic study was carried out with the most promising BuChE inhibitors. Lineweaver–Burk plots were used for the indazole derivatives **1**–**4** and **6**, with donepezil as a reference compound; these are shown in supplementary material (supplemental Fig. S1) and the data gathered in Table 1.

The IC_50_ for BuChE inhibitors (Table 1) was determined by fitting the kinetic data to a competitive, non-competitive, or mixed inhibition model by nonlinear regression analysis using GraphPad Prism 5^35^.

Regarding the inhibition type of BuChE, Lineweaver–Burk plots obtained for **1**–**4** and **6** (supplemental Figure S1) showed both increasing slopes (decreased at increasing inhibitor concentrations) and increasing intercepts with a higher inhibitor concentration and similar plots to those shown by donepezil and therefore according to a mixed type competitive BuChE inhibition mode of action. Kinetic studies suggested two potential different sites of interaction: the active site and the peripheral binding site.

#### 3.2.3. BACE1 enzymatic assay

The BACE1 assay was carried out according to the described protocol available from Invitrogen^36^. The percentages of inhibition for BACE1 were determined for all 5-aminoindazole ethers **27**–**34**, 5-piperidinopropylamine **1**–**6**, indazolyl benzamide **7** and indazolyl ureas **8**–**12** at 10 µM (Table 1).

Those compounds that inhibit 100% up to 10 µM were selected for the determination of IC_50_ and consideration for later studies (Table 1). The results presented in Table 1 indicate that only the 5-amino derivatives **28** and **33** showed activity as inhibitors of BACE1. In relation to the selected family of indazolyl carboxamides **7**–**12**, only the indazolyl derivative **8** and the piperazinyl carboxamide derivative **11** showed activity as inhibitors of BACE1. The most interesting results correspond to all indazoles bearing the 5-piperidinopropylamine substituent **1**–**6**, which showed the lowest IC_50_ values, and 5-piperazinocarboxamide **11**.

The analyses of the results gathered in Table 1, according to values of inhibitory activity for AChE, BuChE and BACE1 enzymes, indicate that the most interesting compounds selected to study their anti-inflammatory and neuroprotective effects were the multitarget derivatives **1**–**4** and **6**, which behave as BACE1 inhibitors with simultaneous activity as AChE/BuChE inhibitors.

#### 3.2.4. Antioxidant activity

The antioxidant activities of the indazole ether derivatives **1**–**12** and **27**–**34** were evaluated by following the well-established ORAC-FL method (oxygen radical absorbance capacity by fluorescence)^37^^,^^38^.

The antioxidant activity shown by the amino indazole derivatives is essentially unchanged in the corresponding 5-amino substituted indazoles, except for compound **7** which lost the effect.

In relation to the most interesting compounds **1**–**12**, the results shown in Table 1 indicate that most indazole derivatives exhibit antioxidant properties except compound **6** and **7**. The 5-piperidinopropilaminoindazole derivatives **1**–**5**, carboxamides **8**, **9** and the piperazinocarbonylamino derivative **11** showed antioxidant properties with values higher than 60% in relation to Trolox. It is worth highlighting that compounds **1** and **11** protect against free radicals in a similar manner as the reference compound Trolox.

#### 3.2.5. Anti-inflammatory effect of selected indazole derivatives

Among the most commonly employed methods to evaluate anti-inflammatory responses is the LPS-treated Raw 264.7 murine macrophage model. The exposure of Raw 264.7 macrophages to external bacterial toxins like lipopolysaccharide (LPS) has been extensively shown to stimulate the secretion of nitric oxide (NO), which is produced by the inducible isoforms of nitric oxide synthase (iNOS)^41^. Thus, many studies have evaluated the effects of anti-inflammatory agents on the direct inhibition of NO production^42^.

An analysis of the results (Table 1), according to values of inhibitory activity for AChE, BuChE and BACE1, indicates that the most interesting compounds selected for the study of inflammation and neuroprotective effects should be the multitarget derivatives **1**–**4** and **6**, which behave as BACE1 inhibitors with simultaneous activity as AChE/BuChE inhibitors.

**Figure 1.**
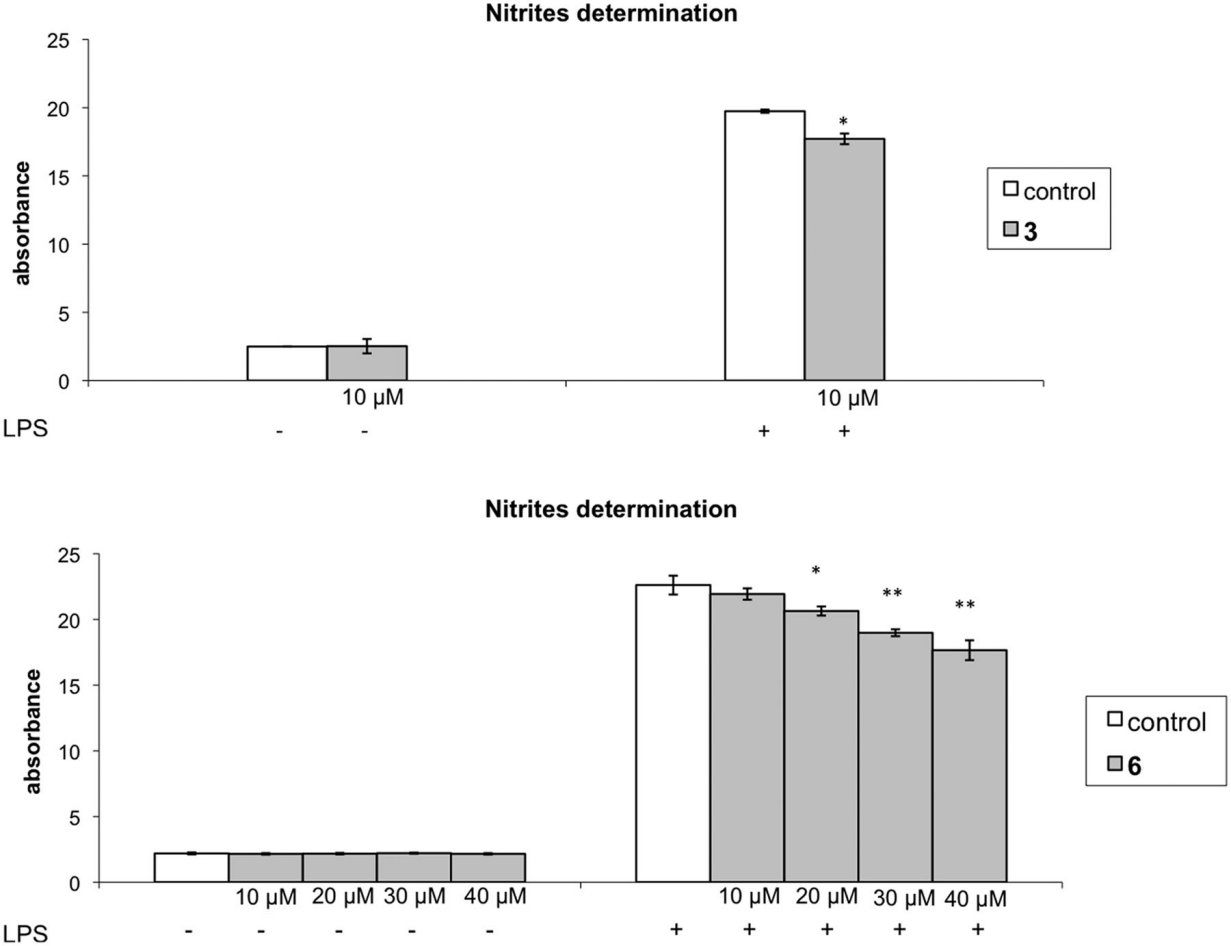
Anti-inflammatory effects of compounds **3** and **6.** The production of extracellular nitrite in Raw 264.7 cells stimulated with LPS (0.4 µg/mL) for 24 h with and without compounds **3** (10 µM) and **6** (10–40 µM). Data are expressed as the mean ± SD from two independent experiments and quantified using Griess reagent. **p < 0.01, *p < 0.1 significantly different from LPS-treated cells.

The anti-inflammatory effects of selected 5-substituted indazole derivatives were evaluated from the study of the NO production on cells exposed to LPS and the relevant results are represented in the Figure 1. The effect of indazole derivatives **1**–**4**, **6** on murine Raw 264.7 cells viability was examined. The assays to 10 µM reveal that **1**, **2** and **4** show cytotoxicity, while **3** (10 µM) and **6** (10, 20, 30, 40 µM) did not show any significant cytotoxic effect.

When control cells were exposed to LPS, the piperidinopropylamino derivatives **3** and **6** were able to attenuate LPS-induced NO production at 10 µM, while **6** inhibited NO production at 20, 30 and 40 µM.

#### 3.2.6. Neuroprotective effect of indazole derivatives on β-Amyloid-Induced death in AD neuronal models

Aβ peptide is toxic to cells both *in vivo* and *in vitro*^43^^,^^44^, inducing the death of human neuroblastoma SH-SY5Y cells. Thus, the human neuroblastoma SH-SY5Y cells^45^ are used as *in vitro* models of neuronal function and differentiation being one of the most common cell line used *in vitro* for biochemical and toxicological studies of Aβ1-42 amyloid peptide^46^.

**Figure 2.**
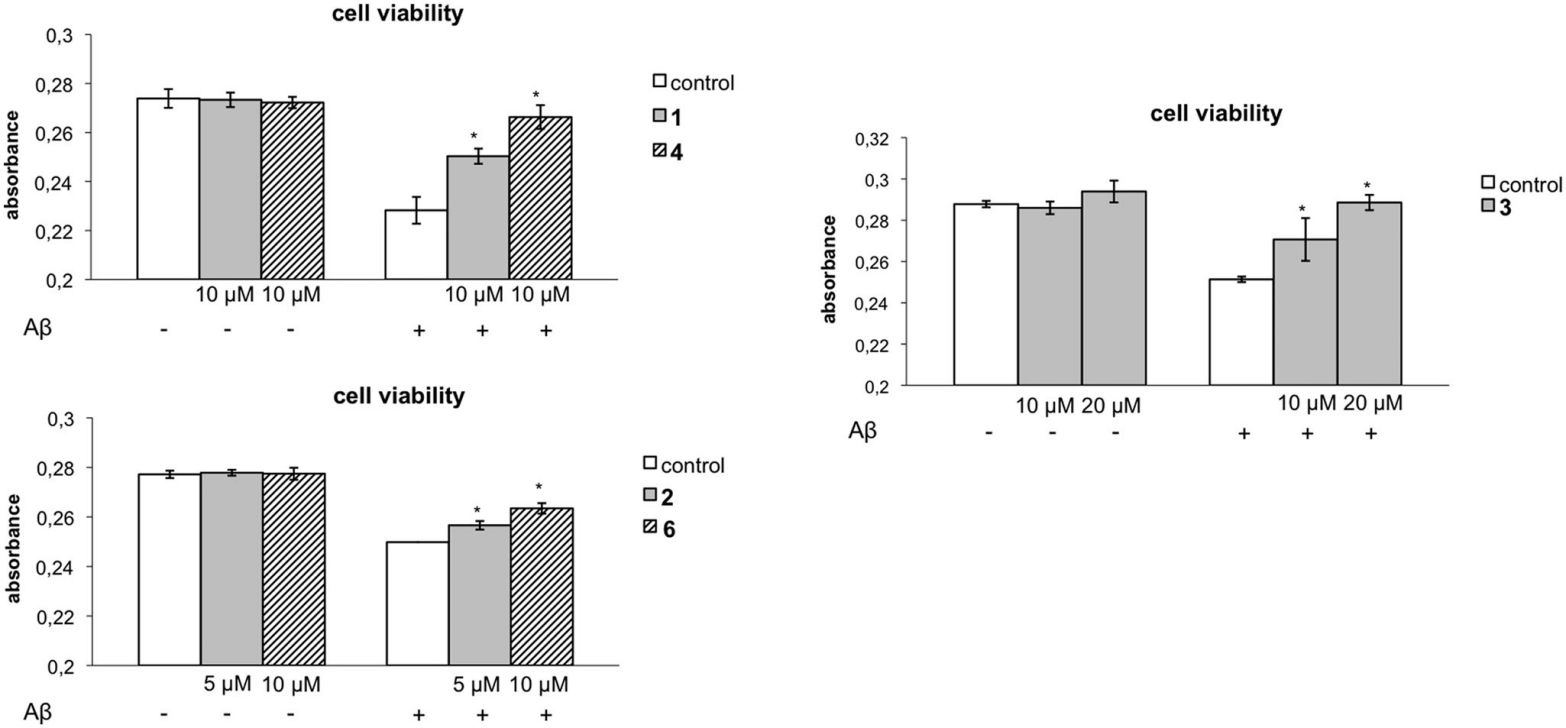
Neuroprotective effects of compounds **1**–**4** and **6** on β-amyloid (Aβ)-induced death in neuronal cells. Neuroblastoma SH-SY5Y cells were incubated in the absence or presence of 5 µM Aβ for 24 h with and without compounds **1**, **4**, **6** (10 µM), **2** (5 µM) and **3** (10 and 20 µM) (maximum non-toxic concentrations) added 1 h prior to Aβ incubation. The number of viable cells after drug treatments was measured by the 3–(4,5-dimethylthiazol-2-yl)-2,5-diphenyltetrazoliumbromide assay. Each data point represents the mean ± standard error of the mean for four different experiments. **p < 0.01, *p < 0.1 significantly different from Aβ-treated cells.

The effect of the most interesting derivatives **1**–**4** and **6** has been studied from the survival of Aβ-treated SH-SY5Y cells and the results are shown in Figure 2. Two conclusions can be drawn from the results: (i) the derivatives do not show cytotoxicity at the concentration studied (Figure 2**)**; and (ii) as expected, Aβ-induced death of SH-SY5Y cells (Figure 2**)** and the indazole derivatives **1**, **3**, **4** and **6** tested significantly reduced cell death at 10 µM, meanwhile compound **2** is able to prevent cell death at a lower concentration, 5 µM.

These results could be explained in a similar way to the effect of galanthamine against Aβ1-42-induced genotoxic and cytotoxic damages in the SH-SY5Y cell line. Thus, this compound may exert antigenotoxic properties and regulate cell loss in addition to effects as an AChE inhibitor and antioxidant activity^47^.

## 4. Conclusions

The aim of this research was to identify and propose a valid multitarget approach as a potential therapeutic strategy to fight against diseases characterised by inflammatory and neurodegenerative symptoms such as Alzheimer’s disease. Thus, we developed a new series of indazole derivatives with a multitarget profile, being inhibitors of ChE (AChE/BuCHE) and/or BACE-1 enzymes.

The piperidinopropylaminoindazole derivatives have shown the most interesting properties since they behave as simultaneous AChE/BuChE and BACE1 inhibitors. In relation to this series, the derivatives **1–4** and **6** should be emphasised according to neuroprotective effect.

Importantly, the same derivative that behaves as an AChE/BuChE/BACE1 inhibitor, compound **3**, was shown to exhibit anti-inflammatory and neuroprotective effects with antioxidant properties. All of these results together highlight the great potential of this new family as a lead compound for the development of drugs as potential treatments for AD.

## Supplementary Material

Supplemental Material
