# Supplementary material for: Multitarget drugs as potential therapeutic agents for alzheimer’s disease. A new family of 5-substituted indazole derivatives as cholinergic and BACE1 inhibitors
Source: J Enzyme Inhib Med Chem. 2022 Sep 1;37(1):2348–56. doi: 10.1080/14756366.2022.2117315 (PMC9477487; doi:10.1080/14756366.2022.2117315)
Supplement: Supplemental Material [file IENZ_A_2117315_SM8120.pdf]

Multitarget Drugs as Potential Therapeutic Agents for Alzheimer's Disease. A New Family of 5-Substituted Indazole Derivatives as Cholinergic and BACE1 Inhibitors

Pedro González-Naranjo[a], Concepción Pérez[a], Marina González-Sánchez[a], Adrián Gironda-Martínez[a], Eugenia Ulzurrun[b,c], Fernando Bartolomé[d,e], Marcos Rubio-Fernández[d], Angeles Martin-Requero[b,e], Nuria E. Campillo[b,f*] and Juan A. Páez[a*]

[a] *Instituto de Química Médica (CSIC), Juan de la Cierva 3, 28006-Madrid, Spain.*

[b] *Centro de Investigaciones Biológicas Margarita Salas (CSIC), Ramiro de Maéztu 9, 28040-Madrid, Spain.*

[c] *Centro Nacional de Biotecnología (CSIC), Darwin 3, 28049-Madrid, Spain.*

[d] *Centro de Investigación Biomédica en Red de Enfermedades Neurodegenerativas (CIBERNED), Valderebollo 28031, Madrid, Spain*

[e] *Instituto de Investigación Hospital Doce de Octubre, Avda de Córdoba s/n 28041 Madrid, Spain.*

[f] *Instituto de Ciencias Matemáticas (CSIC), Nicolás Cabrera, nº13-15, Campus de Cantoblanco, UAM, 28049-Madrid, Spain.*

Contents:

1. Chemistry. Procedures for the preparation of new indazole derivatives **1-12**, **17-26**, and **28-34**.

Table S1: ^{1}H-NMR spectral data (δ) of compounds **1-12**, **21-26**, **28-34**.

Table S2: ^{13}C-NMR spectral data (δ) of compounds **1-12**, **21-26**, **28-34**.

Figure S1. Kinetic study on the mechanism of BuChE inhibition of compounds **1-4** and **6**.

Figures S2-S25: Representative ^{1}H and ^{13}C-NMR spectra of compounds **1-12**.

2. Docking Studies. 2D interaction of compounds **31** and **4.**

Figure S26. 2D interaction plot of compounds (a) **31** and (b) **4**.

Table S3. Interactions of 31 and 4 in the binding site of BACE1.

Elemental Analysis Data.

[a*] Instituto de Química Médica (CSIC). Juan de la Cierva, 3, 28006-Madrid, Spain. jpaez@iqm.csic.es. Tel.: +34 915622900

[b*] Centro de Investigaciones Biológicas Margarita Salas (CSIC). Ramiro de Maeztu, 9, 28040-Madrid, Spain. nuria.campillo@csic.es. Tel.: +34 918373112

1. Chemistry

***1.1. General procedure for the synthesis of piperidinopropylaminoindazole derivatives* 1-6**. To a solution of the appropriate 1,3-disubstitiuted 5-aminoindazole derivative in butanone or acetonitrile, K_2CO_3, catalytic amount of KI and 3-piperidinopropyl chloride hydrochloride were added. The mixture reaction was heated at 140ºC and stirred in microwave reactor. Then, the reaction mixture was cooled and filtered to remove existing inorganic salts. The solvent was evaporated under reduced pressure and purified. Amounts of reagents, time reaction, conditions, specific procedures and purification methods are specified in each case.

1.2. 1-benzyl-3-(1-naphthylmethoxy)-5-(3-piperidinopropylamino)indazole (**1**)**.** From **32** (0.15 g, 0.39 mmol), 3-piperidinopropyl chloride hydrochloride (0.16 mg, 0.79 mmol) and K_2CO_3 (0.27 g, 2.0 mmol) in butanone (6 mL). Reaction time: 12 h. The product was purified by flash chromatography using as eluent mixture CH_2Cl_2/methanol (40/60). Yield: (0.02g, 13%). Oil. ^{1}H-NMR (400 MHz, acetone-d_6) δ: 8.22 (d, 1H, Ar); 7.99 – 7.92 (m, 2H, Ar); 7.74 (dd, 1H, Ar); 7.59 – 7.48 (m, 3H, Ar); 7.31 – 7.19 (m, 6H, 7-H, Ar); 6.83 (dd, 1H, 6-H); 6.54 (d, 1H, 4-H); 5.86 (s, 2H, OCH_2); 5.40 (s, 2H, N1-CH_2); 3.08 (t, 2H, NH-$\underline{CH_2}$); 2.40 – 2.36 (m, 6H, $\underline{CH_2}$-N, N($\underline{CH_2}$)$_2$); 1.75 (q, 2H, $CH_2\underline{CH_2}CH_2$); 1.52 (bs, 4H, $N(CH_2)_2(\underline{CH_2})_2$); 1.37 (m, 2H, CH_2). ^{13}C-NMR (100 MHz, acetone-d_6) δ: 155.6 (C-3); 144.2 (C-5); 139.4 (C-7a); 120.0 (C-6); 114.6 (C-3a); 110.9 (C-7); 97.5 (C-4); 69.5 (OCH_2); 58.2 (NH-CH_2); 55.3 (2C, $N(CH_2)_2$); 52.8 (N1-CH_2); 44.1 (CH_2N); 26.7 (2C, $N(CH_2)_2(\underline{CH_2})_2$); 26.5 ($CH_2\underline{CH_2}CH_2$); 25.1 ($CH_2$); 137.6 (Ar); 134.8 (Ar); 134.1 (Ar); 132.9 (Ar); 129.7 (Ar); 129.4 (Ar); 129.2 (2C, Ar); 128.2 (Ar); 128.1 (2C, Ar); 128.0 (Ar); 127.2 (Ar); 126.7 (Ar); 126.2 (Ar); 124.9 (Ar). HPLC-MS (ES$^+$): CH_3CN/H_2O 15:85–95:15, gt: 10.00 min; rt: 6.46 min, $[M+H]^+$ = 505.

1.3. 3-(benzyloxy)-1-(2,3-dichlorobenzyl)-5-(3-piperidinopropylamino)indazole (**2**)**.** From **29** (0.15 g, 0.37 mmol), 3-piperidinopropyl chloride hydrochloride (0.19 mg, 0.94 mmol) and K_2CO_3 (0.31 g, 2.0 mmol) in butanone (6 mL). Reaction time: 16 h. The product was purified by flash chromatography using as eluent mixture CH_2Cl_2 / methanol (40/60). Yield: (0.022g, 11%). Oil. ^{1}H-NMR (400 MHz, acetone-d_6) δ: 7.54 -7.51 (m, 2H, 7-H, Ar); 7.47 (d, 2H,Ar); 7.40 – 7.30 (m, 3H, Ar); 7.22 (dd, 1H, Ar); 7.17 (m, 2H, 7-H, Ar); 6.89 (dd, 1H, 6-H); 6.65 (d, 1H, 4-H); 6.63 (dd, 1H, Ar). 5.49 (s, 2H, OCH_2); 5.38 (s, 2H, N1-CH_2); 3.18 (t, 2H, NH-$\underline{CH_2}$); 2.54 – 2.50 (m, 6H, $\underline{CH_2}$-N, N($\underline{CH_2}$)$_2$); 1.86 (q, 2H, $CH_2\underline{CH_2}CH_2$); 1.62 (m, 4H, $N(CH_2)_2(\underline{CH_2})_2$); 1.45 (m, 2H, CH_2). ^{13}C-NMR (100 MHz, acetone-d_6) δ: 155.1 (C-3); 143.6 (C-5); 138.7 (C-7) 119.4 (C-6); 113.6 (C-3a); 109.8 (C-7); 96.6 (C-4); 70.1 (OCH_2); 57.3 (NH-CH_2); 54.4 (2C, $N(CH_2)_2$); 49.8 (N1-CH_2); 43.2 (CH_2N); 25.8 (2C, $N(CH_2)_2(\underline{CH_2})_2$); 25.7 ($CH_2\underline{CH_2}CH_2$); 24.3 ($CH_2$); 137.6 (Ar); 136.9 (Ar); 132.4 (Ar); 129.2 (Ar); 128.3 (Ar); 128.1 (Ar); 127.9 (Ar); 127.8 (Ar); 127.0 (Ar). HPLC-MS (ES$^+$): CH_3CN/H_2O 15:85–95:5, gt: 10.00 min, rt: 6.69 min, $[M+H]^+$ =523.

1.4. 1-(3,4-dichlobenzyl)-3-(1-naphthylmethoxy)-5-(3-piperidinopropylamino)indazole (**3**). From **34** (0.10 g, 0.22 mmol), 3-piperidinopropyl chloride hydrochloride (0.09 mg, 0.44 mmol) and K_2CO_3 (0.17 g, 1.2 mmol) in CH_3CN (10 mL). Reaction time: 8 h. The product was purified by flash chromatography using

as eluent mixture water/methanol (30/70). Yield: (0.04g, 27%). Oil. ^{1}H-NMR (300 MHz, $CDCl_3$) δ: 8.12 (d, 1H, Ar); 7.84 – 7.78 (m, 2H, Ar); 7.62 (d, 1H, Ar); 7.47 – 7.40 (m, 4H, Ar); 7.23 – 7.18 (m, 2H, 7-H, Ar); 6.91 – 6.87 (d, 2H, 6-H, Ar); 6.69 (dd, 1H, 7-H); 6.66 (s, 1H, Ar); 5.78 (s, 2H, O-CH_2); 5.21 (s, 2H, N1-CH_2); 3.99 (bs, 1H, NH); 3.02 (t, 2H, NH-$\underline{CH_2}$); 2.36 – 2.30 (m, 6H, CH_2-N, $N(CH_2)_2$); 1.86 – 1.80 (q, 2H, $CH_2\underline{CH_2}CH_2$); 1.65 – 1.57 (m, 4H, $N(CH_2)_2\underline{(CH_2)_2}$); 1.49 (m, 2H, $N(CH_2)_2(CH_2)_2)\underline{CH_2}$). ^{13}C-NMR (75 MHz, $CDCl_3$) δ: 156.0 (C-3); 143.4 (C-5); 137.3 (C-7a); 119.9 (C-6); 114.6 (C-3a); 109.9 (C-7); 98.8 (C-4); 69.6 (O-CH_2); 58.7 (NH-CH_2); 55.0 (2C, $N(CH_2)_2$); 51.8 (N1-CH_2); 45.1 (CH_2-N); 26.5 (2C, $N(CH_2)_2(\underline{CH_2})_2$); 26.0 ($CH_2\underline{CH_2}CH_2$); 24.8 ($N(CH_2)_2(CH_2)_2)\underline{CH_2}$); 138.5 (Ar); 134.2 (Ar); 133.1 (Ar); 133.0 (Ar); 132.3 (Ar); 131.9 (Ar); 130.9 (Ar); 129.5 (Ar); 129.4 (Ar); 129.0 (Ar); 127.7 (Ar); 126.9 (Ar); 126.8 (Ar); 126.3 (Ar); 125.7 (Ar); 124.6 (Ar). HPLC-MS (ES$^+$):CH_3CN/H_2O 15:85–95:5, gt: 5.00 min, rt: 3.88 min, $[M+H]^+$ =573.

1.5. 1-(3,4-dichlobenzyl)-3-(2-naphthylmethoxy)-5-(3-piperidinopropylamino)indazole **(4).** From **31** (0.13 g, 0.29 mmol), 3-piperidinopropyl chloride hydrochloride (0.98 mg, 0.5 mmol) and K_2CO_3 (0.17 g, 1.2 mmol) in CH_3CN (10 mL). Reaction time: 8 h. The product was purified by flash chromatography using as eluent the mixture water/methanol (30/70). Yield: (0.04g, 26%). Oil. ^{1}H-NMR (300 MHz, $CDCl_3$) δ: 7.97 (s, 1H, Ar); 7.88 – 7.83 (m, 3H, Ar); 7.64 (dd, 1H, Ar); 7.52 – 7.46 (m, 2H, Ar); 7.25 – 7.17 (m, 2H, 7-H, Ar); 6.96 (d, 1H, Ar); 6.86 (dd, 1H, 6-H); 6.79 – 6.73 (m, 2H, 4-H, Ar); 5.57 (s, 2H, O-CH_2); 5.25 (s, 2H, N1-CH_2); 3.79 (bs, 1H, NH); 3.16 (t, 2H, NH-$\underline{CH_2}$); 2.46 – 2.38 (m, 6H, CH_2-N, $N(CH_2)_2$); 1.81 (q, 2H, $CH_2\underline{CH_2}CH_2$); 1.62 – 1.56 (m, 4H, $N(CH_2)_2\underline{(CH_2)_2}$; 1.45 (m, 2H, $N(CH_2)_2(CH_2)_2)\underline{CH_2}$). ^{13}C-NMR (75 MHz, $CDCl_3$) δ: 155.5 (C-3); 143.1 (C-5); 137.0 (C-7a); 119.6 (C-6); 114.2 (C-3a); 109.6 (C-7); 98.4 (C-4); 70.9 (O-CH_2); 58.4 (NH-CH_2); 54.8 (2C, $N(CH_2)_2$); 51.4 (N1-CH_2); 44.8 (CH_2-N); 26.2 (2C, $N(CH_2)_2(\underline{CH_2})_2$); 25.8 ($CH_2\underline{CH_2}CH_2$); 24.6 ($N(CH_2)_2(CH_2)_2)\underline{CH_2}$); 138.2 (Ar); 134.8 (Ar); 133.4 (Ar); 133.2 (Ar); 132.6 (Ar); 131.5 (Ar); 130.6 (Ar); 129.0 (Ar); 128.3 (Ar); 128.1 (Ar); 127.8 (Ar); 127.2 (Ar); 126.5(Ar); 126.2 (Ar); 126.1 (Ar); 126.0 (Ar). HPLC-MS (ES$^+$): CH_3CN/H_2O 15:85–95:5, gt: 5.00 min, rt: 3.86 min, $[M+H]^+$ =573.

1.6. 3-(2,3-dichlorobenzyloxy)-1-(1-naphthylmethyl)-5-(3-piperidinopropylamino)indazole (**5**). From **30** (0.15 g, 0.33 mmol), 3-piperidinopropyl chloride hydrochloride (0.16 g, 0.83 mmol) and K_2CO_3 (0.28 g, 2.00 mmol) in butanone (6 mL). Reaction time: 16 h. The product was purified by flash chromatography using as eluent mixture CH_2Cl_2 / methanol (40/60). Yield: (0.03g, 17%). Oil. ^{1}H-NMR (400 MHz, acetone-d_6) δ: 8.33 (m, 2H, Ar); 7.91 – 7.88 (m, 2H, Ar); (7.67 – 7.55) (m, 4H, Ar); 7.40 – 7.35 (m, 2H, Ar); 7.23 – 7.18 (m, 2H, 7-H, Ar); 6.83 (d, 1H, 6-H); 6.66 (s, 1H, 4-H); 5.85 (s, 2H, OCH_2); 5.55 (s, 2H, N1-CH_2); 3.16 (m, 2H, NH-$\underline{CH_2}$); 2.46 – 2.41 (m, 6H, $\underline{CH_2}$-N, $N\underline{(CH_2)_2}$); 1.83 – 1.78 (m, 2H, $CH_2\underline{CH_2}CH_2$); 1.62 – 1.56 (d, 4H, $N(CH_2)_2\underline{(CH_2)_2}$); 1.46 (s, 2H, ($N(CH_2)_2(CH_2)_2)\underline{CH_2}$). ^{13}C-NMR (100 MHz, acetone-d_6) δ: 154.8 (C-3); 144.4 (C-5); 138.8 (C-7); 120.1 (C-6); 114.2 (C-3a); 111.1 (C-7); 97.2 (C-4); 68.5 (OCH_2); 58.1 (NH-CH_2); 55.2 (2C, $N(CH_2)_2$); 51.3 (N1-CH_2); 44.1 (CH_2-N); 26.6 (2C, $N(CH_2)_2\underline{(CH_2)_2}$); 26.5 ($CH_2\underline{CH_2}CH_2$); 25.1 ($N(CH_2)_2(CH_2)_2)\underline{CH_2}$); 137.7 (Ar); 134.8 (Ar); 134.7 (Ar); 133.3 (Ar); 132.2 (Ar); 131.6 (Ar); 130.6 (Ar);

129.4 (Ar); 128.9 (Ar); 128.7 (Ar); 127.0 (Ar); 126.6 (Ar); 126.4 (Ar); 126.2 (Ar); 124.6 (Ar). HPLC-MS (ES^+): CH_3CN/H_2O 15:85–95:15, gt: 10.00 min, rt: 7.20 min, $[M+H]^+$ = 575.

1.7. 1-(2-naphthylmethyl)-3-(2-naphthylmethoxy)-5-(3-piperidinopropylamino)indazole (**6**). From **28** (0.05 g, 0.11 mmol), 3-piperidinopropyl chloride hydrochloride (0.03 mg, 0.15 mmol) and K_2CO_3 (0.14 g, 1.0 mmol) in CH_3CN (8mL). Reaction time: 3 h. The dried crude was solved in 5 mL of CH_2Cl_2 and filtered to eliminate the inorganic salts. The final product was purified by flash chromatography using as eluent the mixture water/methanol 20 / 80. Yield: (0.01g, 9%). Oil. ^{1}H-NMR (400MHz, DMSO-d_6) δ: 8.03 (s, 1H, Ar); 7.94 - 7.80 (m, 5H, Ar); 7.72 - 7.63 (m, 4H, Ar); 7.54 – 7.44 (m, 4H, Ar); 7.37 (d, 1H, Ar); 7.26 (d, 1H, 7-H); 6.84 (dd, 1H, 6-H); 6.52 (d, 1H, 4-H); 5.54 (s, 2H, O-CH_2); 5.51 (s, 2H, N1-CH_2); 3.34 (bs, 2H, NH-$\underline{CH_2}$); 3.06 - 2.87 (m, 6H, CH_2-N, $N(CH_2)_2$); 1.81 (bs, 2H, $CH_2\underline{CH_2}CH_2$); 1.62 (bs, 4H, $N(CH_2)_2\underline{(CH_2)_2}$); 1.45 (bs, 2H, $N(CH_2)_2(CH_2)_2\underline{CH_2}$).^{13}C-NMR (100 MHz, acetone-d_6) δ: 155.4 (C-3); 143.4 (C-5); 137.9 (C-7a); 120.0 (C-6); 112.1 (C-3a); 111.2 (C-7); 98.2 (C-4); 71.1 (O-CH_2); 56.2 (NH-CH_2); 53.6 (2C, $N(CH_2)_2$); 53.0 (N1-CH_2); 42.5 (CH_2-N); 23.9 ($CH_2\underline{CH_2}CH_2$); 23.4 (2C, $N(CH_2)_2\underline{(CH_2)_2}$); 22.4 ($N(CH_2)_2(CH_2)_2\underline{CH_2}$); 136.8 (Ar); 136.2 (Ar); 134.3 (Ar); 133.7 (Ar); 128.9 (Ar); 128.9 (Ar); 128.8 (Ar); 128.6 (Ar); 128.5 (Ar); 128.4 (Ar); 127.9 (Ar); 127.1 (Ar); 127.0 (Ar); 127.0 (Ar); 126.9 (Ar); 126.8 (Ar); 126.7 (Ar); 126.7 (Ar); 126.6 (Ar); 126.4 (Ar). CH_3CN/H_2O 15:85–95:5, gt: 10.00 min, rt: 7.44 min, $[M+H]^+$ = 555.

***1.8. General procedure for the synthesis of derivatives* 7-12**. To a solution of the appropriate 5-aminoindazole derivative in pyridine, was added, the corresponding acyl chloride at room temperature and the mixture was stirred at room temperature. Amounts of reagents, time reaction, conditions, specific procedures and purification methods are specified in each case.

***1.9. 1-benzyl-3-benzyloxy-5-(2,3-dichlorobenzamido)indazole* (7).** From **27** (10.0 mg, 0.03 mmol), 2,3-dichlorobenzoyl chloride (10.5 mg, 0.05 mmol) and pyridine (2 mL). Reaction time: 60 h. At the end of reaction, the solution was poured on water (75 mL) and acetic acid (5 mL). The suspension was extracted with methylene chloride (3 x 25 mL). The organic phase was dried with Mg_2SO_4 and filtered. The solvent was eliminated at vacuum. The residue obtained was solved in CH_2Cl_2 (5 mL) and, after the addition of hexane (40 mL), the suspension obtained was filtered and dried. Yield: (10 mg, 66%). ^{1}H-NMR (300 MHz, $CDCl_3$) δ: 7.98 (d, 1H, Ar); 7.71 (s, 1H, Ar); 7.57 - 7.13 (m, 14H, 4-H, 6-H, 7-H, Ar); 5.44 (s, 2H, O-CH_2); 5.41 (s, 2H, N1-CH_2); ^{13}C-NMR (75 MHz, $CDCl_3$) δ: 164.3 (CO); 156.0 (C-3); 138.0 (C-7a); 132.3 (C-5); 122.5 (C-6); 113.2 (C-3a); 112.3 (C-7); 109.3 (C-4); 70.8 (O-CH_2); 52.7 (N1-CH_2); 137.6 (Ar); 137.3 (Ar); 137.0 (Ar); 134.2 (Ar); 132.3 (Ar); 129.4 (Ar); 129.3 (Ar); 128.8 (2C, Ar); 128.6 (2C, Ar); 128.2 (2C, Ar); 128.1 (Ar); 128.0 (Ar); 128.7 (Ar); 127.1 (2C, Ar). HPLC-MS (ES^+): CH_3CN/H_2O 15:85–95:5, gt: 5.00 min, rt: 6.11 min, $[M+H]^+$ = 504.

1.10. 1-benzyl-3-(1-naphthylmethoxy)-5-((N,N-diethylcarbamoyl)amino)indazole (**8**). From **32** (50 mg, 0.13 mmol), N,N-diethylcarbamoyl chloride (0.05 mL, 0.39 mmol) and pyridine (5 mL). Reaction time: 4 h. The solution was poured over 50 mL of water and the suspension obtained extracted with CH_2Cl_2. The

organic layer was evaporated to dryness and the residue was purified by flash chromatography using as eluent a mixture CH_2Cl_2 / MeOH (99:1). Yield: (21 mg, 34%). Oil. ^{1}H-NMR (400 MHz, $CDCl_3$) δ: 8.16 (m, 1H, Ar); 7.90 – 7.85 (m, 2H, Ar); 7.69 (d, 1H, 4-H); 7.53 – 7.45 (m, 4H, Ar); 7.40 (d, 1H, 6-H); 7.31 – 7.25 (m, 3H, Ar); 7.18 (d, 2H, Ar); 7.10 (d, 1H, 7-H); 6.22 (s, 1H, NH); 5.86 (s, 2H, OCH_2) 5.41 (s, 2H, $N1CH_2$); 3.32 (q, 4H, $N(CH_2)_2$); 1.17 (t, 6H, $N(CH_2)_2\underline{(CH_3)_2}$). ^{13}C-NMR (100 MHz, $CDCl_3$) δ: 155.7 (C-3); 155.3 (C-O); 137.5 (C-7a); 131.8 (C-5); 123.9 (C-6); 113.1 (C-3a); 111.6 (C-7); 109.1 (C-4); 69.1 (O-CH_2); 52.5 (N1-CH_2); 41.6 (2C, $N(CH_2)_2$); 13.9 (2C, $N(CH_2)_2\underline{(CH_3)_2}$); 139.2 (Ar); 133.7 (Ar); 132.6 (Ar); 131.4 (Ar); 129,0 (Ar); 128.6 (2C, Ar); 128.5 (Ar); 127.5(Ar); 127.1 (Ar); 127.0 (2C, Ar); 126.4 (Ar); 125.8 (Ar); 125.3 (Ar); 124.1 (Ar). HPLC-MS (ES$^+$): CH_3CN/H_2O 15:85–95:5, gt: 10.00 min, rt: 10.73 min, $[M+H]^+$ = 479.

***1.11. 1-(4-chlorobenzyl)-3-(1-naphthylmethoxy)-5-((N-methyl,N-phenylcarbamoyl)amino)indazole*)** (**9**). From **33** (50 mg, 0.12 mmol), N-methyl-N-phenylcarbamoyl chloride (26 mg, 0.15 mmol) and pyridine (3 mL). Reaction time: 46h. The organic layer was evaporated to dryness and the residue was purified by flash chromatography using as eluent a mixture CH_2Cl_2 / MeOH (99:1). Yield: (35 mg, 53%). Oil. ^{1}H-NMR (400 MHz, $CDCl_3$) δ: 8.12 (m, 1H, Ar); 7.90 – 7.84 (m, 2H, Ar); 7.66 (d, 1H, 4-H); 7.52 – 7.30 (m, 11H, 6-H, Ar); 7.09 – 7.05 (m, 3H, 7-H, Ar); 6.97 (d, 1H, Ar); 6.17 (s, 1H, NH); 5.82 (s, 2H, OCH_2); 5.32 (s, 2H, $N1CH_2$); 3.32 (s, 3H, CH_3). ^{13}C-NMR (100 MHz, $CDCl_3$) δ: 156.0 (C-3); 155.2 (C-O); 136.0 (C-7a); 131.9 (C-5); 123.4 (C-6); 113.3 (C-3a); 111.2 (C-7); 109.0 (C-4); 69.1 (O-CH_2); 52.0 (N1-CH_2); 37.4 (CH_3); 143.1 (Ar); 139.2 (Ar); 133.8 (Ar); 132.6 (Ar); 131.3 (Ar); 131.1 (Ar); 130.5 (2C, Ar); 129.6 (Ar); 129.2 (Ar); 128.9 (2C, Ar); 128.7 (Ar); 128.5 (2C, Ar); 128.0 (Ar); 127.6 (2C, Ar); 127.3 (Ar); 126.6 (Ar); 125.4 (Ar); 124.1 (Ar). HPLC-MS (ES$^+$): CH_3CN/H_2O 15:85–95: 5, gt: 10.00 min, rt: 11.55 min, $[M+H]^+$ = 547.

1.12. 1-(3,4-dichlorobenzyl)-3-(1-naphthylmethoxy)-5-((N,N-diphenylcarbamoyl)amino)indazole (**10**). From **34** (48 mg, 0.11 mmol), N,N-diphenyllcarbamyl chloride (35 mg, 0.15 mmol) and pyridine (3 mL). Reaction time: 24 h. The organic layer was evaporated to dryness and the residue was purified by flash chromatography using as eluent a mixture CH_2Cl_2 / MeOH (99:1). Yield: (39 mg, 55%). Oil. ^{1}H-NMR (400 MHz, $CDCl_3$) δ: 8.16 (m, 1H, Ar); 7.90 – 7.85 (m, 2H, Ar); 7.66 (d, 1H, 4-H); 7.53 – 7.43 (m, 5H, 6-H, Ar); 7.39 – 7.22 (m, 14H, Ar); 7.08 (d, 1H, 7-H); 6.97 (d, 1H, Ar); 6.41 (s, 1H, NH); 5.83 (s, 2H, OCH_2); 5.33 (s, 2H, $N1CH_2$); ^{13}C-NMR (100 MHz, $CDCl_3$) δ: 156.2 (C-3); 154.2 (C-O); 137.8 (C-7a); 131.8 (C-5); 123.1 (C-6); 113.4 (C-3a); 111.2 (C-7); 108.9 (C-4); 69.2 (O-CH_2); 51.5 (N1-CH_2); 142.5 (Ar); 139.2 (Ar); 133.8 (Ar); 132.8 (Ar); 132.4 (Ar); 131.9 (Ar); 131.4 (Ar); 131.1 (Ar); 130.7 (Ar); 129.7 (4C, Ar); 129.2 (Ar); 129.1 (Ar); 128.7 (Ar); 127.6 (3C, Ar); 127.3 (Ar); 126.8 (2C, Ar); 126.6 (Ar); 126.5 (Ar); 126.0 (Ar); 125.4 (Ar); 124.0 (Ar). HPLC-MS (ES$^+$): CH_3CN/H_2O 15:85–95:5, gt: 10.00 min, rt: 12.81 min, $[M+H]^+$ = 643.

1.13. 3-(benzyloxy-1-(2,3-dichlorobenzyl)-5-(((4-methylpiperazino)carbonyl)amino)indazole (**11**). From **29** (50 mg, 0.12 mmol), (4-methylpiperazyne)carbonyl chloride hydrocloride (37 mg, 0.18 mmol) and pyridine (5 mL). After 20h, (4-methylpiperazyne)carbonyl chloride hydrocloride (37 mg, 0.18 mmol) was added. Reaction time: 44 h. The organic layer was evaporated to dryness and the residue was purified by

flash chromatography using as eluent a mixture CH_2Cl_2 / MeOH (99:1). Yield: (35 mg, 58%). Oil. ^{1}H-NMR (400 MHz, $CDCl_3$) δ: 7.66 (d, 1H, 4-H); 7.49 – 7.47 (m, 2H, Ar); 7.38 – 7.29 (m, 5H, 6-H, Ar); 7.08 (d, 1H, 7-H); 6.98 (t, 1H, Ar); 6.48 (d, 2H, Ar); 5.47 (s, 2H, OCH_2) 5.39 (s, 2H, $N1CH_2$); 3.69 (s, 1H, NH); 3.52 (t, 4H, $N(CH_2)_2$); 2.46 (t, 4H, $N(CH_2)_2\underline{(CH_2)_2}$); 2.33 (s, 3H, CH_3). ^{13}C-NMR (100 MHz, $CDCl_3$) δ: 156.1 (C-3); 155.8 (C-O); 137.0 (C-7a); 131.5 (C-5); 124.1 (C-6); 113.3 (C-3a); 112.1 (C-7); 109.0 (C-4); 70.7 (O-CH_2); 54.7 (2C, $N(CH_2)_2$); 50.4 (N1-CH_2); 46.0 (CH_3); 43.9 (2C, $N(CH_2)_2\underline{(CH_2)_2}$); 139.5 (Ar); 137.7 (Ar); 133.2 (Ar); 130.4 (Ar); 129.4 (Ar); 128.5 (Ar); 128.1 (2C, Ar); 128.0 (2C, Ar); 127.6 (Ar); 126.3 (Ar). HPLC-MS (ES^+): CH_3CN/H_2O 15:85–95:5, gt: 10.00 min, rt: 7.10 min, $[M+H]^+$ = 524.

1.14. 3-(2,3-dichlorobenzyloxy)-1-(1-naphthylmethyl)-5((1-pyrrolidinyl-carbonyl)amino)indazole (**12**). From **30** (50 mg, 0.11 mmol), 1-pyrrolidinylcarbonyl chloride (0.03 mL, 0.15 mmol) and pyridine (3 mL). Reaction time: 20 h. The crude was poured over 50 ml of H_2O and was extracted with CH_2Cl_2 (3x15 mL). The organic layer was evaporated to dryness and the residue was purified by flash chromatography using as eluent a mixture CH_2Cl_2 / MeOH (99:1). Yield: (27 mg, 45%). Oil. ^{1}H-NMR (400 MHz, $CDCl_3$) δ: 8.16 (m, 1H, Ar); 7.90 - 7.85 (m, 1H, Ar); 7.78 – 7.76 (m, 2H, Ar); 7.67 – 7.49 (m, 3H, 4-H, Ar); 7.42 (d, 1H, 6-H); 7.33 (t, 1H, Ar); 7.27 (d, 1H, Ar); 7.20 (t, 1H, Ar); 7.03 (d, 1H, 7-H); 6.96 (d, 1H, Ar); 6.15 (s, 1h, NH); 5.84 (s, 2H, OCH_2) 5.55 (s, 2H, $N1CH_2$); 3.44 (m, 4H, $N(CH_2)_2$); 1.95 (m, 4H, $N(CH_2)_2\underline{(CH_2)_2}$). ^{13}C-NMR (100 MHz, $CDCl_3$) δ: 155.2 (C-3); 154.7 (C-O); 137.5 (C-7a); 131.6 (C-5); 123.4 (C-6); 113.1 (C-3a); 111.1 (C-7); 109.5 (C-4); 68.1 (O-CH_2); 51.1 (N1-CH_2); 45.9 (2C, $N(CH_2)_2$); 25.8 (2C, $N(CH_2)_2\underline{(CH_2)_2}$); 139.4 (Ar); 133.8 (Ar); 133.1 (Ar); 132.8 (Ar); 131.2 (Ar); 131.1(Ar); 129.6 (Ar); 128.9 (Ar); 128.4 (Ar); 127.4 (Ar); 127.2 (Ar); 126.5 (Ar); 126.0 (Ar); 125.5 (Ar); 125.3 (Ar); 123.3 (Ar). HPLC-MS (ES^+): CH_3CN/H_2O 15:85–95:5, gt 10.00 min, rt: 11.71 min, $[M+H]^+$ = 545.

1.15. General Procedure *for the synthesis of N-1 substituted 3-indazolol derivatives* **17**-**20.** To a solution of 5-nitro-1*H*-3-indazolol (**13**) in a 1N solution of NaOH in water, the corresponding halide was added. The reaction mixture was heated to 70 ºC and stirred until complete elimination of the indazolol derivative. The suspension obtained was cooled to room temperature and the resulting precipitate was separated by vacuum filtration. Amounts of reagents, time reaction, conditions, specific procedures and purification methods are specified in each case.

1.16. 1-(4-chlorobenzyl)-5-nitro-3-indazolol (**17***)*. From **13** (0.88 g, 4.93 mmol), NaOH aq. 1N (5 mL) and 4-chlorobenzyl bromide (1.13 g; 5.50 mmol). Time of reaction: 20 h. At the end of the reaction, the red suspension was neutralized with AcOH and filtered, washed with H_2O and CH_2Cl_2. Yield: (1.41 g, 94%). Mp 315 °C. ^{1}H-NMR (300 MHz, DMSO-d_6) δ: 11.55 (1H, OH); 8.66 (d, 1H, 4-H); 8.18 (dd, 1H, 6-H); 7.78 (d, 1H, 7-H); 7.38 (d, 2H, Ar); 7.25 (d, 2H, Ar); 5.48 (s, 2H, N1-CH_2). ^{13}C-NMR (75 MHz, DMSO-d_6) δ: 156.5 (C-3); 142.0 (C-7a); 137.3 (C-5); 120.5 (C-6); 120.2 (C-4); 119.0 (C-3a); 107.3 (C-7); 50.1 (N1-CH_2); 136.2 (Ar); 131.6 (Ar); 129.2 (2C, Ar); 128.2 (2C, Ar). HPLC-MS (ES^+): CH_3CN/H_2O 15:85–95:5, gt: 5.00 min; rt: 4.77, $[M+H]^+$ = 304.

1.17. 1-(2,3-dichlorobenzyl)-5-nitro-3-indazolol (**18**)**.** From **13** (1.00 g, 5.60 mmol), NaOH aq. 1N (8.5 mL) and 2,3-dichlorobenzyl bromide (1.70 g, 7.30 mmol). After 3 h. was added more 2,3-dichlorobenzyl chloride (0.16 g, 0.70 mmol). Reaction time: 20 h. At the end of the reaction, the red suspension was filtered, washed with AcOH aq, H_2O and hexane. Yield: (1.534 g, 81%). Mp 273 °C. ^{1}H-NMR (400 MHz, DMSO-d_6) δ: 11.70 (s, 1H, OH); 8.68 (d, *J* = 2.2 Hz, 1H, 4-H); 8.20 (dd, 1H, 6-H); 7.77 (d, 1H, 7-H); 7.60 (d, 1H, Ar); 7.32 (t, 1H, Ar); 6.96 (d, 1H, Ar); 5.60 (s, 2H, CH_2-N). ^{13}C-NMR (100 MHz, DMSO-d_6) δ: 157.0 (C-3); 142.7 (C-7a); 140.2 (C-5); 122.1 (C-6); 118.6 (C-4); 111.8 (C-3a); 110.1 (C-7); 49.9 (N1-CH_2); 137.0 (Ar); 132.0 (Ar); 130.2 (Ar); 129.9 (Ar); 128.3 (Ar); 128.3 (Ar). HPLC-MS (ES$^+$): CH_3CN/H_2O 40:60 – 95:5, gt: 10.00 min; rt: 5.58, $[M+H]^+$ = 338.

1.18. 1-(3,4-dichlorobenzyl)-5-nitro-3-indazolol (**19**)**.** From **13**, (0.21 g, 1.19 mmol), NaOH aq. 1N (5 mL) and 3,4-dichlorobenzyl chloride (0.32 g, 1.40 mmol). After 3 h. was added more 3,4-dichlorobenzyl chloride (0.16 g, 0.70 mmol). At the end of the reaction, the red suspension was poured on HCl 5% (30 mL). The resulting yellow suspension was filtered, washed with water and air-dried. Reaction time: 24 h. Yield: (0.28 g, 68%). Mp 257 °C. ^{1}H-NMR (300 MHz, DMSO-d_6) δ: 11.58 (s, 1H, OH); 8.68 (d, 1H, 4-H); 8.20 (dd, 1H, 6-H); 7.82 (d, *J* = 9.3 Hz, 1H, Ar); 7.58 (m, 2H, 7-H, Ar); 7.20 (dd, *J* = 2.0 Hz, 1H, Ar); 5.51 (s, 2H, N1-CH_2). ^{13}C-NMR (75 MHz, DMSO-d_6) δ: 157.2 (C-3), 142.7 (C-7a), 140.2 (C-5), 122.5 (C-6), 119.0 (C-4), 112.2 (C-3a), 110.4 (C-7); 51.4 (N1-CH_2); 138.5 (Ar); 131.4 (Ar); 131.2 (Ar); 130.7 (Ar); 130.0 (Ar); 128.2 (Ar); HPLC-MS (ES$^+$): CH_3CN / H_2O 10/90 – 85/15, gt: 6.00 min; rt: 5.06; $[M+H]^+$ = 338.

1.19. 1-(1-naphthylmethyl)-5-nitro-3-indazolol (**20**)**.** From **13** (1.00 g, 5.60 mmol), NaOH aq. 1N (6 mL) and 1-naphthylmethyl chloride (0.98 g, 5.60 mmol). Time of reaction: 20 h. At the end of the reaction, the red suspension was neutralized with AcOH and filtered, washed with H_2O and hexane. Yield: (0.52 g, 29%). Mp 261 °C. ^{1}H-NMR (400 MHz, DMSO-d_6) δ: 11.52 (s, 1H, OH); 8.68 (d, 1H, 4-H); 8.21 (m, 2H, 6-H, Ar); 7.97 (m, 1H, Ar); 7.79 (d, 2H, 7-H, Ar); 7.46 (t, 1H, Ar); 7.20 (d, 1H, Ar); 5.97 (s, 2H, N1-CH_2). ^{13}C-NMR (100 MHz, DMSO-d_6) δ: 156.5 (C-3); 142.3 (C-7a); 140.1 (C-5); 122.0 (C-6); 118.6 (C-4); 111.8 (C-3a); 110.2 (C-7); 49.9 (N1-CH_2); 133.4 (Ar); 132.5 (Ar); 130.8 (Ar); 128.6 (Ar); 128.3 (Ar); 126.5 (Ar); 126.0 (C, Ar); 125.5 (Ar); 123.5 (Ar). HPLC-MS (ES$^+$): CH_3CN/H_2O 40:60–95:5, gt: 10.00 min; rt: 4.93, $[M+H]^+$ = 320.

1.20. General procedure for the synthesis of 5-nitroindazole ethers 1-substituted **21-26.** To a suspension of the appropriate 1-substituted derivative of 5-nitro-3-indazolol in 2-butanone, K_2CO_3, and KI, the corresponding bromide or chloride was added and stirred at room temperature until complete elimination of N1-H indazole ether. Then, the reaction mixture was cooled and filtered to remove existing inorganic salts. The solvent was evaporated under reduced pressure and purified. The resulting crude product was processed in each case. Amounts of reagents, time reaction, conditions, specific procedures and purification methods are specified in each case.

1.21. 3-(benzyloxy)-1-(2,3-dichlorobenzyl)-5-nitroindazole (**21**). From **18** (1.00 g, 2.96 mmol), benzyl bromide (0.42 mL 3.55 mmol) and K_2CO_3 (0.90 g, 6.50 mmol) in butanone (60 mL). Reaction time: 20 h.

The dried crude was solved in 10 mL of CH_2Cl_2. After addition of 50 mL of hexane, the resulting suspension was filtered to obtain a yellow solid. The final product was purified by recrystallisation from isopropanol. Yield: (0.79 g, 62%). Mp 142.0 °C. ^{1}H-NMR (400 MHz, $CDCl_3$) δ: 8.70 (d, 1H, 4-H); 8.23 (dd, 1H, 6-H); 7.52 – 7.49 (m, 2H, Ar); 7.43 – 7.36 (m, 4H, 7-H, Ar); 7.25 (d, 1H, Ar); 7.09 (t, 1H, Ar); 6.70 (dd, 1H, Ar); 5.55 (s, 2H, CH_2-O); 5.44 (s, 2H, CH_2-N). ^{13}C-NMR (100 MHz,$CDCl_3$) δ: 158.3 (C-3); 143.6 (C-7a); 141.8 (C-5); 123.5 (C-6); 120.1 (C-4); 113.1 (C-3a); 109.3 (C-7); 71.7 (CH_2-O); 51.1 (CH_2-N); 136.6 (Ar); 136.5 (Ar); 133.9 (Ar); 131.3 (Ar); 130.4 (Ar); 129.0 (Ar); 128.8 (2C, Ar); 128.7 (Ar); 128.0 (2C, Ar); 127.0 (Ar). HPLC-MS (ES$^+$):CH_3CN/H_2O 40:60 – 95:5, gt: 10.00 min, rt: 10.88 min, $[M+H]^+$ = 428.

1.22. 3-(2,3-dichlorobenzyloxy)-1-(1-naphthylmethyl)-5-nitroindazole (**22**)**.** From **20** (0.64 g, 2.03 mmol), 2,3-dichlorobenzyl bromide (0.58 g, 2.43 mmol) and K_2CO_3 (0.60 g, 4.40 mmol) in butanone (60 mL). Reaction time: 20 h. The dried crude was solved in 10 mL of CH_2Cl_2. After addition of 50 mL of hexane, the resulting suspension was filtered to obtain a yellow solid. The final product was purified by recrystallisation from isopropanol. Yield: (0.49 g, 60%). Mp 137 °C. ^{1}H-NMR (400 MHz, $CDCl_3$) δ: ^{1}H-NMR (400 MHz, $CDCl_3$) δ: 8.71 (d, 1H, 4-H); 8.15 – 8.12 (m, 2H, 6-H, Ar); 7.90 – 7.82 (m, 2H, Ar); 7.54 – 7.33 (m, 5H, 7-H, Ar); 7.25 – 7.11 (m, 3H, Ar); 5.90 (s, 2H, O-CH_2); 5.60 (s, 2H, N-CH_2). ^{13}C-NMR (100 MHz, $CDCl_3$) δ: 157.1 (C-3); 143.2 (C-7a); 141.3 (C-5); 122.8 (C-6); 118.6 (C-4); 112.6 (C-3a); 109.4 (C-7); 68.6 (O-CH_2); 51.8 (N1-CH_2); 136.5 (Ar); 134.0 (Ar); 133.4 (Ar); 131.4 (Ar); 131.1 (Ar); 130.2 (Ar); 129.2 (Ar); 129.0 (Ar); 127.5 (2C, Ar); 127.4 (Ar); 126.9 (Ar); 129.3 (Ar); 125.8 (Ar); 125.4 (Ar); 123.1 (Ar). HPLC-MS (ES$^+$): CH_3CN/H_2O 40:60–95:5, gt: 10.00 min, rt: 10.92 min, $[M+H]^+$ = 479.

1.23. 1-(3,4-dichlorobenzyl)-3-(2-naphthylmethoxy)-5-nitroindazole (**23**)**.** From **19** (0.70 g, 2.1 mmol), 2-naphthylmethyl bromide (0.53 g, 2.4 mmol) and K_2CO_3 (0.60 g, 4.3 mmol) in butanone (60 mL). Reaction time: 24 h. The final product was purified by flash chromatography using as eluent a mixture hexane: methylene chloride (4:1). Yield: (0.56 g, 55%). Mp 147 °C. ^{1}H-NMR (300 MHz, $CDCl_3$) δ: 8.70 (d, 1H, 4-H); 8.20 (dd, 1H, 6-H); 7.94 - 7.81 (m, 4H, Ar); 7.61 – 7.46 (m, 3H, Ar); 7.30 – 7.16 (m, 3H, 7-H, Ar); 6.92 (dd 1H, Ar); 5.59 (s, 2H, O-CH_2); 5.33 (s, 2H, N1-CH_2). ^{13}C-RMN: (75 MHz, $CDCl_3$) δ: 157.9 (C-3), 142.9 (C-7a), 141.5 (C-5), 123.1 (C-6); 118.9 (C-4), 113.0 (C-3a), 108.7 (C-7); 71.5 (O-CH_2); 51.8 (N1-CH_2); 136.3 (Ar); 133.7 (Ar); 133.3 (Ar); 133.1 (Ar); 132.4 (Ar); 131.0 (Ar); 129.3 (Ar); 128.5 (Ar); 128.2 (Ar); 127.9 (Ar); 127.5 (Ar); 126.6 (Ar); 126.5 (2C, Ar); 125.9 (Ar). HPLC-MS (ES$^+$): CH_3CN/H_2O 15:85–95:5 gt: 5 min, rt: 7.11 min, $[M+H]^+$ =478.

1.24. 1-benzyl-3-(1-naphthylmethoxy)-5-nitroindazole (**24**)**.** From **16** (1.00 g, 3.74 mmol), 1-naphthylmethyl chloride (0.79 g, 4.40 mmol) and K_2CO_3 (1.16 g, 8.4 mmol) in butanone (60 mL). Reaction time: 24 h. The dried crude was solved in 10 mL of CH_2Cl_2. After addition of 50 mL of hexane, the resulting suspension was filtered to obtain a yellow solid. The final product was purified by recrystallisation from isopropanol. Yield: (0.72 g, 52%). Mp 123.1 °C. ^{1}H-NMR (400 MHz, $CDCl_3$) δ: 8.60 (d, 1H, 4-H); 8.15 (d,

2H, 6-H, Ar); 7.89 (t, 2H, Ar); 7.69 (d, 1H, Ar); 7.56 (m, 3H, Ar); 7.32 (m, 3H, Ar); 7.22 (m, 2H, Ar); 7.17 (d, 1H, 7-H); 5.89 (s, 2H, O-CH_2); 5.43 (s, 2H, N1-CH_2). ^{13}C-NMR (100 MHz,$CDCl_3$) δ: 157.8 (C-3); 142.9 (C-7a); 141.2 (C-5); 122.8 (C-6); 118.8 (C-4); 112.8 (C-3a); 109.0 (C-7); 69.7 (O-CH_2); 53.1 (N1-CH_2); 136.2 (Ar); 133.9 (Ar); 131.9 (Ar); 131.8 (Ar); 129.5 (Ar); 129.0 (2C, Ar); 128.8 (Ar); 128.2 (Ar); 127.7 (Ar); 127.3 (2C, Ar); 126.8 (Ar); 126.1 (Ar); 125.4 (Ar); 123.9 (Ar). HPLC-MS (ES$^+$):CH_3CN/H_2O 40:60–95:5, gt: 10.00 min, rt: 10.45 min, $[M+H]^+$ = 410.

1.25. 1-(4-chlorobenzyl)-3-(1-naphthylmethoxy)-5-nitroindazole (**25**). From **17** (0.60 g, 1.96 mmol), 1-naphthylmethyl chloride (0.40 g, 2.25 mmol) and K_2CO_3 (0.41 g, 2.97 mmol) in butanone (40 mL). Reaction time: 24 h. The final product was obtained by recrystallisation from isopropanol. Yield: (0.41 g, 47%). Mp 160.4 °C. ^{1}H-NMR (300 MHz, $CDCl_3$) δ: 8.64 (d, 1H, 4-H); 8.21 – 8.15 (m, 2H, 6-H, Ar); 7.93 – 7.88 (m, 2H, Ar); 7.69 (d, 1H, Ar); 7.58 – 7.48 (m, 3H, Ar); 7.31 – 7.26 (m, 2H, 7-H, Ar); 7.21 – 7.14 (m, 3H, Ar); 5.90 (s, 2H, O-CH_2); 5.41 (s, 2H, N1-CH_2). ^{13}C-NMR (75 MHz, $CDCl_3$) δ: 157.9 (C-3), 142.9 (C-7a), 141.3 (C-5), 123.0 (C-6); 118.9 (C-4), 112.9 (C-3a), 108.8 (C-7); 69.7 (O- CH_2); 52.4 (N1-CH_2); 134.7 (Ar); 134.2 (Ar); 133.9 (Ar); 131.9 (Ar); 131.7 (Ar); 129.6 (2C, Ar); 129.2 (Ar); 128.9 (Ar); 128.7 (2C, Ar); 127.7 (Ar); 126.8 (Ar); 126.2 (Ar); 125.3 (Ar); 123.0 (Ar). HPLC-MS (ES$^+$): CH_3CN/H_2O 80:20–95:5, gt: 5.00 min, rt: 3.40 min, $[M+H]^+$ = 444.

1.26. 1-(3,4-dichlorobenzyl)-3-(1-naphthylmethoxy)-5-nitroindazole (**26**)**.** From **19** (0.70 g, 2.1 mmol), 1-naphthylmethyl chloride (0.53 g, 2.4 mmol) and K_2CO_3 (0.60 g, 4.3 mmol) in butanone (60 mL). Reaction time: 96 h. The final product was purified by flash chromatography using as eluent a mixture hexane: methylene chloride (4:1). Yield: (0.34 g, 35%). Mp. 140 °C. ^{1}H-NMR (300 MHz, $CDCl_3$) δ: 8.65 (d, 1H, 4-H); 8.23 (d, 1H, 6-H); 8.17 (d, 1H, Ar); 7.90 (t, 2H, Ar); 7.69 (d, 1H, Ar); 7.58 – 7.47 (m, 4H, Ar); 7.39 (d, 1H, Ar); 7.34 (1H, d, 7-H); 7.20 (d,1H, Ar); 7.03(d, 1H, Ar); 5.90 (s, 2H, O-CH_2); 5.39 (s, 2H, N1-CH_2). ^{13}C-NMR (75 MHz, $CDCl_3$) δ: 158.2 (C-3), 142.7 (C-7a), 141.8 (C-5), 123.4 (C-6); 119.2 (C-4), 113.3 (C-3a), 109.0 (C-7); 71.8 (O-CH_2); 51.4 (N1-CH_2); 143.2 (Ar); 136.6 (Ar); 133.9 (Ar); 133.6 (Ar); 133.4 (Ar); 132.7 (Ar); 131.2 (Ar); 129.6 (Ar); 128.8 (Ar); 128.4 (Ar); 128.2 (Ar); 127.8 (Ar); 126.9 (Ar); 126.8 (2C, Ar); 126.2 (Ar). HPLC-MS (ES$^+$): CH_3CN/H_2O 15:85–95:5, gt: 5.00 min, rt: 7.11 min, $[M+H]^+$ = 478.

***1.27. General procedure for the synthesis of 5-aminoindazoles* 28-34.**

A mixture of 5-nitroindazole derivative and a catalytic amount of FeO(OH) in methanol or ethanol was allowed to react with monohydrated hydrazine in excess under argon atmosphere. The reaction mixture was stirred at 50 – 70 ºC until the elimination of 5-nitroindazole. Then, the suspension was filtered throughout of zelite and the solvent was evaporated under vacuum. The resulting crude product was processed in each case. Amounts of reagents, time reaction, conditions, specific procedures and purification methods are specified in each case.

1.28. 5-amino-3-(2-naphthylmethoxy)-1-(2-naphthylmethyl)indazole (**28**)**.** From **15** (0.21 g, 0.46 mmol), hydrazine monohydrated (1.0 mL, 2.62 mmol) and FeO(OH) (0.01 g, 0.1 mmol) in ethanol (25 mL). Time of reaction: 23 h. The dried crude was suspended on hexane (30 mL) and filtered to obtain a white solid. Yield: (0.20 g, 99%). Mp 149 °C. ^{1}H-NMR (300 MHz, $CDCl_3$) δ: 7.97 (s, 1H, Ar); 7.87 – 7.76 (m, 5H, Ar); 7.70 – 7.58 (m, 5H, Ar); 7.50 – 7.42 (m, 4H, Ar); 7.28 – 7.24 (m, 1H, Ar); 7.03 (d, 1H, 7-H); 6.96 (d, 1H, 4-H); 6.80 (dd, 1H, 6-H); 5.60 (s, 2H, O-CH_2); 5.50 (s, 2H, N1-CH_2); 3.49 (bs, 2H, NH_2). ^{13}C-NMR (75 MHz, $CDCl_3$) δ: 155.1 (C-3); 139.4 (C-5); 137.8 (C-7a); 119.5 (C-6); 114.0 (C-3a); 110.0 (C-7); 103.2 (C-4); 70.9 (O-CH_2); 52.9 (N1-CH_2); 135.3 (Ar); 134.9 (Ar); 133.4 (Ar); 133.2 (Ar); 132.9 (Ar); 128.5 (Ar); 128.4 (Ar); 128.3 (Ar); 128.2 (Ar); 128.1 (Ar); 128.0 (Ar); 127.8 (Ar); 127.1 (Ar); 126.2 (Ar); 126.1 (Ar); 125.9 (Ar); 125.8 (Ar); 125.3 (Ar). HPLC-MS (ES$^+$):CH_3CN/H_2O 15:85-95:5, gt: 10.00 min, tr: 8.19 min, $[M+H]^+$ = 430.

1.29. 5-amino-3-(benzyloxy)-1-(2,3-dichlorobenzyl)indazole (**29**). From **21** (0.65 g, 1.52 mmol), hydrazine monohydrated (1.9 mL, 3.32 mmol) and FeO(OH) (18 mg, 0.18 mmol) in methanol (40 mL). Reaction Time: 48 h. The dried crude was solved in 5 mL of CH_2Cl_2 and poured over 45 mL of Hexane. The white suspension obtained was filtered and dried to give the final product as white solid. Yield: (0.52 g, 86%). Mp 117.4 ºC. ^{1}H-NMR (400 MHz, $CDCl_3$) δ: 7.52 – 7.49 (m, 2H, Ar); 7.40 – 7.31 (m, 4H, Ar); 7.01 – 6.97 (m, 2H, 7-H, Ar); 6.94 (d, 1H, 4-H); 6.83 (dd, 1H, 6-H); 6.49 (dd, 1H, Ar); 5.45 (s, 2H,CH_2-O); 5.41 (s, 2H, CH_2-N); 3.53 (s, 2H, NH_2). ^{13}C-NMR (100 MHz, $CDCl_3$) δ: 155.7 (C-3); 139.7 (C-5); 138.3 (C-7); 120.1 (C-6); 114.2 (C-3a); 110.0 (C-7); 103.7 (C-4); 71.0 (CH_2-O); 50.6 (CH_2-N); 138.1 (Ar); 137.6 (Ar); 133.4 (Ar); 130.7 (Ar); 129.6 (Ar); 128.8 (Ar); 128.5 (Ar); 128.4 (Ar); 127.8 (Ar); 126.6 (Ar). CH_3CN/H_2O 40:60–95:5, gt: 10.00 min, rt: 7.71 $[M+H]^+$ = 398.

1.30. 5-amino-3-(2,3-dichlorobenzyloxy)-1-(1-naphthylmethyl)indazole (**30**)**.** From **22** (0.44 g, 0.92 mmol), hydrazine monohydrated (1.2 mL, 2.1 mmol) and FeO(OH) (11 mg, 0.11 mmol) in methanol (40 mL). Reaction Time: 72 h. The dried crude was solved in 5 mL of diethylic ether and poured over 45 mL of Hexane. The white suspension obtained was filtered and dried to give the final product as white solid. Yield: (0.36 g, 87%). Mp 127.2 ºC. ^{1}H-NMR (400 MHz, $CDCl_3$) δ: 8.18 – 8.16 (m, 1H, Ar); 7.88 – 7.85 (m, 1H, Ar); 7.77 (d, 1H, Ar); 7.55 – 7.49 (m, 3H, Ar); 7.43 (d, 1H, Ar); 7.33 (t, 1H, Ar); 7.19 (t, 1H, Ar); 7.00 – 6.96 (m, 2H, Ar, 7-H, Ar); 6.94 (d, 1H, 4-H); 6.80 (dd, 1H, 6-H); 5.81 (s, 2H,CH_2-O); 5.56 (s, 2H, N-CH_2); 3.53 (bs, 2H, NH_2). ^{13}C-NMR (100 MHz, $CDCl_3$) δ: 154.3 (C-3); 139.5 (C-5); 137.8 (C-7); 119.6 (C-6); 113.6 (C-3a); 110.2 (C-7); 102.9 (C-4); 68.2 (O-CH_2); 51.1 (N1-CH_2); 137.6 (Ar); 133.8 (Ar); 133.1 (Ar); 133.0 (Ar); 131.3 (Ar); 131.2 (Ar); 129.7 (Ar); 128.8 (Ar); 128.3 (Ar); 127.3 (Ar); 127.3 (Ar); 126.5 (Ar); 125.9 (Ar); 123.3 (Ar). CH_3CN/H_2O 40:60–95:5, gt: 10.00 min, rt: 5.05 $[M+H]^+$ = 448.

1.31. 5-amino-1-(3,4-dichlorobenzyl)-3-(2-naphthylmethoxy)indazole (**31**)**.** From **23** (0.30 g, 0.63 mmol), hydrazine monohydrated (3.0 mL, 7.88 mmol) and FeO(OH) (0.05 g, 0.50 mmol) in ethanol (30 mL). Reaction time: 72 h. The dried crude was suspended on water (25 mL) and extracted with diethylic ether (3 x 10 mL). The organic phase was dried with Mg_2SO_4 and filtered. The organic phase was evaporated at

vacuum and over the residue was added methylene chloride (5 mL) and hexane (25 mL). The resulting suspension was filtered and the solid obtained was washed with hexane and air-dried. Yield: (0.21 g, 76%). Mp 111.3 ºC. ^{1}H-NMR (300 MHz, $CDCl_3$) δ: 7.97 (s, 1H, Ar); 7.89 – 7.84 (m, 2H, Ar); 7.63 (d, 1H, Ar); 7.54 – 7.49 (m, 2H, Ar); 7.27 – 7.19 (m, 2H, Ar); 6.99 – 6.96 (m, 2H, 7-H, Ar); 6.87 (d, 1H, 4-H); 6.82 (d, 1H, 6-H); 5.59 (s, 2H, O-CH_2); 5.25 (s, 2H, N1-CH_2); 3.44 (bs, 2H, NH_2). ^{13}C-NMR (75 MHz, $CDCl_3$) δ: 155.7 (C-3); 138.3 (C-5); 137.9 (C-7a); 120.0 (C-6); 114.5 (C-3a); 110.0 (C-7); 103.6 (C-4); 71.2 (O-CH_2); 51.7 (N1-CH_2); 140.0 (Ar); 135.0 (Ar); 133.7 (Ar); 133.5 (Ar); 133.0 (Ar); 131.9 (Ar); 130.9 (Ar); 129.4 (Ar); 128.6 (Ar); 128.1 (Ar); 127.5 (Ar); 127.4 (Ar); 126.8 (Ar); 126.6 (Ar); 126.5 (Ar); 126.4 (Ar). HPLC-MS (ES$^+$): CH_3CN/H_2O 15:85–95:5, gt: 5.00 min; rt: 4.93. $[M+H]^+$ = 448.

1.32. 5-amino-1-benzyl-3-(1-naphthylmethoxy)indazole (**32**). From **24** (0.65 g, 1.58 mmol), hydrazine monohydrated (2.6 mL, 4.55 mmol) and FeO(OH) (18 mg, 0.20 mmol) in methanol (40 mL). Reaction Time: 24 h. The dried crude was solved in 5 mL of CH_2Cl_2 and poured over 45 mL of Hexane. The white suspension obtained was filtered and dried to give the final product as white solid. Yield: (0.55 g, 92%). Mp 258.6 ºC. ^{1}H-NMR (400 MHz, $CDCl_3$) δ: 8.22 (d, 1H, Ar); 7.94 – 7.88 (m, 2H, Ar); 7.71 (d, 1H, Ar); 7.58 – 7.48 (m, 3H, Ar); 7.34 – 7.21 (m, 5H, Ar); 7.03 (d, 1H, 7-H); 6.88 (d, 1H, 4-H); 6.81 (dd, 1H, 6-H); 5.89 (s, 2H, O-CH_2); 5.41 (s, 2H, N1-CH_2); 3.50 (s, 2H, NH_2). ^{13}C-NMR (100 MHz, $CDCl_3$) δ: 155.2 (C-3); 138.2 (C-5); 137.9 (C-7a); 119.7 (C-6); 113.9 (C-3a); 110.0 (C-7); 104.1 (C-4); 69.3 (O-CH_2); 52.7 (N1-CH_2); 137.8 (Ar); 133.9 (Ar); 132.9 (Ar); 132.0 (Ar); 129.1 (Ar); 128.7 (3C, Ar); 127.6 (Ar); 127.3 (Ar); 127.2 (2C, Ar); 126.5 (Ar); 126.0 (Ar); 125.4 (Ar); 124.3 (Ar). CH_3CN/H_2O 15:85–95:5, gt: 10.00 min, rt: 7.20, $[M+H]^+$ =380.

1.33. 5-amino-1-(4-chlorobenzyl)-3-(1-naphthylmethoxy)indazole (**33**). From **25** (0.25 g, 0.56 mmol), hydrazine monohydrated (1 mL, 1.75 mmol) and FeO(OH) (6 mg, 0.07 mmol) in ethanol (20 mL). Reaction Time: 48 h. The dried crude was solved in 5 mL of CH_2Cl_2 and poured over 45 mL of Hexane. The white suspension obtained was filtered and dried to give the final product as white solid. Yield: (0.20 g, 88%). Mp 156.9 ºC. ^{1}H-NMR (400 MHz, $CDCl_3$) δ: 8.22 – 8.19 (m, 1H, Ar); 7.94 – 7.89 (m, 2H, Ar); 7.70 (d, 1H, Ar); 7.57 – 7.48 (m, 3H, Ar); 7.28 (d, 2H, Ar); 7.13 (d, 2H, Ar); 7.01 (d, 1H, 7-H); 6.89 (d, 1H, 4-H); 6.82 (d, 1H, 6-H); 5.88 (s, 2H, O-CH_2); 5.36 (s, 2H, N1-CH_2); 3.50 (bs, 2H, NH_2). ^{13}C-NMR (100 MHz, $CDCl_3$) δ: 155.3 (C-3); 139.1 (C-5); 137.7 (C-7a); 119.7 (C-6); 114.1 (C-3a); 109.8 (C-7); 103.6 (C-4); 69.3 (O-CH_2); 52.0 (N1-CH_2); 136.3 (Ar); 133.9 (Ar); 132.8 (Ar); 132.0 (Ar); 129.2 (Ar); 128.9 (2C, Ar); 128.7 (Ar); 128.6 (2C, Ar); 127.3 (Ar); 126.5 (Ar); 126.0 (Ar); 125.4 (Ar); 124.2 (Ar). HPLC-MS (ES$^+$):CH_3CN/H_2O 15:85–95:5, gt: 5.00 min, rt: 6.02 min. $[M+H]^+$ =414.

1.34. 5-amino-1-(3,4-dichlorobenzyl)-3-(1-naphthylmethoxy)indazole (**34**). From **26** (0.30 g, 0.63 mmol), hydrazine monohydrated (3.0 mL, 5.25 mmol) and FeO(OH) (0.02 g, 0.20 mmol) in ethanol (60 mL). Reaction time: 18 h. The dried crude was suspended on water (25 mL) and extracted with methylene chloride (3 x 10 mL). The organic phase was dried with Mg_2SO_4 and filtered. The organic phase was concentrated until 5 mL and hexane (25 mL) was added. The resulting suspension was filtered and the

solid obtained was washed with hexane and air-dried. Yield: (0.19 g, 67%). Mp 150.2 ºC ^{1}H-NMR (300 MHz, $CDCl_3$) δ: 8.18 – 8.16 (m, 1H, Ar); 7.91 – 7.85 (m, 2H, Ar); 7.67 (d, 1H, Ar); 7.54 – 7.45 (m, 3H, Ar); 7.35 – 7.28 (m, 2H, Ar); 7.00 – 6.96 (m, 2H, 7-H, Ar); 6.87 (d, 1H, 4-H); 6.82 (dd, 1H, 6-H); 5.84 (s, 2H, O-CH_2); 5.30 (s, 2H, N1-CH_2); 3.53 (bs, 2H, NH_2). ^{13}C-NMR (75 MHz, $CDCl_3$) δ: 155.8 (C-3); 138.3 (C-5); 137.3 (C-7a); 120.1 (C-6); 114.5 (C-3a); 109.9 (C-7); 104.2 (C-4); 69.6 (O-CH_2); 51.8 (N1-CH_2); 139.1 (Ar); 134.1 (Ar); 133.1 (Ar); 133.0 (Ar); 132.3 (Ar); 131.0 (Ar); 129.5 (Ar); 129.4 (Ar); 129.3 (Ar); 129.0 (Ar); 127.6 (Ar); 126.8 (Ar); 126.2 (Ar); 125.7 (Ar); 124.4 (Ar). HPLC-MS (ES^+): CH_3CN/H_2O 15:85–95:5, gt: 5.00 min, rt: 4.97, $[M+H]^+$ = 448.

Table S1. ^{1}H-NMR spectral data (δ) of compounds **1-12**, **21-26**, **28-34**.

Compd.	4-H	6-H	7-H	O-CH_2	N1-CH_2	Other signals
**1**[a]	6.54	6.83	7.31 – 7.19	5.86	5.40	8.22; 7.99 – 7.92; 7.74; 7.59 – 7.48; 7.31 – 7.19; 3.08; 2.40 - 2.36; 1.75; 1.52; 1.37
**2**[a]	6.65	6.89	7.24	5.49	5.38	7.54 – 7.51; 7.47; 7.40 – 7.30; 7.22; 7.17; 6.63; 3.18; 2.54 - 2.50; 1.86; 1.62; 1.45
**3**[b]	6.69	6.91 – 6.87	7.23 – 7.18	5.78	5.21	8.12; 7.84 – 7.78; 7.62; 7.47 – 7.40; 7.23– 7.18; 6.91 – 6.87; 6.66; 3.99; 3.02; 2.36 – 2.30; 1.86 – 1.80; 1.65 – 1.57; 1.49
**4**[b]	6.79 - 6.70	6.86	7.25 – 7.17	5.57	5.25	7.97; 7.88 - 7.83; 7.64; 7.52 – 7.46; 7.25 – 7.17; 6.96; 6.79 – 6.70; 3.79; 3.16; 2.46 - 2.38; 1.81; 1.62 – 1.56; 1.45
**5**[a]	6.66	6.83	7.23 – 7.18	5.85	5.55	8.33; 7.91- 7.88; 7.67- 7.55; 7.40 - 7.35; 7.23 – 7.18; 3.16; 2.46 - 2.41; 1.83 – 1.78; 1.62 – 1.56; 1.46
**6**[c]	6.52	6.84	7.26	5.54	5.51	8.03; 7.94 – 7.80; 7.72 – 7.63; 7.54 – 7.44; 7.37; 3.04; 2.87; 1.81; 1.62; 1.45
**7**[b]	7.71	7.58 – 7.32	7.18 – 7.13	5.43	5.40	7.98; 7.58 – 7.32; 7.18 – 7.13
**8**[b]	7.69	7.40	7.10	5.86	5.41	8.16; 7.90 – 7.85; 7.53 – 7.45; 7.31 – 7.25; 7.18; 6.22; 3.32; 1.17
**9**[b]	7.66	7.52 – 7.30	7.09 – 7.05	5.82	5.32	8.12; 7.90 – 7.84; 7.66; 7.52 – 7.30; 7.24; 7.09 – 7.05; 6.97; 6.17; 3.32
**10**[b]	7.66	7.53 – 7.43	7.08	5.83	5.33	8.16; 7.90 – 7.85; 7.53 – 7.43; 7.39 – 7.22; 6.97; 6.41
**11**[b]	7.66	7.38 – 7.29	7.08	5.47	5.39	7.49 – 7.47; 7.38 – 7.29; 7.08; 6.98; 6.48; 3.69; 3.52; 2.46; 2.33
**12**[b]	7.67 – 7.49	7.42	7.03	5.84	5.45	8.16; 7.87 – 7.85; 7.78 – 7.76; 7.67 – 7.49; 7.33; 7.27; 7.20; 6.96; 6.15; 3.44; 1.95
**21**[b]	6.69	6.91 – 6.87	7.23 – 7.18	5.78	5.21	8.12; 7.84 – 7.78; 7.62; 7.47 – 7.40; 7.23– 7.18; 6.91 – 6.87; 6.66; 3.99; 3.02; 2.36 – 2.30; 1.86 – 1.80; 1.65 – 1.57; 1.49
**22**[b]	6.79 - 6.70	6.86	7.25 – 7.17	5.57	5.25	7.97; 7.88 - 7.83; 7.64; 7.52 – 7.46; 7.25 – 7.17; 6.96; 6.79 – 6.70; 3.79; 3.16; 2.46 - 2.38; 1.81; 1.62 – 1.56; 1.45
**23**[b]	6.79 - 6.70	6.86	7.25 – 7.17	5.57	5.25	7.97; 7.88 - 7.83; 7.64; 7.52 – 7.46; 7.25 – 7.17; 6.96; 6.79 – 6.70; 3.79; 3.16; 2.46 - 2.38; 1.81; 1.62 – 1.56; 1.45
**24**[b]	6.66	6.83	7.23 – 7.18	5.85	5.55	8.33; 7.91- 7.88; 7.67- 7.55; 7.40 - 7.35; 7.23 – 7.18; 3.16; 2.46 - 2.41; 1.83 – 1.78; 1.62 – 1.56; 1.46
**25**[b]	6.52	6.84	7.26	5.54	5.51	8.03; 7.94 – 7.80; 7.72 – 7.63; 7.54 – 7.44; 7.37; 3.04; 2.87; 1.81; 1.62; 1.45
**26**[b]	6.69	6.91 – 6.87	7.23 – 7.18	5.78	5.21	8.12; 7.84 – 7.78; 7.62; 7.47 – 7.40; 7.23– 7.18; 6.91 – 6.87; 6.66; 3.99; 3.02; 2.36 – 2.30; 1.86 – 1.80; 1.65 – 1.57; 1.49
**28**[b]	6.69	6.91 – 6.87	7.23 – 7.18	5.78	5.21	8.12; 7.84 – 7.78; 7.62; 7.47 – 7.40; 7.23– 7.18; 6.91 – 6.87; 6.66; 3.99; 3.02; 2.36 – 2.30; 1.86 – 1.80; 1.65 – 1.57; 1.49

**29**[b]	6.69	6.91 – 6.87	7.23 – 7.18	5.78	5.21	8.12; 7.84 – 7.78; 7.62; 7.47 – 7.40; 7.23– 7.18; 6.91 – 6.87; 6.66; 3.99; 3.02; 2.36 – 2.30; 1.86 – 1.80; 1.65 – 1.57; 1.49
**30**[b]	6.52	6.84	7.26	5.54	5.51	8.03; 7.94 – 7.80; 7.72 – 7.63; 7.54 – 7.44; 7.37; 3.04; 2.87; 1.81; 1.62; 1.45
**31**[b]	6.66	6.83	7.23 – 7.18	5.85	5.55	8.33; 7.91- 7.88; 7.67- 7.55; 7.40 - 7.35; 7.23 – 7.18; 3.16; 2.46 - 2.41; 1.83 – 1.78; 1.62 – 1.56; 1.46
**32**[b]	6.66	6.83	7.23 – 7.18	5.85	5.55	8.33; 7.91- 7.88; 7.67- 7.55; 7.40 - 7.35; 7.23 – 7.18; 3.16; 2.46 - 2.41; 1.83 – 1.78; 1.62 – 1.56; 1.46
**33**[b]	6.52	6.84	7.26	5.54	5.51	8.03; 7.94 – 7.80; 7.72 – 7.63; 7.54 – 7.44; 7.37; 3.04; 2.87; 1.81; 1.62; 1.45
**34**[b]	6.79 - 6.70	6.86	7.25 – 7.17	5.57	5.25	7.97; 7.88 - 7.83; 7.64; 7.52 – 7.46; 7.25 – 7.17; 6.96; 6.79 – 6.70; 3.79; 3.16; 2.46 - 2.38; 1.81; 1.62 – 1.56; 1.45

[a] Acetone-d_6; [b] $CDCl_3$; [c]: DMSO-d_6.

Table S2. ^{13}C-NMR spectral data (δ) of compounds **1-12**, **21-26** and **28-34.**

Compd.	C-3	C-3a	C-4	C-5	C-6	C-7	C-7a	O-CH$_2$	N1-CH$_2$	Other signals
**1**[a]	155.6	114.6	97.5	144.2	120.0	110.9	139.4	69.5	52.8	137.6, 134.8, 134.1, 132.9, 129.7, 129.4, 129.2 (2C), 128.2, 128.1 (2C), 128.0, 127.2, 126.7, 126.2, 124.9, 58.2; 55.3 (2C), 44.1, 26.7 (2C), 26.5, 25.1
**2**[a]	155.1	113.6	96.6	143.6	119.4	109.8	138.7	70.1	49.8	137.6, 136.9, 132.4, 129.2, 128.3, 128.1, 127.9, 127.8, 127.0 (Ar), 57.3, 54.4 (2C), 43.2, 25.8 (2C), 25.7, 24.3
**3**[b]	156.0	114.6	98.8	143.4	119.9	109.9	137.3	69.6	51.8	138.5, 134.2, 133.1, 133.0, 132.3, 131.9, 130.9, 129.5, 129.4, 129.0, 127.7, 126.9, 126.8, 126.3, 125.7, 124.6, 58.7, 55.0 (2C), 45.1, 26.5 (2C), 26.0, 24.8
**4**[b]	155.5	114.2	98.4	143.1	119.6	109.6	137.0	70.9	51.4	138.2, 134.8, 133.7, 133.4, 133.2, 132.6, 131.5, 130.6, 129.0, 128.3, 128.1, 127.8, 127.2, 126.5, 126.2, 126.1, 126.0, 58.4, 54.8 (2C), 44.8, 26.2 (2C), 25.8, 24.6
**5**[a]	154.8	114.2	97.2	144.4	120.1	111.1	138.8	68.5	51.3	137.7, 134.8, 134.7, 133.3, 132.2, 131.6, 130.6, 129.4, 128.9, 128.7, 127.0, 126.6, 126.4, 126.2, 124.6, 58.1, 55.2 (2C), 44.1, 26.6 (2C), 26.5, 25.1
**6**[a]	155.4	112.1	98.2	143.4	120.0	111.2	137.9	71.1	53.0	136.8, 136.2, 134.3, 133.7, 128.9, 128.9, 128.8, 128.6, 128.5, 128.4, 127.9, 127.1, 127.0, 127.0, 126.9, 126.8, 126.7, 126.7, 126.6, 126.4, 56.2, 53.6 (2C), 42.5, 23.9, 23.4 (2C), 22.4
**7**[b]	156.0	113.2	109.3	132.3	122.5	112.3	138.0	70.8	52.7	164.3, 137.6, 137.3, 137.0, 134.2, 132.3, 129.4, 129.3, 128.8 (2C), 128.6 (2C), 128.2 (2C), 128.1, 128.0, 128.7, 127.1 (2C)
**8**[b]	155.7	113.1	109.1	131.8	123.9	111.6	137.5	69.1	52.5	155.3, 139.2, 133.7, 132.6, 131.4, 129.0, 128.6 (2C), 128.5, 127.5, 127.1, 127.0 (2C), 126.4, 125.8, 125.3, 124.1, 41.6 (2C), 13.9 (2C)
**9**[b]	156.0	113.3	109.0	131.9	123.4	111.2	136.0	69.1	52.0	155.2, 143.1, 139.2, 133.8, 132.6, 131.3, 131.1, 130.5 (2C, Ar); 129.6, 129.2, 128.9 (2C, Ar); 128.7, 128.5 (2C), 128.0, 127.6 (2C), 127.3, 126.6, 125.4, 124.1, 37.4
**10**[b]	156.2	113.4	108.9	131.8	123.1	111.2	137.8	69.2	51.5	154.2, 142.5, 139.2, 133.8, 132.8, 132.4, 131.9, 131.4, 131.1, 130.7, 129.7 (4C), 129.2, 129.1, 128.7, 127.6 (3C), 127.3, 126.8 (2C), 126.6, 126.5, 126.0, 125.4, 124.0
**11**[b]	156.1	113.3	109.0	131.5	124.1	112.1	137.0	70.7	50.4	155.8, 139.5, 137.7, 133.2, 130.4, 129.4, 128.5, 128.1 (2C), 128.0 (2C), 127.6, 126.3, 54.7 (2C), 46.0, 43.9 (2C)
**12**[b]	155.2	113.1	109.5	131.6	123.4	111.1	137.5	68.1	51.1	154.7, 139.4, 133.8, 133.1, 132.8, 131.2, 131.1, 129.6, 128.9, 128.4, 127.4, 127.2, 126.5, 126.0, 125.5, 125.3, 123.3, 45.9 (2C), 25.8 (2C)
**21**[b]	158.3	113.1	120.1	141.8	123.5	109.3	143.6	71.7	51.1	136.6, 136.5, 133.9, 131.3, 130.4, 129.0, 128.8 (2C), 128.7, 128.0 (2C), 127.0
**22**[b]	157.1	112.6	118.6	141.3	122.8	109.4	143.2	68.6	51.8	136.5, 134.0, 133.4, 131.4, 131.1, 130.2, 129.2, 129.0, 127.5 (2C, Ar), 127.4, 126.9, 129.3, 125.8, 125.4, 123.1
**23**[b]	157.9	113.0	118.9	141.5	123.1	108.7	142.9	71.5	51.8	136.3, 133.7, 133.3, 133.1, 132.4, 131.0, 129.3, 128.5, 128.2, 127.9, 127.5, 126.6, 126.5 (2C), 125.9 (Ar)

**24**[b]	157.8	112.8	118.8	141.2	122.8	109.0	142.9	69.7	53.1	136.2, 133.9, 131.9, 131.8, 129.5, 129.0 (2C), 128.8, 128.2, 127.7, 127.3 (2C), 126.8, 126.1, 125.4, 123.9
**25**[b]	157.9	112.9	118.9	141.3	123.0	108.8	142.9	69.7	52.4	134.7, 134.2, 133.9, 131.9, 131.7, 129.6 (2C), 129.2, 128.9, 128.7 (2C), 127.7, 126.8, 126.2, 125.3, 123.0
**26**[b]	158.2	113.3	119.2	141.8	123.4	109.0	142.7	71.8	51.4	143.2, 136.6, 133.9, 133.6, 133.4, 132.7, 131.2, 129.6, 128.8, 128.4, 128.2, 127.8, 126.9, 126.8 (2C), 126.2
**28**[b]	155.1	114.0	103.2	139.4	119.5	110.0	137.8	70.9	52.9	135.3, 134.9, 133.4, 133.2, 132.9, 128.5, 128.4, 128.3, 128.2, 128.1, 128.0, 127.8, 127.1, 126.2, 126.1, 125.9, 125.8, 125.3
**29**[b]	155.7	114.2	103.7	139.7	120.1	110.0	138.3	71.0	50.6	138.1, 137.6, 133.4, 130.7, 129.6, 128.8, 128.5, 128.4, 127.8, 126.6
**30**[b]	154.3	113.6	102.9	139.5	119.6	110.2	137.8	68.2	51.1	137.6, 133.8, 133.1, 133.0, 131.3, 131.2, 129.7, 128.8, 128.3, 127.3, 127.3, 126.5, 125.9, 123.3
**31**[b]	155.7	114.5	103.6	138.3	120.0	110.0	137.9	71.2	51.7	140.0, 135.0, 133.7, 133.5, 133.0, 131.9, 130.9, 129.4, 128.6, 128.1, 127.5, 127.4, 126.8, 126.6, 126.5, 126.4
**32**[b]	155.2	113.9	104.1	138.2	119.7	110.0	137.9	69.3	52.7	137.8, 133.9, 132.9, 132.0, 129.1, 128.7 (3C), 127.6, 127.3, 127.2 (2C), 126.5, 126.0, 125.4, 124.3
**33**[b]	155.3	114.1	103.6	139.1	119.7	109.8	137.7	69.3	52.0	136.3, 133.9, 132.8, 132.0, 129.2, 128.9 (2C), 128.7, 128.6 (2C), 127.3, 126.5, 126.0, 125.4, 124.2
**34**[b]	155.8	114.5	104.2	138.3	120.1	109.9	137.3	69.6	51.8	139.1, 134.1, 133.1, 133.0, 132.3, 131.0, 129.5, 129.4, 129.3, 129.0, 127.6, 126.8, 126.2, 125.7, 124.4

[a] Acetone-d_6; [b] $CDCl_3$.

1

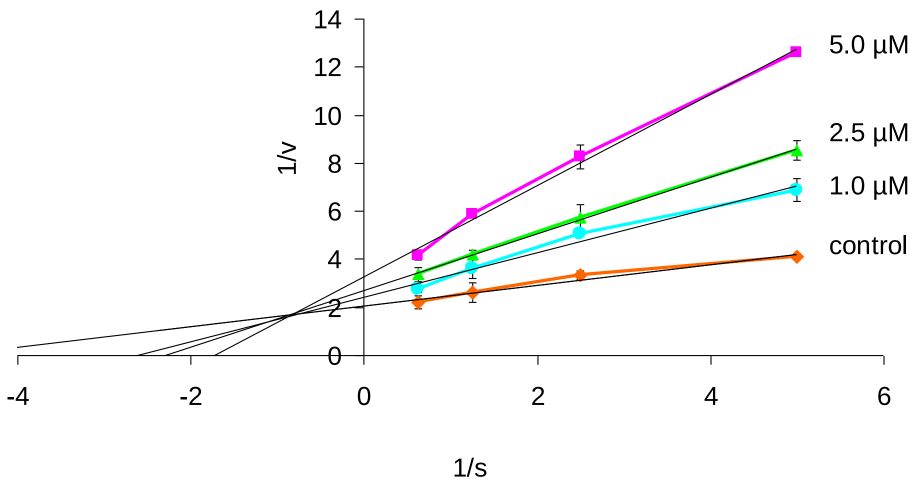
14
12
10
8
6
4
2
0
1/v
-4
-2
0
2
4
6
1/s
5.0 µM
2.5 µM
1.0 µM
control

2

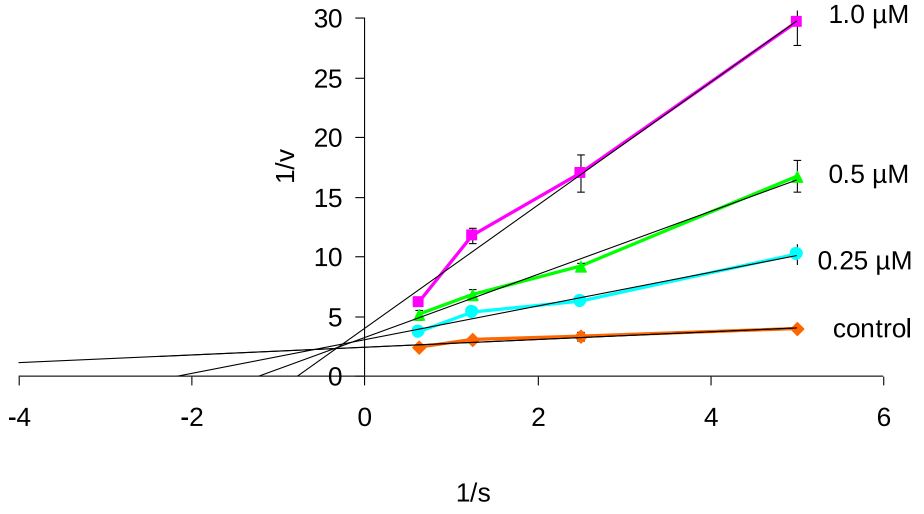
30
25
20
15
10
5
0
1/v
-4
-2
0
2
4
6
1/s
1.0 µM
0.5 µM
0.25 µM
control

3

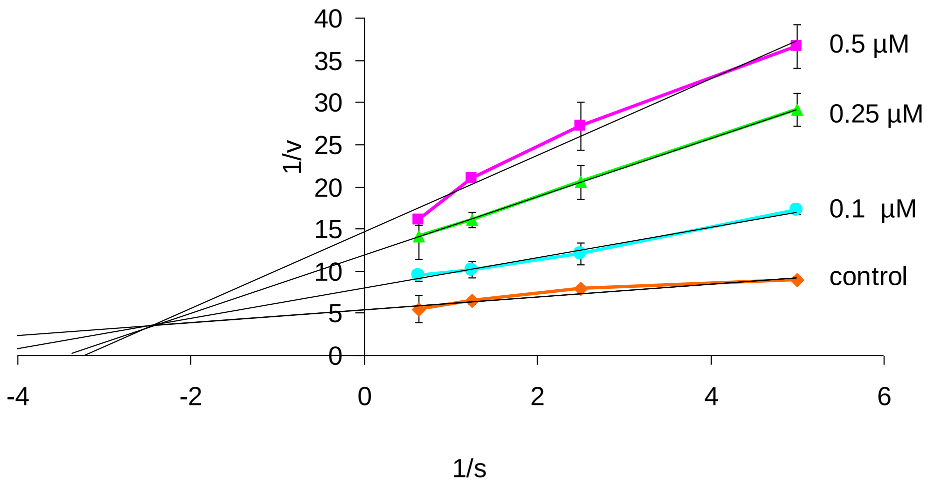
40
35
30
25
20
15
10
5
0
1/v
-4
-2
0
2
4
6
1/s
0.5 µM
0.25 µM
0.1 µM
control

4

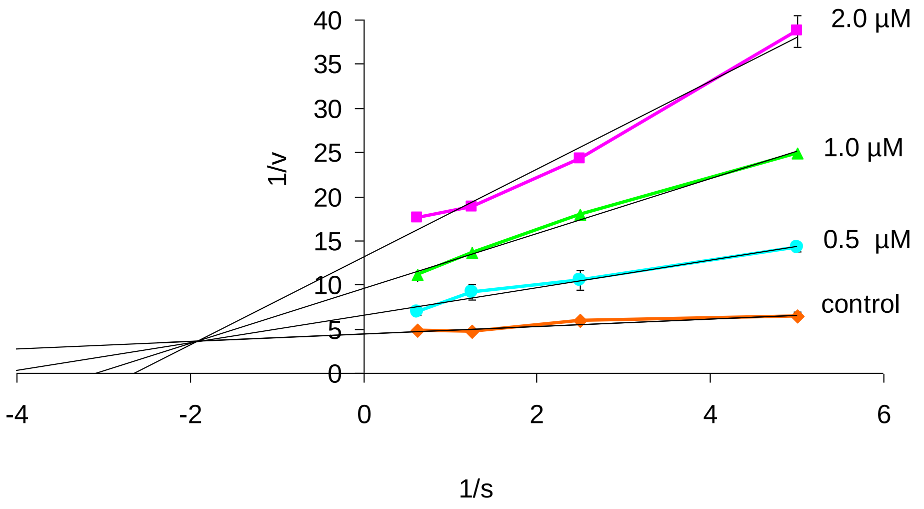

6

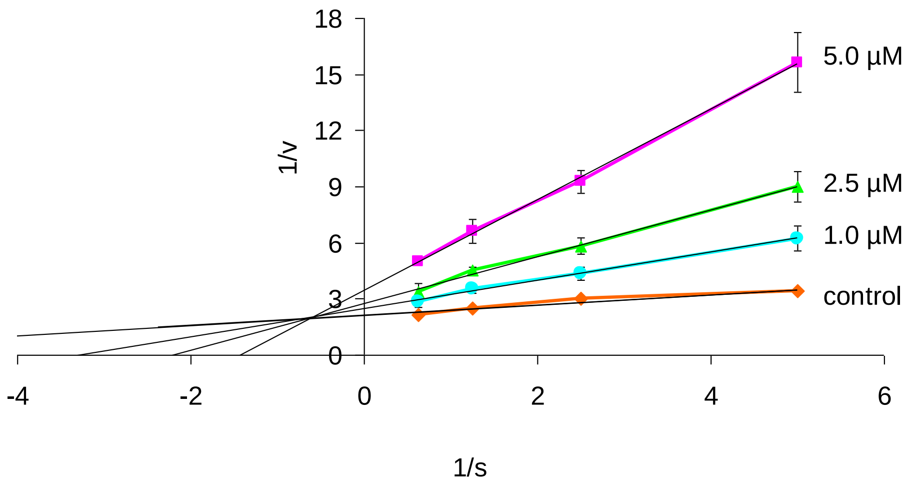

Figure S1. Kinetic study on the mechanism of BuChE inhibition of compounds **1-4 and 6**. Overlaid Lineweaver–Burk reciprocal plots of BuChE initial velocity at increasing substrate concentration in the absence of inhibitor and in the presence of inhibitors are shown. Lines were derived from a weighted least-squares analysis of the data points.

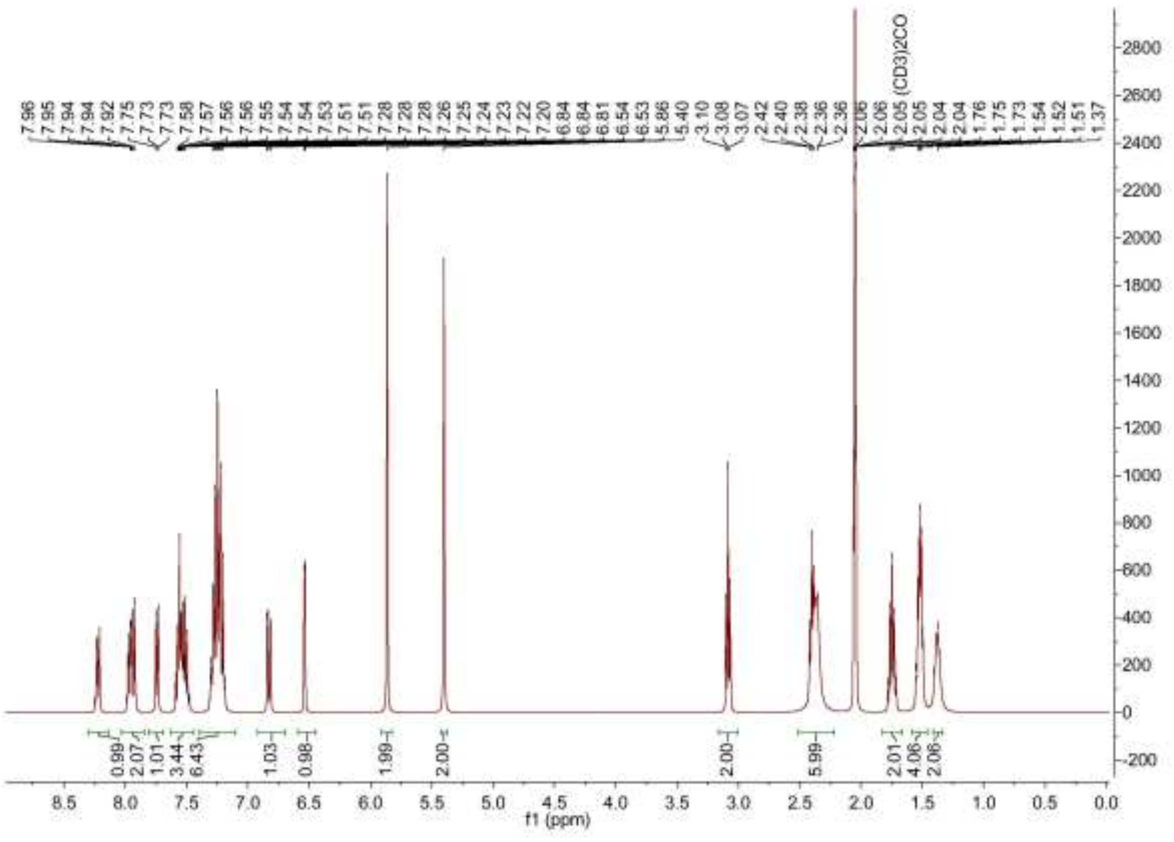

Figure **S2**. ^{1}H-NMR spectrum of **1** (400 MHz, acetone-d_6).

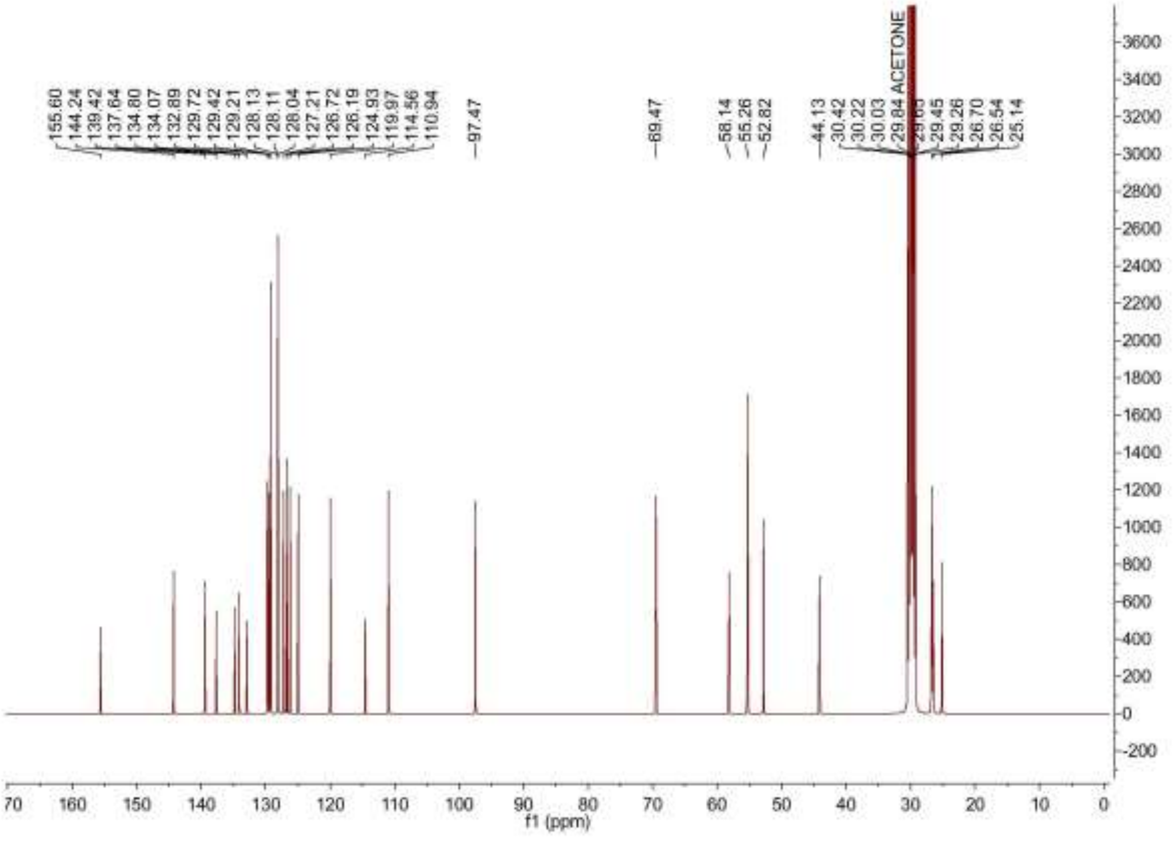

Figure **S3**. ^{13}C-NMR spectrum of **1** (100 MHz, acetone-d_6).

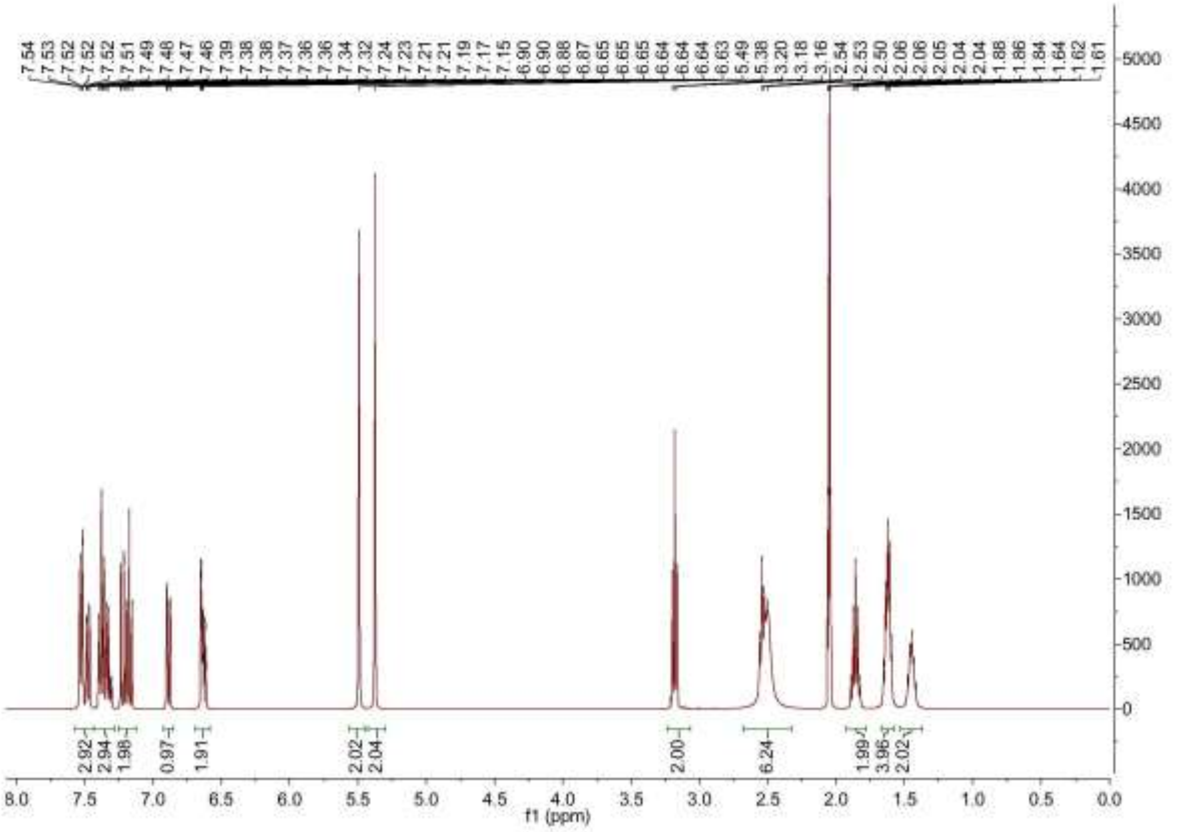

***Figure* S4**. ^{1}H-NMR spectrum of **2** (400 MHz, acetone-d$_6$).

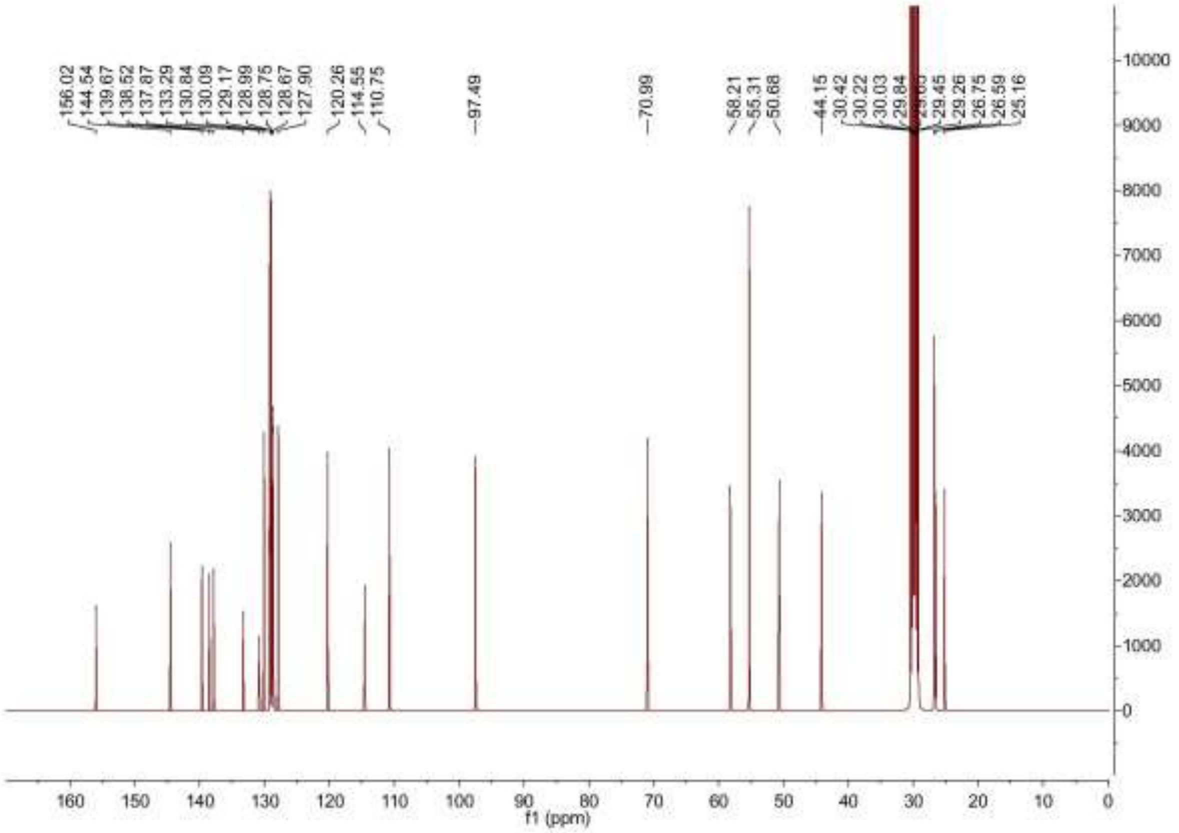

***Figure* S5**. ^{13}C-NMR spectrum of **2** (100 MHz, acetone-d$_6$).

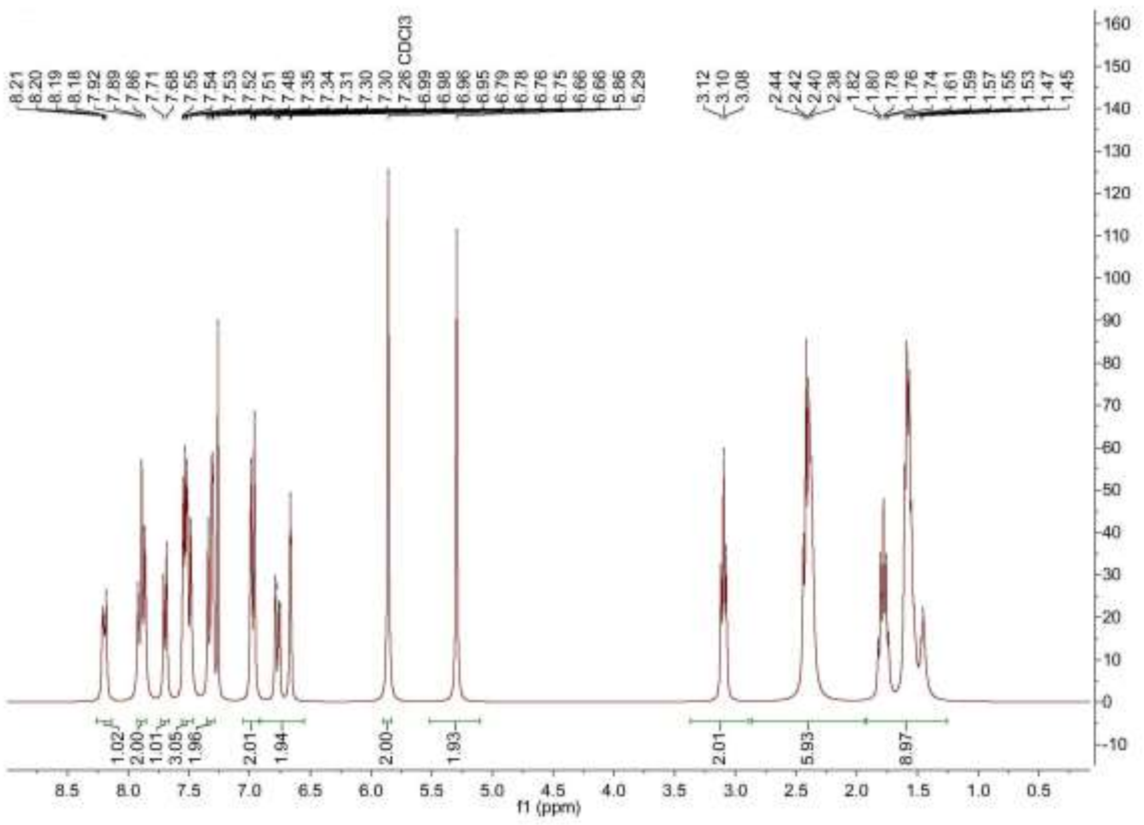

***Figure* S6**. ^{1}H-NMR spectrum of **3** (400 MHz, $CDCl_3$).

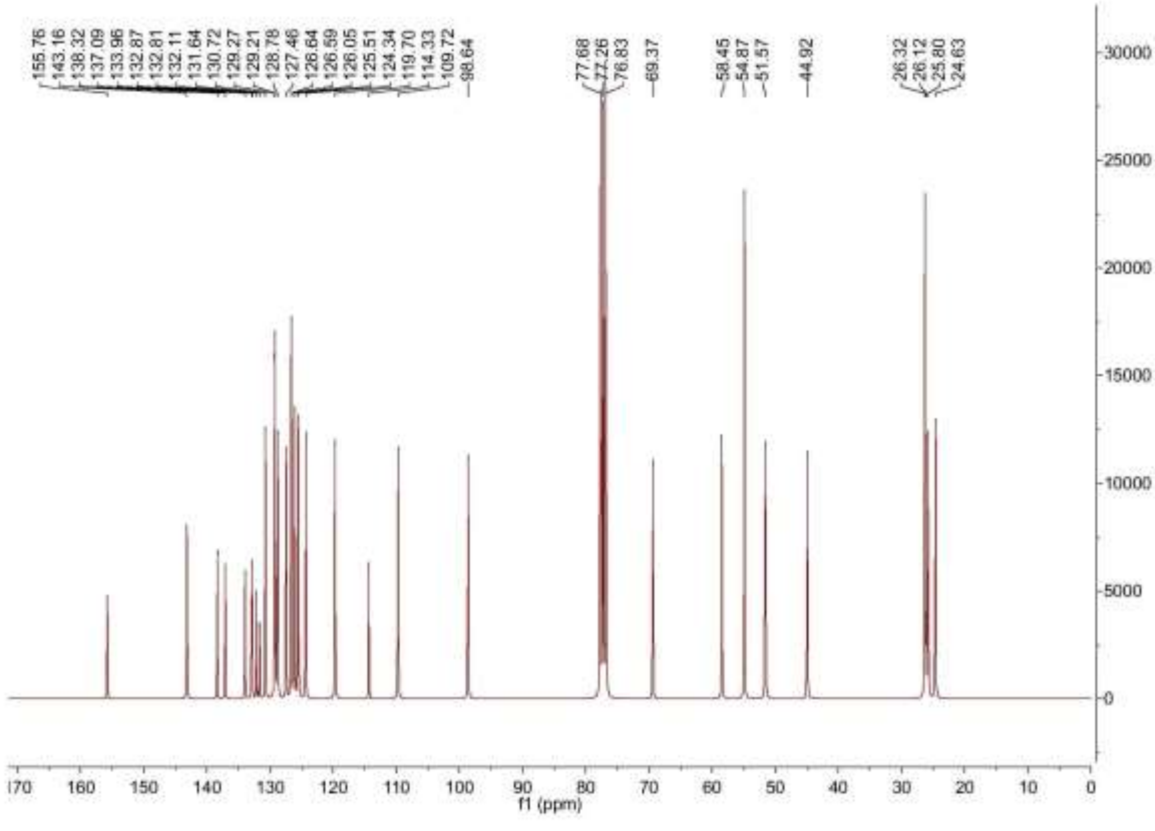

***Figure* S7**. ^{13}C-NMR spectrum of **3** (100 MHz, $CDCl_3$).

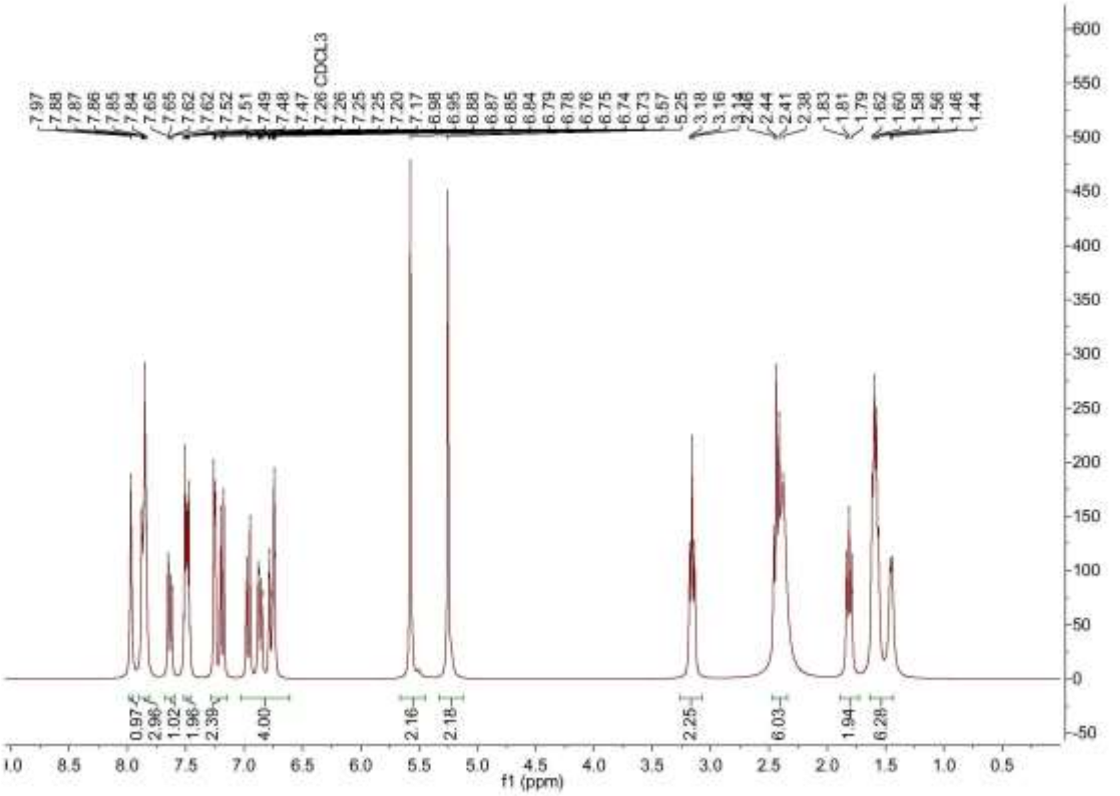

***Figure* S8**. ^{1}H-NMR spectrum of **4** (400 MHz, $CDCl_3$).

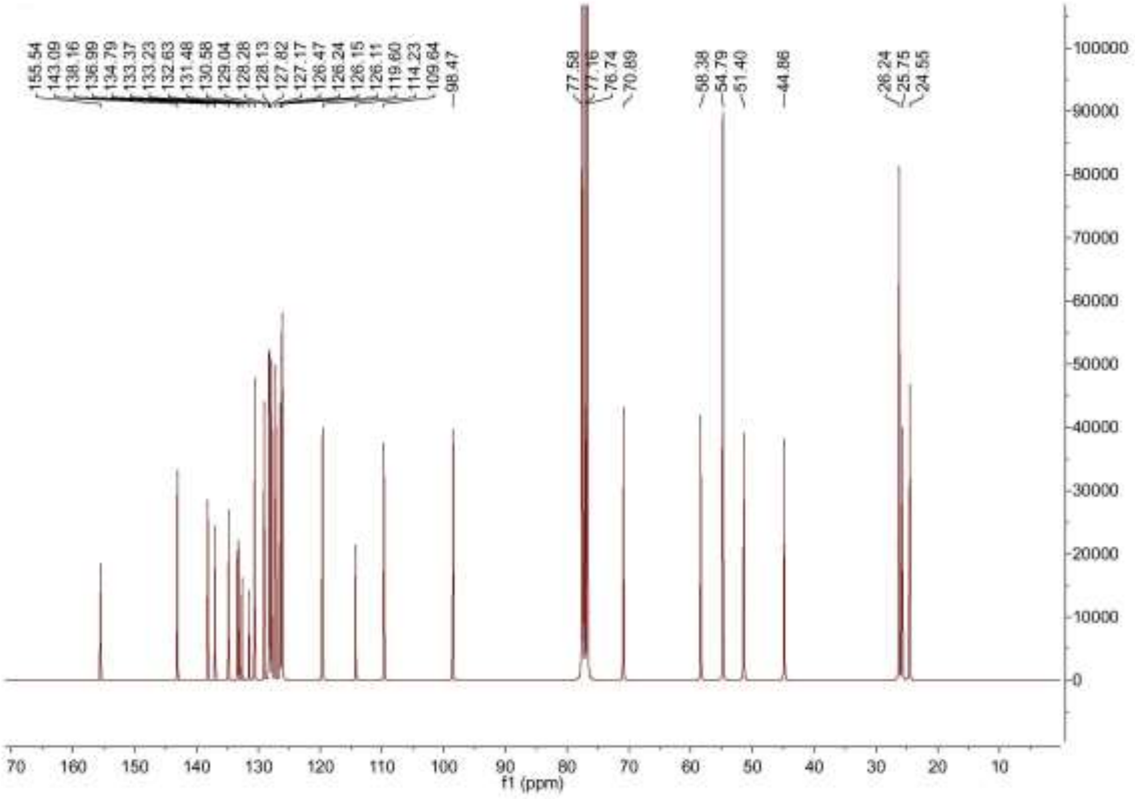

***Figure* S9.** ^{13}C-NMR spectrum of **4** (100 MHz, $CDCl_3$).

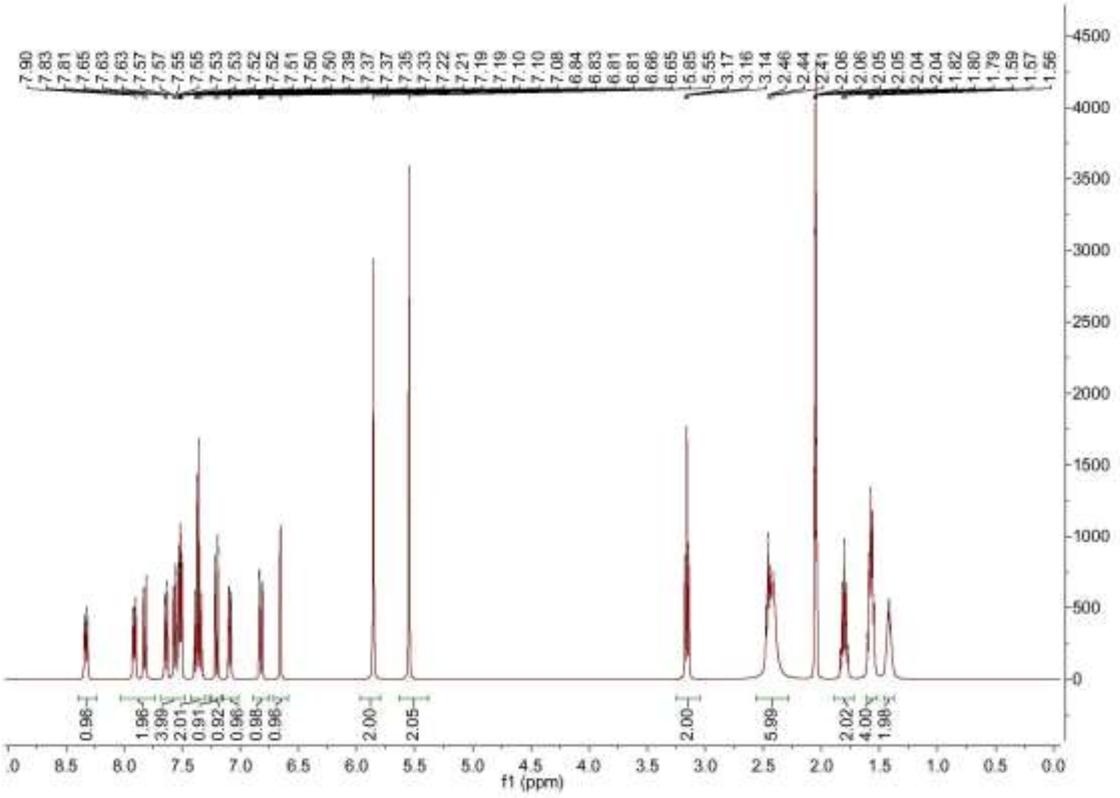

***Figure* S10**. ^{1}H-NMR spectrum of **5** (400 MHz, acetone-d_6).

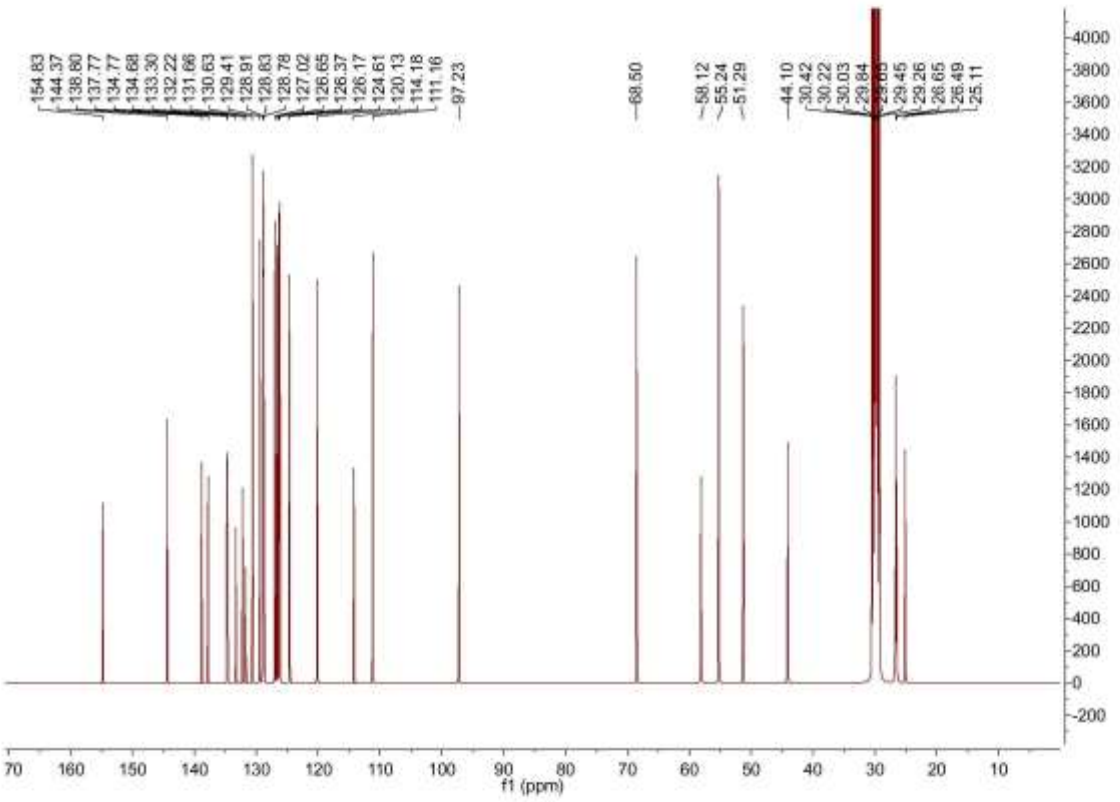

***Figure* S11**. ^{13}C-NMR spectrum of **5** (100 MHz, acetone-d_6).

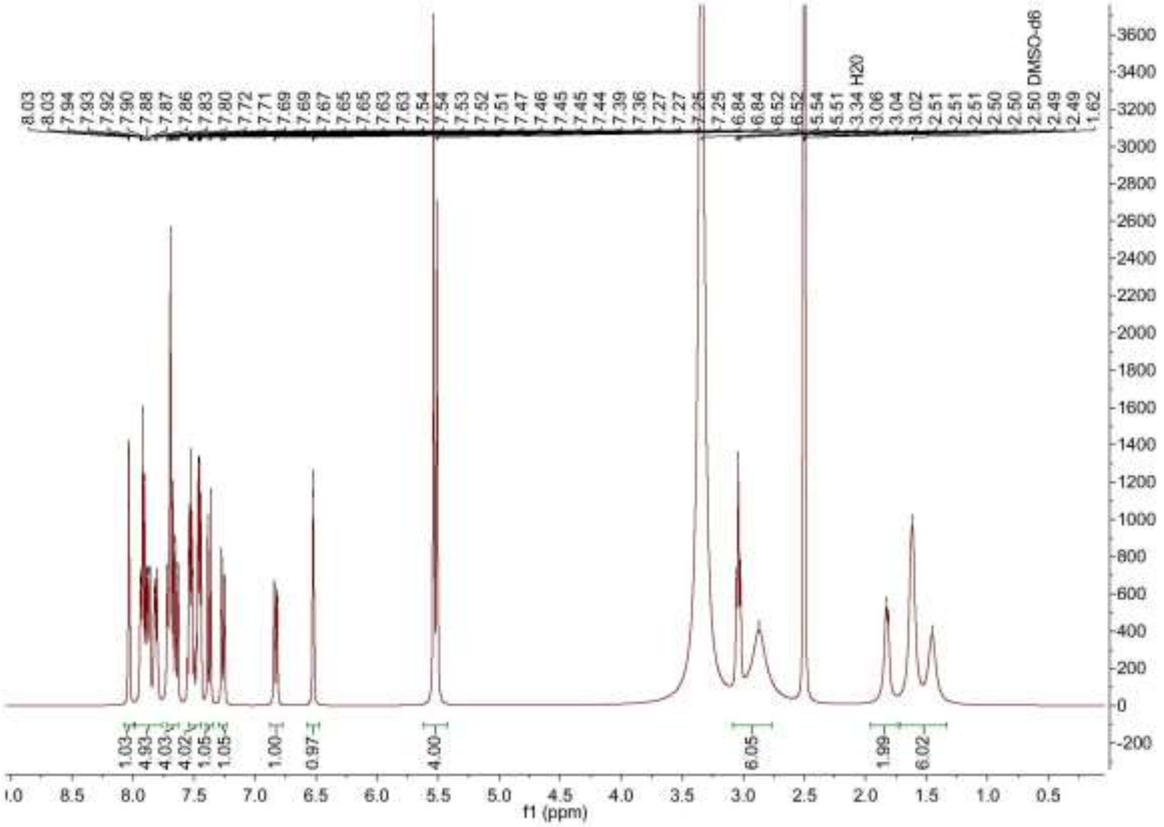

***Figure* S12**. ^{1}H-NMR spectrum of **6** (400 MHz, DMSO-d_6).

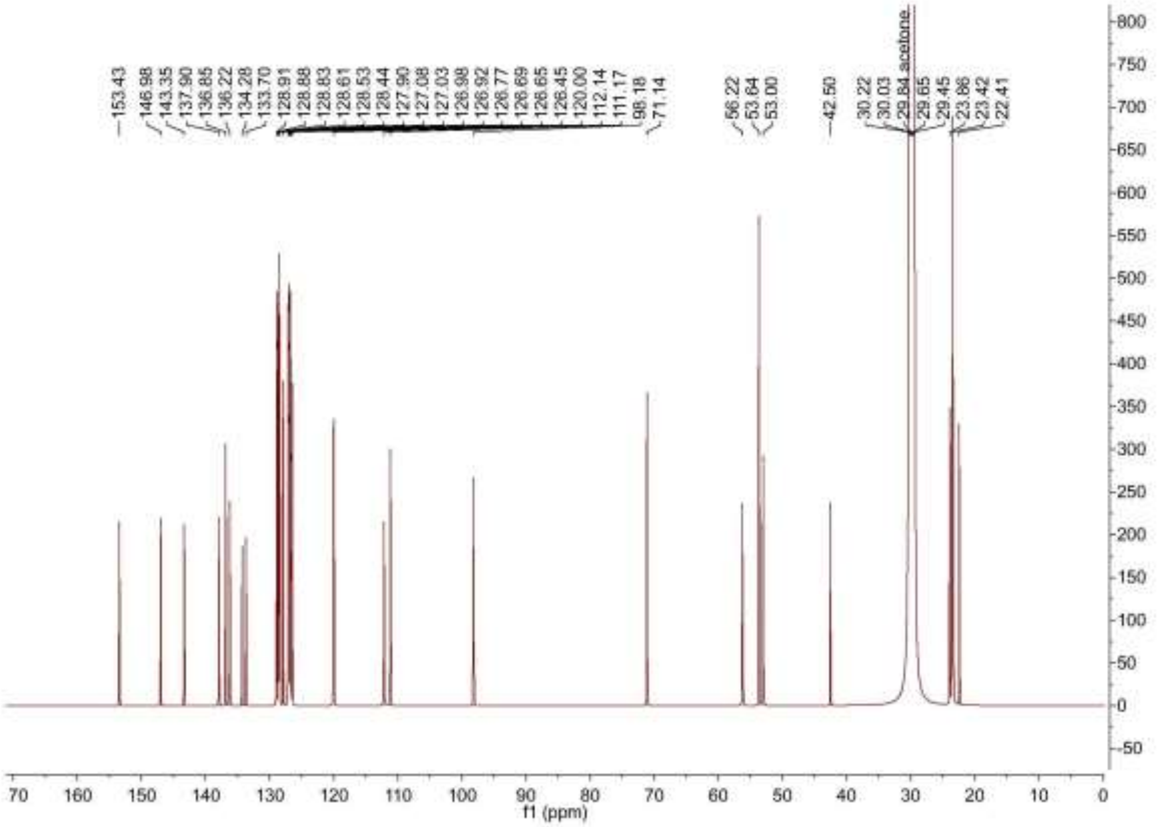

***Figure* S13**. ^{13}C-NMR spectrum of **6** (100 MHz, acetone-d_6).

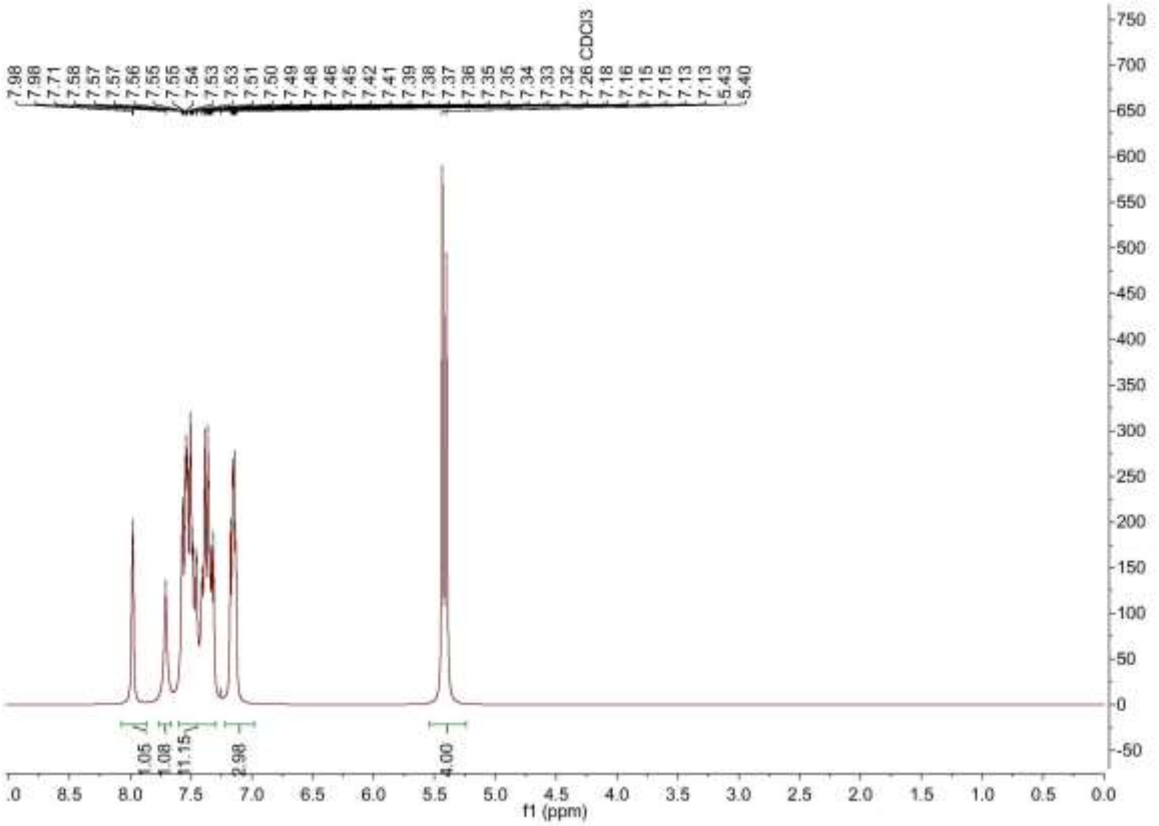

***Figure* S14**. ^{1}H-NMR spectrum of **7** (400 MHz, $CDCl_3$).

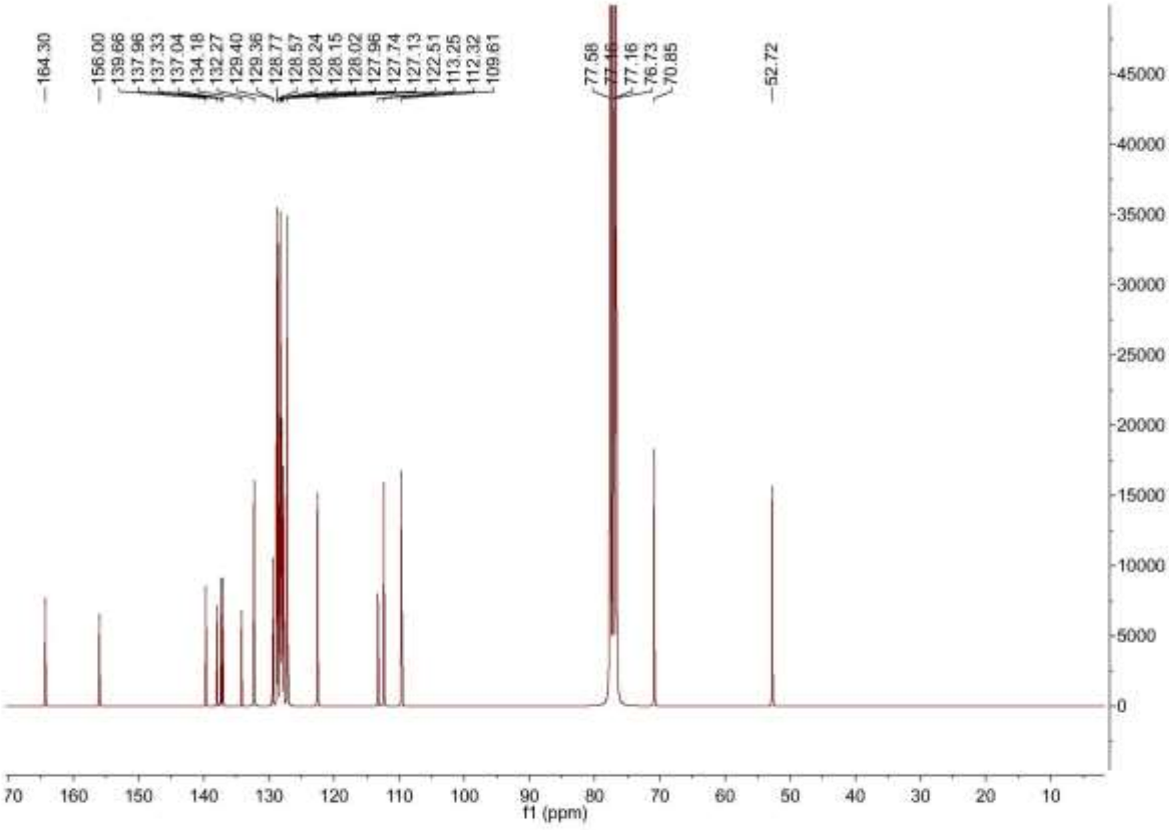

***Figure* S15.** ^{13}C-NMR spectrum of **7** (100 MHz, $CDCl_3$).

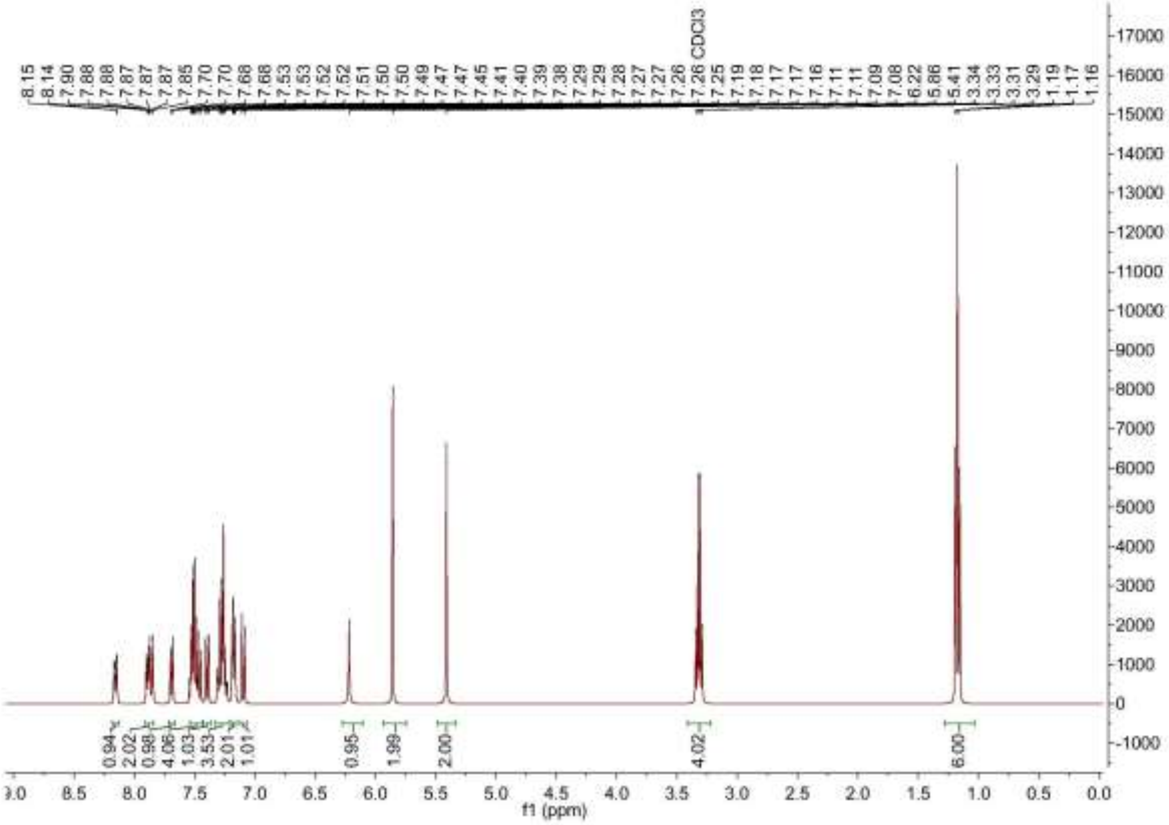

***Figure* S16**. ^{1}H-NMR spectrum of **8** (400 MHz, $CDCl_3$).

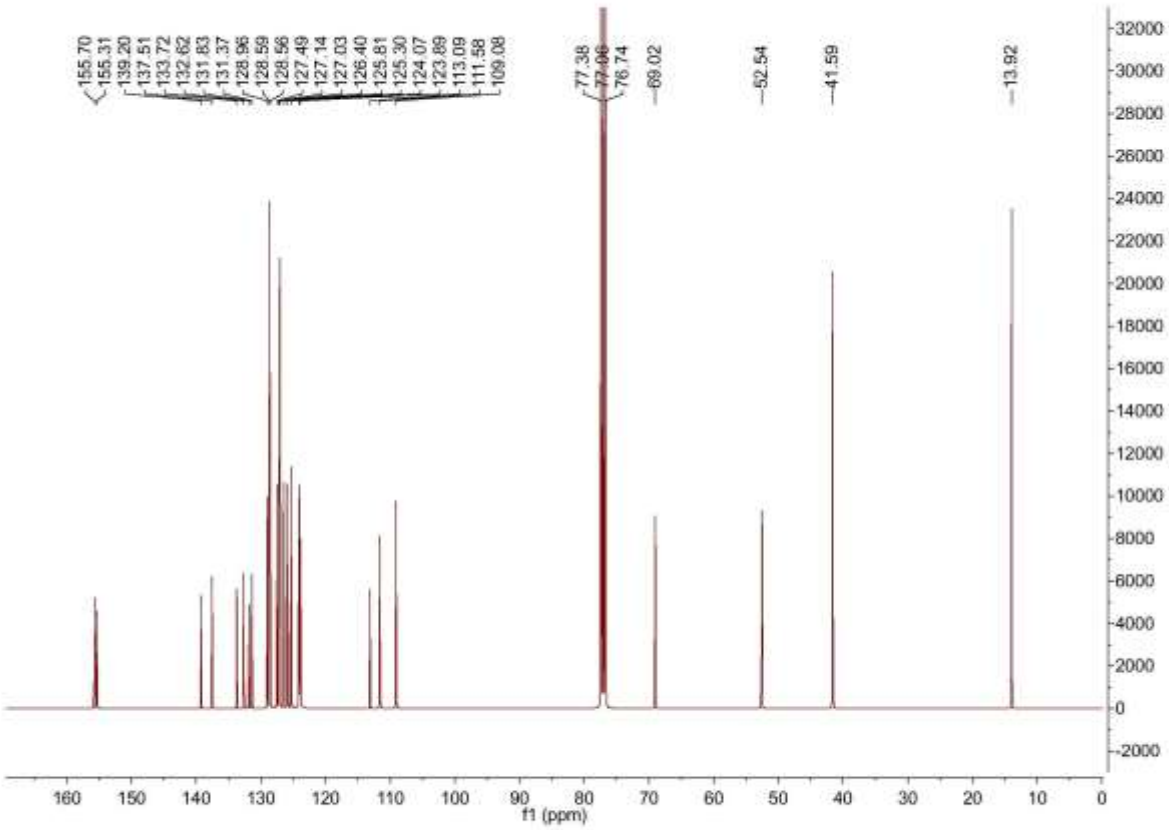

***Figure* S17.** ^{13}C-NMR spectrum of **8** (100 MHz, $CDCl_3$).

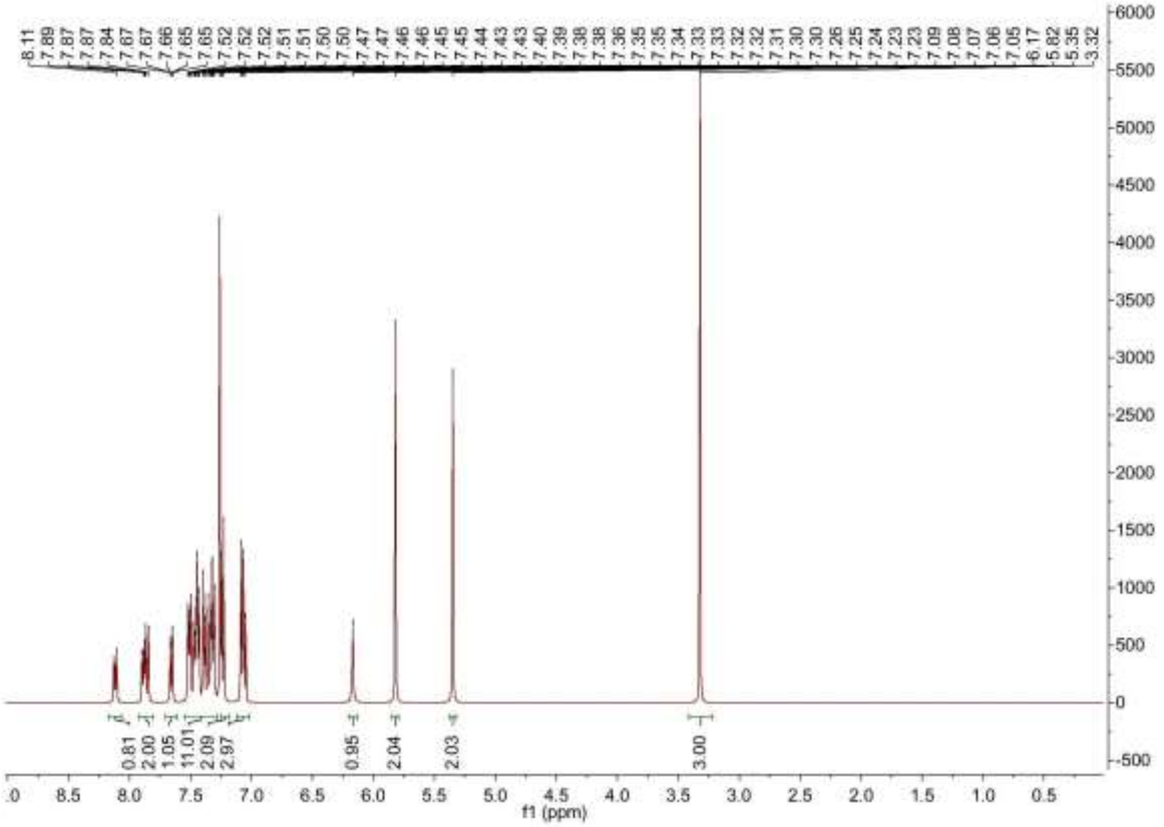

***Figure* S18**. ^{1}H-NMR spectrum of **9** (400 MHz, $CDCl_3$).

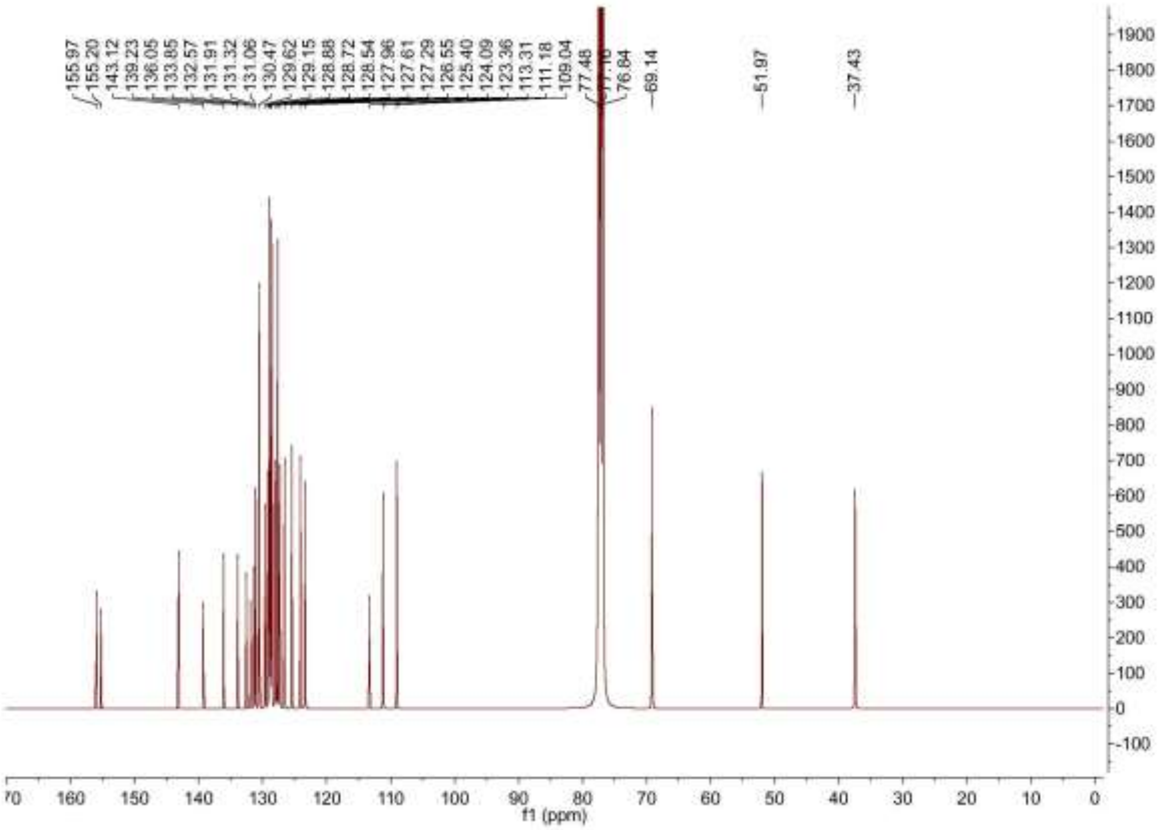

***Figure* S19.** ^{13}C-NMR spectrum of **9** (100 MHz, $CDCl_3$).

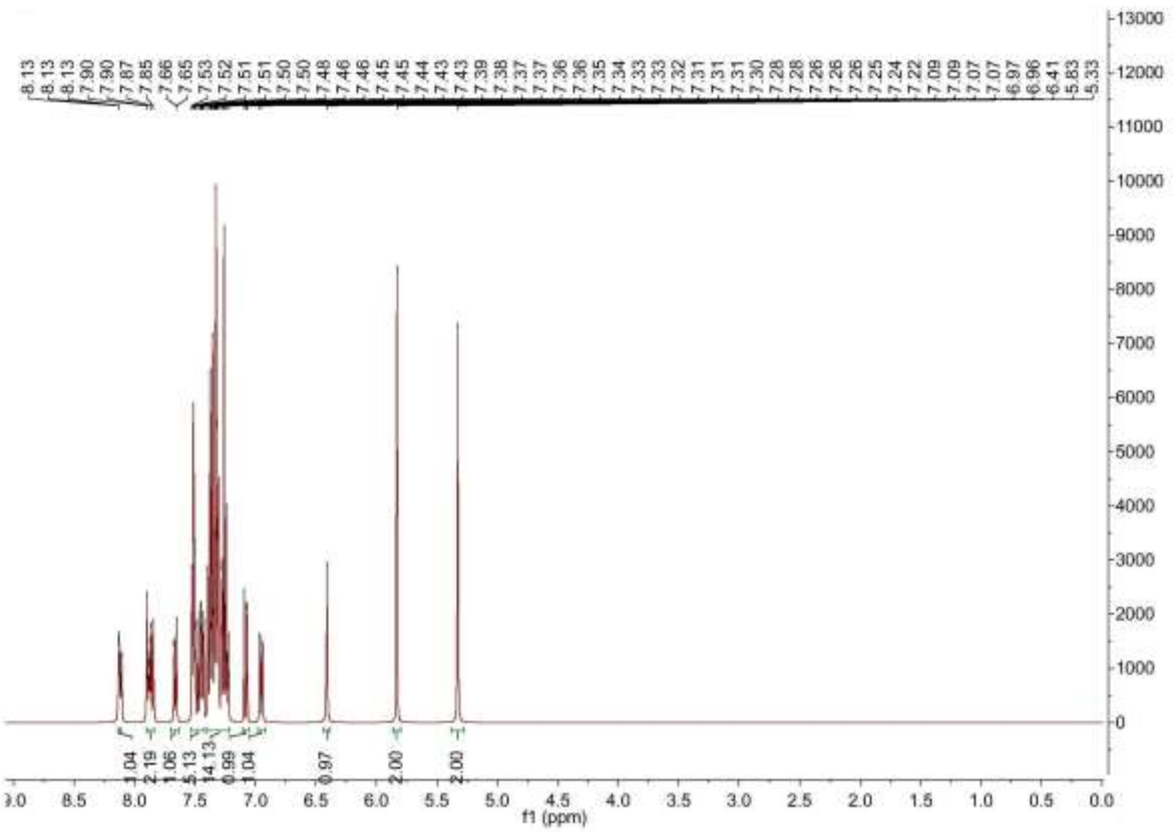

***Figure* S20**. ^{1}H-NMR spectrum of **10** (400 MHz, $CDCl_3$).

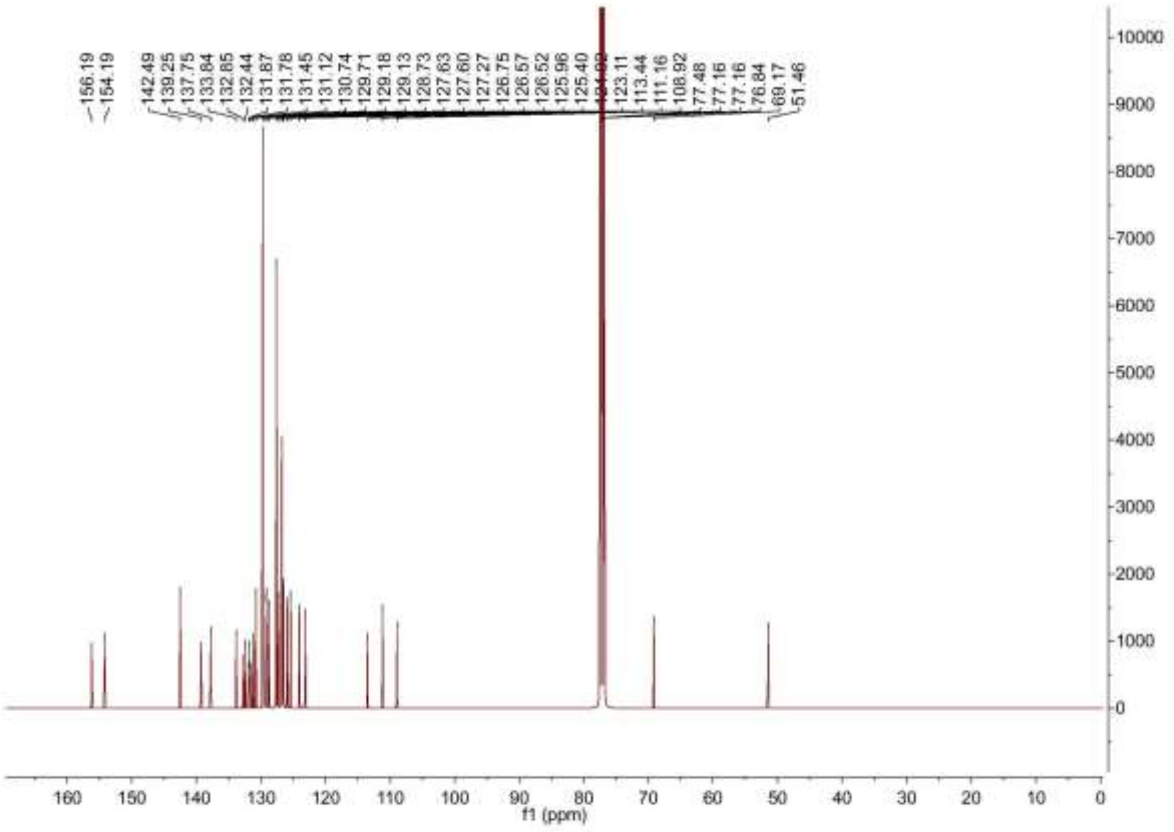

***Figure* S21.** ^{13}C-NMR spectrum of **10** (400 MHz, $CDCl_3$).

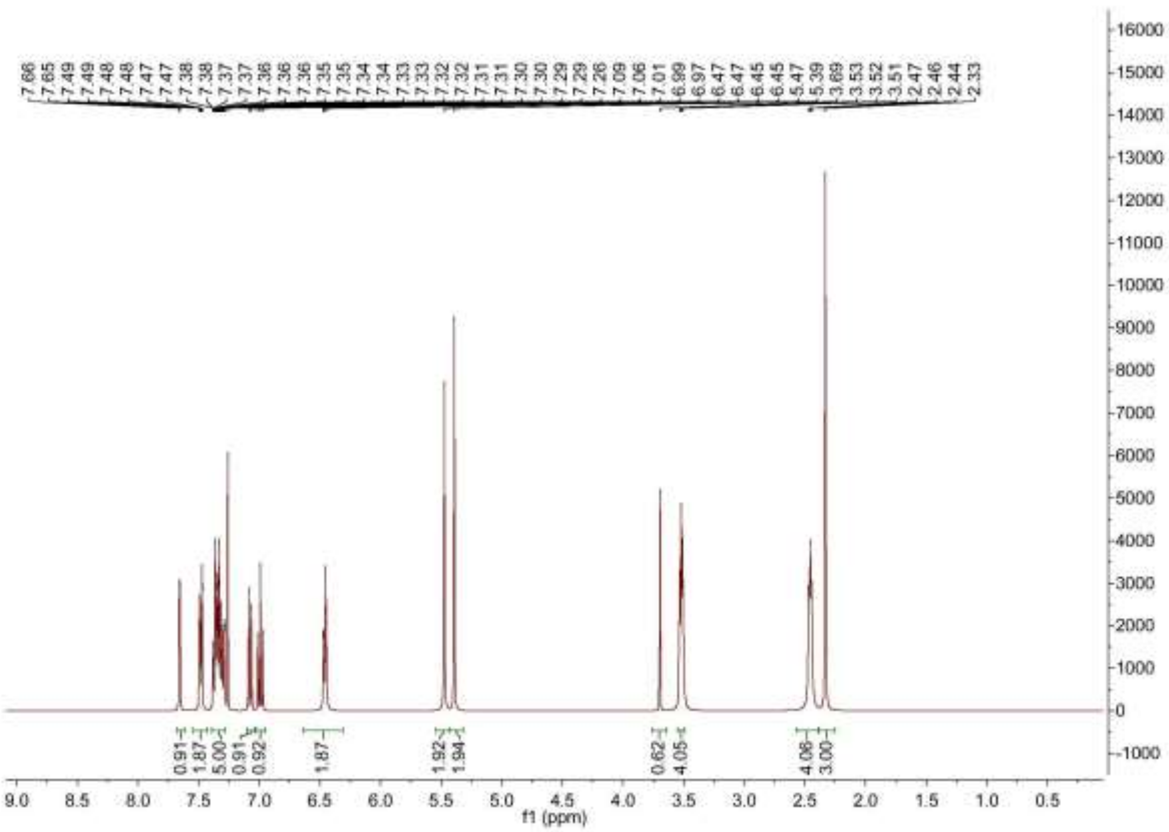

***Figure* S22**. ^{1}H-NMR spectrum of **11** (400 MHz, $CDCl_3$).

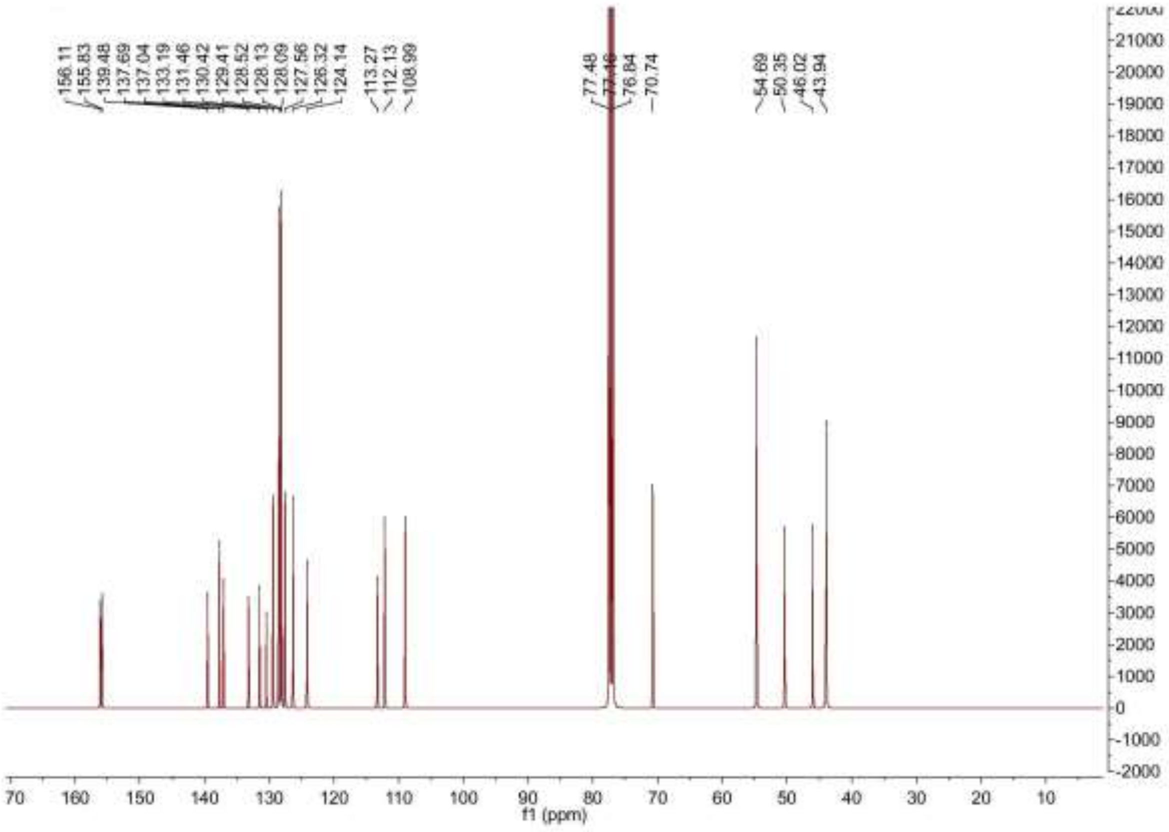

***Figure* S23.** ^{13}C-NMR spectrum of **11** (100 MHz, $CDCl_3$).

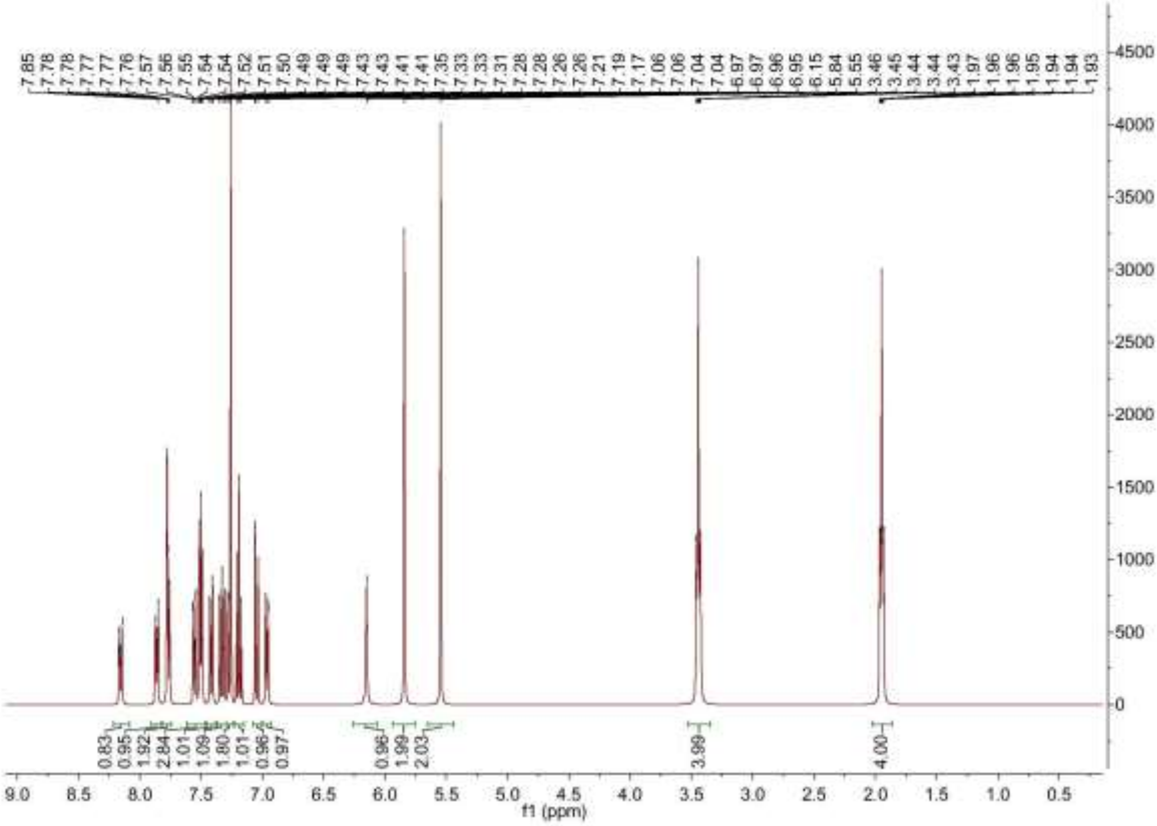

***Figure* S24**. ^{1}H-NMR spectrum of **12** (400 MHz, $CDCl_3$).

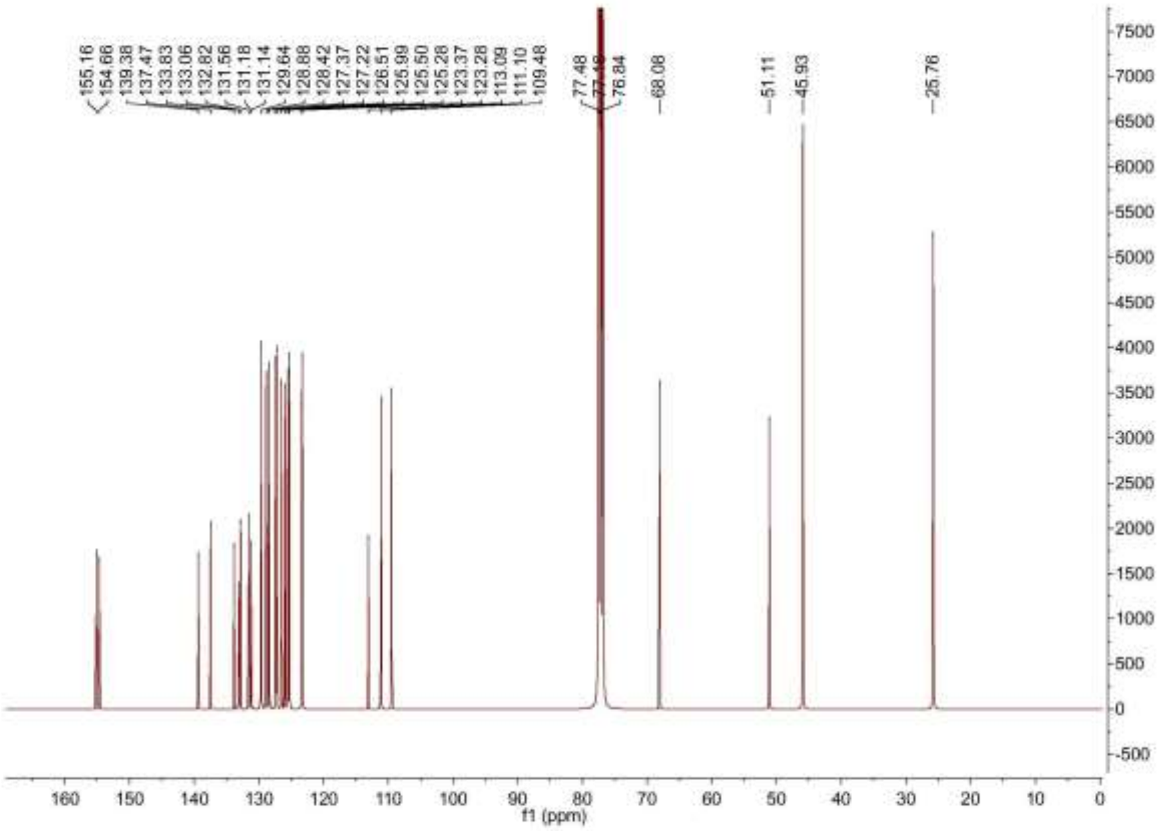

***Figure* S25.** ^{13}C-NMR spectrum of **12** (100 MHz, $CDCl_3$).

2. Docking studies

The docking studies as well as the preparation of protein and ligands were performed with Schrödinger Sofware. The BACE1 structure (PDB ID 5tol, (Wu et al. 2016, Bioorg Med Chem Lett, 26, 5729-5731) was prepared using Protein Preparation Wizard into the graphical user interface Maestro. This includes a pre-process steps such as hydrogen atoms addition, modelling of missing side chains using and removal of water molecules. Also refine for hydrogen bonds using PROPKA at pH = 7.0 (Olsson et al. 2011, J Chem Theory Comput, 7, 525-537) and restrain minimization using OPLS3e force field were performed.

Ligand preparation was carried out by LigPrep panel into Maestro. The force field selected was OPLS3e. For generation of possible tautomers and ionization states was used Epik at pH = 7.0.

The grid was centered on the binding site identified using the program implemented in the PLIDflow workflow (Ulzurrun et al. 2020, Bioinformatics, 36, 4203-4205) AutoLigand (Harris et al. 2008, Proteins, 70, 1506-1517). The docking simulation was performed using Glide.

In order to explain the activity of this family of derivatives and specifically the influence of the piperidinopropyl chain, the docking study of the compound pair **31** and **4** was carried out with the GLIDE program (*Fig.* S26).

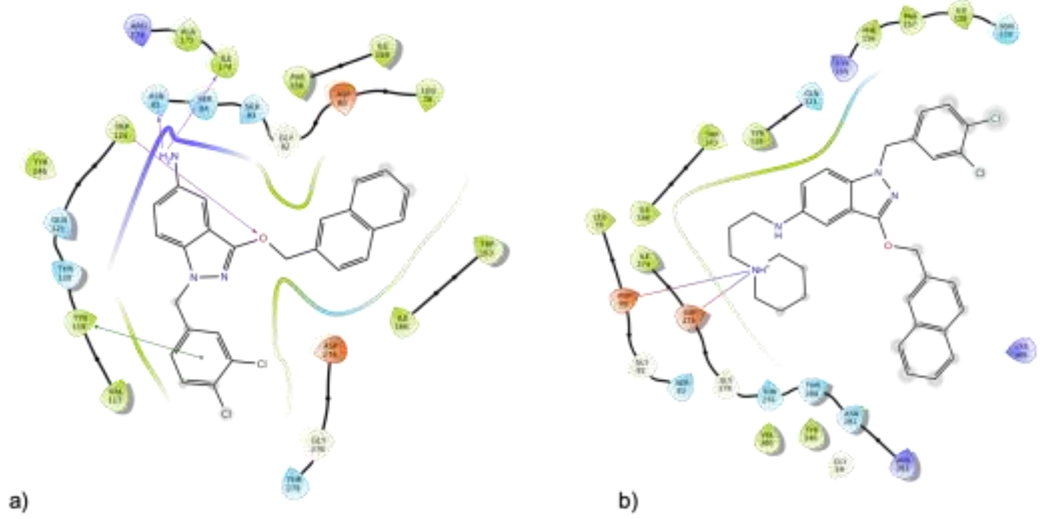

Figure S26. 2D interaction plot of compounds (a) **31** and (b) **4.**

The docking simulation revealed the interactions gathered in *Table S3.* Analyzing *Fig.* S26 and *Table S3*, we can see the influence of the piperidinopropyl chain. As can be seen, in the derivative **31**, the amino group, form a hydrogen bond with Asn85, but does not establish interactions with the catalytics Asp80 and Asp276 while the piperidinopropyl chain in the compound **4** favours the relocation of the compound to allow to form two ionic bridges between the amino group of piperidinopropyl chain and the catalytic aspartic acids Asp80 and

Asp276. In addition, to the interaction with the catalytic aspartic acids, the compound **4** establishes interaction with different subsites (SS) defined according to Hu et al. (ACS Chem Neurosci, 2019, 10, 880-889). The group of Hu performed a new dissection of the binding pocket of BACE1 from a systematic analysis of the results of 354 ligand-protein complexes. Thus, eight subsites, including the catalytic site were identified. Well, our compound **4** establishes interactions with three subsites (SSI, SS3 and SS4) by aromatic H-bond and halogen bond interactions thus explaining its biological activity.

Table S3. Interactions of **31** and **4** in the binding site of BACE1.

**Res.**	**31**	**4**	**Subsite**
Asp80	-	$-NH^{+}$/ salt Bridge	Catalytic Site (SS2)
Asp276	-	$-NH^{+}$/ salt Bridge	Catalytic Site (SS2)
Asn85	NH_2/Hydrogen bond	-	-
Tyr119	Phenyl/Π-Π	-	SS1
Trp124	-O-/Hydrogen bond	-	SS8
Lys155	-	Indazolyl/Aromatic H-bond<br>Phenyl/Aromatic H-bond	SS3
Phe156	-	Indazolyl/Aromatic H-bond	SS1
Asn159	-	Phenyl/Halogen bond	-
Ile174	NH_2/Hydrogen bond	-	SS8
Gly278	-	Indazolyl/Aromatic H-bond	SS4
Arg355	-	Naphthyl/Aromatic H-bond	-

Elemental Analysis Data

1-benzyl-3-(1-naphthylmethoxy)-5-(3-piperidinopropylamino)indazole (**1**).

Anal. ($C_{33}H_{36}N_4O$, 504.67) % calcd. (% found) C: 78.54 (78.61); H: 7.19 (7.14); N: 11.10 (11.03).

3-(benzyloxy)-1-(2,3-dichlorobenzyl)-5-(3-piperidinopropylamino)indazole (**2**).

Anal. ($C_{29}H_{32}Cl_2N_4O$, 523.50) % calcd. (% found) C: 66.54 (66.43); H: 6.16 (6.25); N: 10.70 (10.60).

1-(3,4-dichlobenzyl)-3-(1-naphthylmethoxy)-5-(3-piperidinopropylamino)indazole (**3**).

Anal. ($C_{33}H_{34}Cl_2N_4O$, 573.56) % calcd. (% found) C: 69.10 (68.85); H: 5.98 (6.21); N: 9.77 (9.61).

1-(3,4-dichlobenzyl)-3-(2-naphthylmethoxy)-5-(3-piperidinopropylamino)indazole (4)

Anal. ($C_{33}H_{34}Cl_2N_4O$, 573.56) % calcd. (% found) C: 69.10 (69.05); H: 5.98 (6.16); N: 9.77 (9.65).

3-(2,3-dichlorobenzyloxy)-1-(1-naphthylmethyl)-5-(3-piperidinopropylamino)indazole (**5**).

Anal. ($C_{33}H_{34}Cl_2N_4O$, 573.56) % calcd. (% found) C: 69.10 (69.28); H: 5.98 (5.92); N: 9.77 (9.92).

1-(2-naphthylmethyl)-3-(2-naphthylmethoxy)-5-(3-piperidinopropylamino)indazole (**6**).

Anal. ($C_{37}H_{38}N_4O$, 554.72) % calcd. (% found) C: 80.11 (80.01); H: 6.90 (7.08); N: 10.10 (9.95).

1-benzyl-3-benzyloxy-5-(2,3-dichlorobenzamido)indazole (7).

Anal. ($C_{33}H_{34}Cl_2N_4O$, 573.56) % calcd. (% found) C: 69.10 (69.28); H: 5.98 (5.92); N: 9.77 (9.92).

1-benzyl-3-(1-naphthylmethoxy)-5-((N,N-diethylcarbamoyl)amino)indazole (**8**).

Anal. ($C_{30}H_{30}N_4O_2$, 478.58) % calcd. (% found) C: 75.29 (75.01); H: 6.32 (6.63); N: 11.71 (11.82).

1-(4-chlorobenzyl)-3-(1-naphthylmethoxy)-5-((N-methyl,N-phenylcarbamoyl)amino)indazole (9).

Anal. ($C_{33}H_{27}ClN_4O_2$, 547.05) % calcd. (% found) C: 72.45 (72.49); H: 4.97 (5.06); N: 10.24 (10.15).

1-(3,4-dichlorobenzyl)-3-(1-naphthylmethoxy)-5-((N,N-diphenylcarbamoyl)amino)indazole (10).

Anal. ($C_{38}H_{28}Cl_2N_4O_2$, 643.56) % calcd. (% found) C: 70.90 (70.92); H: 4.25 (4.39); N: 9.04 (8.71).

3-benzyloxy-1-(2,3-dichlorobenzyl)-5-(((4-methylpiperazino)carbonyl)amino)indazole (11).

Anal. ($C_{27}H_{27}Cl_2N_5O_2$, 524.44) % calcd. (% found) 61.84 (61.66); H: 5.19 (4.98); N: 13.35 (13.48).

3-(2,3-dichlorobenzyloxy)-1-(1-naphthylmethyl)-5((1-pyrrolidinyl-carbonyl)amino)indazole (12).

Anal. ($C_{30}H_{26}Cl_2N_4O_2$, 545.50) % calcd. (% found) 66.06 (65.82); H: 4.80 (4.92); N: 10.27 (10.11).

1-(4-chlorobenzyl)-5-nitro-3-indazolol (17).

Anal. ($C_{14}H_{10}ClN_3O_3$, 303.70) % calcd. (% found) C: 55.37 (55.41); H: 3.32 (3.42); N: 13.84 (13.82).

1-(2,3-dichlorobenzyl)-5-nitro-3-indazolol (18).

Anal. ($C_{14}H_9Cl_2N_3O_3$, 338.15) % calcd. (% found) C: 49.73 (49.36); H: 2.68 (2.74); N: 12.43 (12.29).

1-(3,4-dichlorobenzyl)-5-nitro-3-indazolol (19).

Anal. ($C_{14}H_9Cl_2N_3O_3$, 338.15) % calcd. (% found) C: 49.73 (49.49); H: 2.68 (2.82); N: 12.43 (12.28).

1-(1-naphthylmethyl)-5-nitro-3-indazolol (20).

Anal. ($C_{18}H_{13}N_3O_3$, 319.31) % calcd. (% found) C: 67.71 (67.51); H: 4.10 (4.05); N: 13.16 (12.96).

3-(benzyloxy)-1-(2,3-dichlorobenzyl)-5-nitroindazole (21).

Anal. ($C_{21}H_{15}Cl_2N_3O_3$, 428,27) % calcd. (% found) C: 58.89 (58.87); H: 3.53 (3.54); N: 9.81 (9.98).

3-(2,3-dichlorobenzyloxy)-1-(1-naphthylmethyl)-5-nitroindazole (22).

Anal. ($C_{25}H_{17}Cl_2N_3O_3$, 478.33) % calcd. (% found) C: 62.77 (61.56); H: 3.58 (3.76); N: 8.78 (8.53).

1-(3,4-dichlorobenzyl)-3-(2-naphthylmethoxy)-5-nitroindazole (23).

Anal. ($C_{25}H_{17}Cl_2N_3O_3$, 478.33) % calcd. (% found) C: 62.77 (62.51); H: 3.58 (3.62); N: 8.78 (8.68)

1-benzyl-3-(1-naphthylmethoxy)-5-nitroindazole (24).

Anal. ($C_{25}H_{19}N_3O_3$, 409.44) % calcd. (% found) C: 73.34 (73.26); H: 4.68 (4.70); N: 10.26 (10.29).

1-(4-chlorobenzyl)-3-(1-naphthylmethoxy)-5-nitroindazole (25).

Anal. ($C_{25}H_{18}ClN_3O_3$, 443.88) % calcd. (% found) C: 67.65 (67.72); H: 4.09 (3.97); N: 9.56 (9.39).

1-(3,4-dichlorobenzyl)-3-(1-naphthylmethoxy)-5-nitroindazole (26).

Anal. ($C_{25}H_{17}Cl_2N_3O_3$, 478.33) % calcd. (% found) C: 62.77 (62.41); H: 3.58 (3.56); N: 8.78 (8.63)

5-amino-3-(2-naphthylmethoxy)-1-(2-naphthylmethyl)indazole (28).

Anal. ($C_{29}H_{23}N_3O$, 429.50) % calcd. (% found) C: 81.09 (80.42); H: 5.40 (5.53); N: 9.78 (9.61).

5-amino-3-(benzyloxy)-1-(2,3-dichlorobenzyl)indazole (29).

Anal. ($C_{21}H_{15}Cl_2N_3O_3$, 398.30) % calcd. (% found) C: 62.51 (62.75); H: 3.93 (4.32); N: 10.94 (10.51).

5-amino-3-(2,3-dichlorobenzyloxy)-1-(1-naphthylmethyl)indazole (30).

Anal. ($C_{25}H_{19}Cl_2N_3O$, 448.34) % calcd. (% found) C: 66.97 (66.69); H: 4.27 (4.16); N: 9.37 (9.36).

5-amino-1-(3,4-dichlorobenzyl)-3-(2-naphthylmethoxy)indazole (31).

Anal. ($C_{25}H_{19}Cl_2N_3O$, 448.34) % calcd. (% found) C: 66.97 (66.75); H: 4.27 (4.33); N: 9.37 (9.50).

5-amino-1-benzyl-3-(1-naphthylmethoxy)indazole (32).

Anal. ($C_{25}H_{21}N_3O$, 379.45) % calcd. (% found) C: 79.13 (78.80); H: 5.58 (5.63); N: 11.07 (10.97).

5-amino-1-(4-chlorobenzyl)-3-(1-naphthylmethoxy)indazole (33).

Anal. ($C_{25}H_{20}ClN_3O$, 413.9) % calcd. (% found) C: 72.16 (72.55); H: 5.02 (4.87); N: 10.12 (10.15).

5-amino-1-(3,4-dichlorobenzyl)-3-(1-naphthylmethoxy)indazole (34).

Anal. ($C_{25}H_{19}Cl_2N_3O$, 448.34) % calcd. (% found) C: 66.97 (66.59); H: 4.27 (4.22); N: 9.37 (9.42).
